# Convergence and divergence of DNA methylation and gene expression patterns in neopolyploid *Arabidopsis kamchatica*

**DOI:** 10.1038/s41467-026-75042-4

**Published:** 2026-06-30

**Authors:** Stefan Milosavljevic, Dario Copetti, Kenji Yip Tong, Aki Morishima, Jun Sese, Mark D. Robinson, Kentaro K. Shimizu, Rie Shimizu-Inatsugi

**Affiliations:** 1 Department of Evolutionary Biology and Environmental Studies, University of Zurich, Zurich, Switzerland; 2 Arizona Genomics Institute, School of Plant Sciences, University of Arizona, Tucson, AZ USA; 3 Humanome Lab., Inc, Tokyo, Japan; 4 Department of Molecular Life Sciences, University of Zurich, Zurich, Switzerland; 5 Kihara Institute for Biological Research, Yokohama City University, Yokohama, Japan

**Keywords:** Experimental evolution, Plant evolution, DNA methylation, Evolutionary genetics

## Abstract

Polyploidization is a key evolutionary force in plants, but the reasons behind its prevalence remain unclear. While the potential ecological benefits of established polyploids are well studied, little is known about the short-term genomic and epigenetic responses immediately after polyploidization, which are crucial for successful speciation. In this study, we assemble the genomes of the two progenitors of *Arabidopsis kamchatica* (*A. halleri* and *A. lyrata*) and examine the epigenome of synthetic and natural tetraploids of *A. kamchatica* to investigate the combined effect of allopolyploidization and environment on DNA methylation changes. We find the most significant methylation changes at allopolyploidization, followed by smaller changes in subsequent generations. Offspring grown under different conditions show divergent patterns, suggesting environmental effects, while their methylation patterns converge toward those of natural tetraploids over generations. Our findings highlight two key epigenetic changes post-polyploidization: convergence toward established polyploids and divergence driven by environmental factors.

## Abstract (web summary)

Genomic and epigenetic responses immediately after plant polyploidization are crucial for successful speciation. Here, the authors assemble the genomes of two progenitors of tetraploid *Arabidopsis kamchatica* and reveal the role of allopolyploidization and environment on DNA methylation changes.

## Introduction

Allopolyploidization, the merging of two genomes to produce a new genotype, is one of the most profound genome reorganization opportunities in evolution. Ancient polyploidization events contributed to the diversity of many successful plant and animal lineages, such as angiosperms and vertebrates^1–6^. In plants, polyploidization events are ubiquitous^7^ *in natura* as well as in many important cultivated crops being polyploids, highlighting the evolutionary and agronomic prevalence of polyploidy^8,9^. In particular, the merger of two closely-related but ecologically diverged genomes can form an allopolyploid with novel genomic and phenotypic properties^5,10^. This could result in a wider habitat range than either parent, potentially extending to an entirely new habitat^10^.

When allopolyploidization occurs, epigenetic and genetic responses are assumed to be the key to successful adaptation and speciation. Studies of many genera revealed various patterns and levels of genetic and epigenetic changes after polyploidization. For example, newly polyploidized *Brassica* and *Spartina* showed drastic genetic and epigenetic modifications, respectively^11–13^, also referred to as genome shock^14^. On the other hand, *Senecio, Arabidopsis suecica, Aegilops*, and *Mimulus* exhibited limited, but not negligible, epigenetic changes^15–19^. The picture is further complicated when accounting for the link between epigenetic and genetic changes, often including transcriptomics. In some species, DNA methylation changes in transposable elements (TEs) could have a causal role in explaining gene silencing^15^. This is not the case for *A. suecica*, where no abnormal TE activity was detected^17^, while gene-associated methylation changes correlated with transcriptional changes^20^.

The findings described above mostly relied on experiments where polyploids were grown in optimal laboratory conditions. Since polyploids evolve in natural conditions with a multitude of biotic and abiotic stresses, investigating the effects of the environment should contribute to filling up the current knowledge gap. The response(s) to these environmental factors were hypothesized to be a critical or even essential element in the establishment of a polyploid^21^. Considering that adaptation to new niches is part of the speciation process of polyploids^5^, it is crucial to investigate the extent to which the environment can shape the epigenetic change of neopolyploids. Although many research focused on the epigenetic status at a specific time point, capturing the transition over generations should be another important factor to be addressed more in-depth.

Among many ecologically important polyploid species, *A. kamchatica* is a suitable model species to study ecological adaptation after allopolyploidization due to its wider latitudinal distribution compared to the progenitors. The progenitors, *A. halleri* and *A lyrata*, are closely related species with the same number of chromosomes but have clear differences in geographical distribution as well as preferred soil type, *A. halleri* being a well-studied metal-ion hyperaccumulator^10^. The genome-wide pattern of polymorphism and selection of *A. kamchatica* was studied^22^ while epigenetic evolution and its role in adaptation to various habitats remain unexplored. An artificial neotetraploid *A. kamchatica* can be resynthesized in the lab to serve as a model for studying the epigenetic change at the early stage after polyploidization, as well as the effects of the environment.

In this study, we investigate the impact of allopolyploidization on DNA methylation and its generational transitions by comparing whole-genome DNA methylation patterns among progenitors, artificial neotetraploids, and natural tetraploids, as well as between neotetraploids incubated under two different conditions. The DNA methylation patterns of neotetraploids show convergent trends toward those of natural tetraploids over generations, whereas the neotetraploids diverge under two conditions. Similar trends are found in gene expression patterns more pronouncedly. These results suggest two aspects of neopolyploid evolution: convergence toward established polyploids and divergence driven by environmental factors, which may shape the intraspecific epigenetic and transcriptomic diversity of polyploid species. At last, we discuss the relationship between whole-genome DNA methylation and gene expression patterns.

## Results

### Genome assemblies of two progenitors

**Fig. 1:**
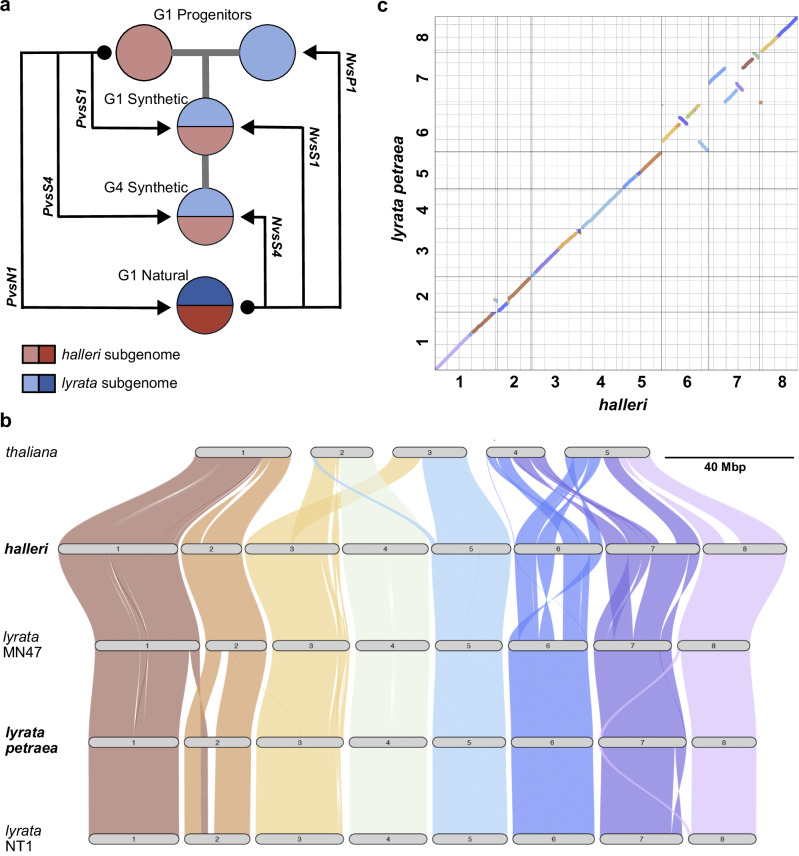
Experimental design and the features of the *A. halleri* and *A. lyrata* genomes. **a** In our experimental design, progenitor species *A. halleri* (pale red circle) and *A. lyrata* (pale blue circle) were grown together with a synthetic *A.kamchatica* (pale red and pale blue semicircles) and two natural *A. kamchatica* lines (red and blue semicircles, ALK and TKS) in two different conditions, mild and stress. The methylation pattern was compared between the samples suggested by the arrows, with the black dots suggesting a reference. Arrows on the left side indicate the comparison with progenitors as references, and those on the right side with natural polyploids as references. These comparisons were conducted for each subgenome. **b** Genomic synteny of *A. thaliana* (Columbia), *A. halleri*, *A. lyrata* subsp. *lyrata* MN47^26^, subsp. *petraea*, and accession NT1^27^. The names of the lines we used in the present study are highlighted in bold. **c** Collinearity plot of *A. lyrata* subsp. *petraea* and *A. halleri* genomes. Numbers represent the chromosomes.

We used *A. halleri* subsp. *gemmifera* and *A. lyrata* subsp*. petraea*, which were the taxa closest to the subgenomes of natural *A. kamchatica*^22^, to synthesize artificial tetraploid lines (termed synthetics here). These neopolyploids produced self-fertilized seeds, indicating they were self-compatible. The offsprings from the same selfed synthetic tetraploid plants were divided into two groups incubated in two different conditions, named mild and stress conditions for 4 generations (Supplementary Fig. 1). The two progenitors and two genetically distinct natural tetraploids from Alaska (ALK) and Japan (TKS), which were shown to have originated from independent polyploidization events^22–24^, were also cultivated together with the synthetic line. ALK and TKS are collected at cold and hot habitats of the broad distribution range of *A. kamchatica*, respectively^22^. Whole genome methylation patterns in all individuals from first (G1) and fourth (G4) generations (G5 for TKS) were analyzed by bisulfite sequencing (BS-seq) (see Fig. 1a for schematic comparisons).

The survival rate of the synthetic polyploids in the stress condition, which was harsher with the maximum temperature of 26 °C, was approximately 80% (Supplementary Table 1), while 100% for other species. The mild condition is a suitable environment for all three species, with the maximum temperature of 22 °C, resulting in 100% survival rates of all species. Next, we examined the seed production as a proxy of reproductive success. Seed production among all synthetics across generations was significantly different between conditions, better in mild conditions (Supplementary Table 1). In addition, in the stress condition, not all individuals of a natural tetraploid ALK derived from a colder habitat produced seeds, while all individuals of the other natural tetraploid, TKS, produced seeds on both conditions. These data suggest the difference in tolerance to the stress condition among natural genotypes. Overall, the fitness of synthetics was affected by the abiotic stress in the stress condition.

For DNA methylation analysis, we assembled chromosome-level reference genomes of both *A. lyrata* and *A. halleri* used to generate synthetics by highly accurate PacBio HiFi reads. The resulting assemblies were highly contiguous with N50 of about 28 and 26 Mb, with 12 and 42 gaps per genome, respectively. Both genome assemblies had BUSCO scores of >99% (Table 1). The assembly of *A. halleri* was significantly improved in terms of contiguity compared with previous assemblies^22,25^. The quality of the assembly of *A. lyrata* subsp. *petraea* is comparable to the previously reported assemblies of MN47^26^ and NT1^27^. The difference between *A. lyrata* subsp. *petraea* and MN47 at chromosomes 1 and 2 were also supported by NT1, suggesting the intraspecific structural variations in *A. lyrata* (Fig. 1b). The synteny between our *A. halleri* and *A. lyrata* assemblies was generally high, except for chromosomes 6 and 7 (Fig. 1b, c), which could be improved by incorporating additional types of sequencing data, such as structural information from Hi-C sequencing, in the future. We suggest that these assemblies with the high BUSCO values are adequate for the epigenetic analysis of this study focusing on the genic level.

**Table 1:** Statistics on the quality of the two progenitor genomes and assemblies

	*A. halleri*	*A. lyrata*
Total length of assembly (bp)	246,097,870	206,717,915
Number of contigs	302	135
Scaffold mean length (bp)	814,894	1,531,244
Longest scaffold (bp)	37,868,808	28,718,688
Shortest scaffold (bp)	14,180	13,472
Total gaps	12	42
N90	19,340,014	20,443,964
N90 contig	8	8
Chloroplast contigs	1	1
Mitochondrion contigs	4	1
Complete BUSCOs	99.10%	99.10%
Single copy BUSCOs	92.10%	96.70%
Duplicated BUSCOs	7.00%	2.40%
KAT comp assembly completeness (%)	99.64%	99.65%
Total annotated genes	29,894	27,602
TAIR 10 orthologs (one-way hit)	26,636	25,159
TAIR 10 orthologs (reciprocal best hit)	20,418	20,435
Homeologous pairs (reciprocal best hit)	21,305	21,305

**Fig. 2:**
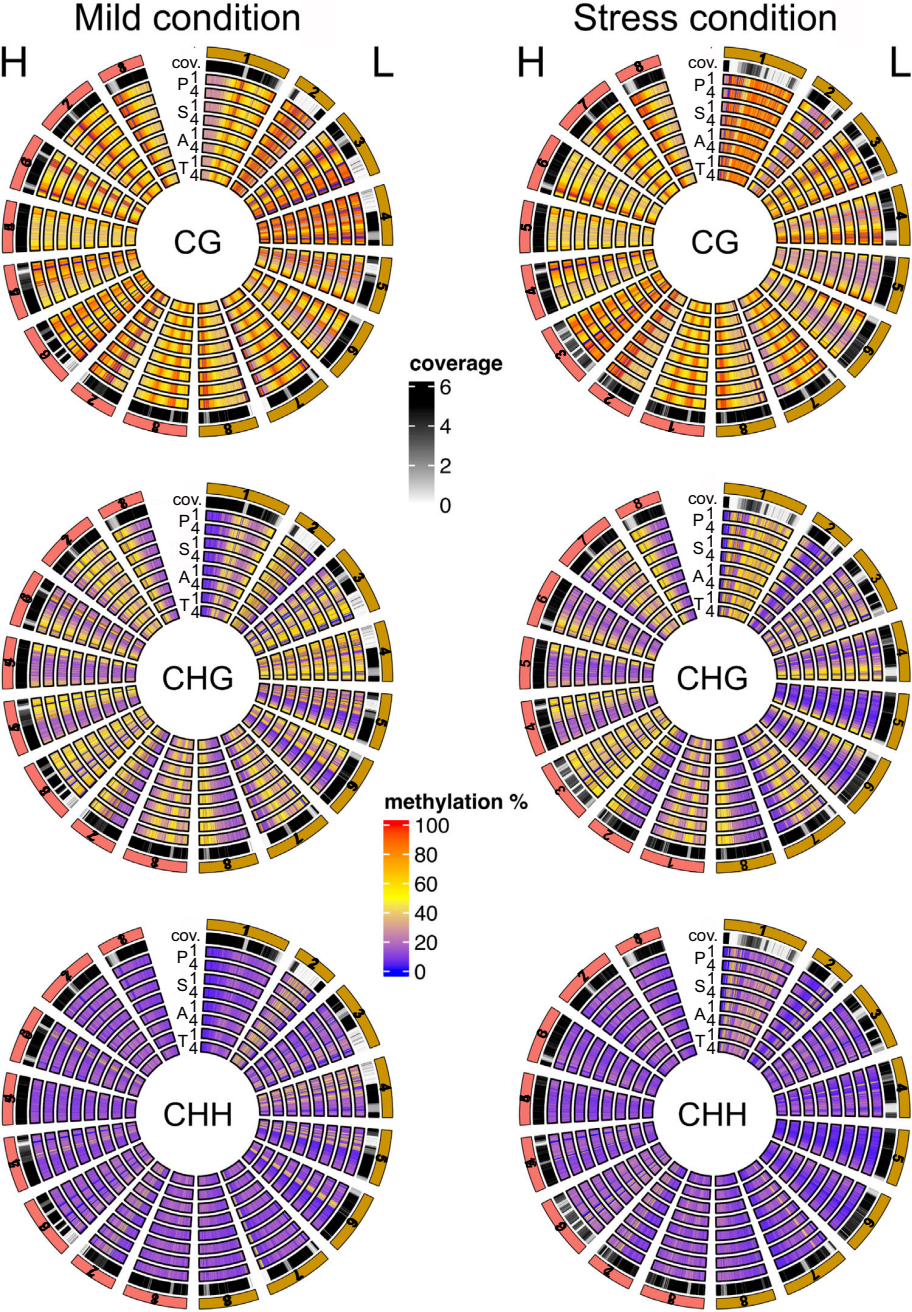
Average methylation levels along chromosomes across methylation contexts and conditions. Average methylation levels were calculated for 100 kb windows. Circos plots note from outside: chromosome number (1–8 of L-side in yellow and 1–8 of H-side in pink, clockwise from the top), cov. (mapping coverage in G4 synthetics), and average methylations (P: progenitors, S: synthetics, A: ALK, and T: TKS, in 1: G1, and 4: G4/5).

While the mapping coverage of BS-seq short reads on most chromosomes showed a rather uniform pattern, some regions showed extremely low coverage on the *A. lyrata*-subgenome (L) in all synthetic samples (cov. in Fig. 2 and Supplementary Fig. 2), which is also supported by the mapping of normal DNA seq without BS-treatment (Supplementary Fig. 3). These stretches could represent either chromosome loss or subgenome dosage change due to homoeologous exchange (HE), both previously reported in other polyploid systems^28^. Considering the unreduced genome size of their descendants measured by flow cytometry (Supplementary Table 2) and the higher coverage of *A. halleri-*subgenome (H) at the counterpart regions of these L regions with lower coverages (Supplementary Figs. 2 and 3), we suggest that dosage change from L to H due to HE occurred at these regions. In natural tetraploids ALK and TKS, we did not detect a sign of HE by coverage (Supplementary Fig. 4). We defined HE regions by comparing *A. halleri* and *A. lyrata* coverage (indicated by the pink bar in Supplementary Fig. 2). The proportion of HE region among the whole genome was estimated as 5% and 2% in mild and stress conditions, respectively (Supplementary Table 2). To focus on the effect of allopolyploidization on methylation, we excluded these HE regions in further analysis (see “Methods” section) by filtering out low-coverage regions.

### Convergence of neotetraploids toward natural tetraploids

**Fig. 3:**
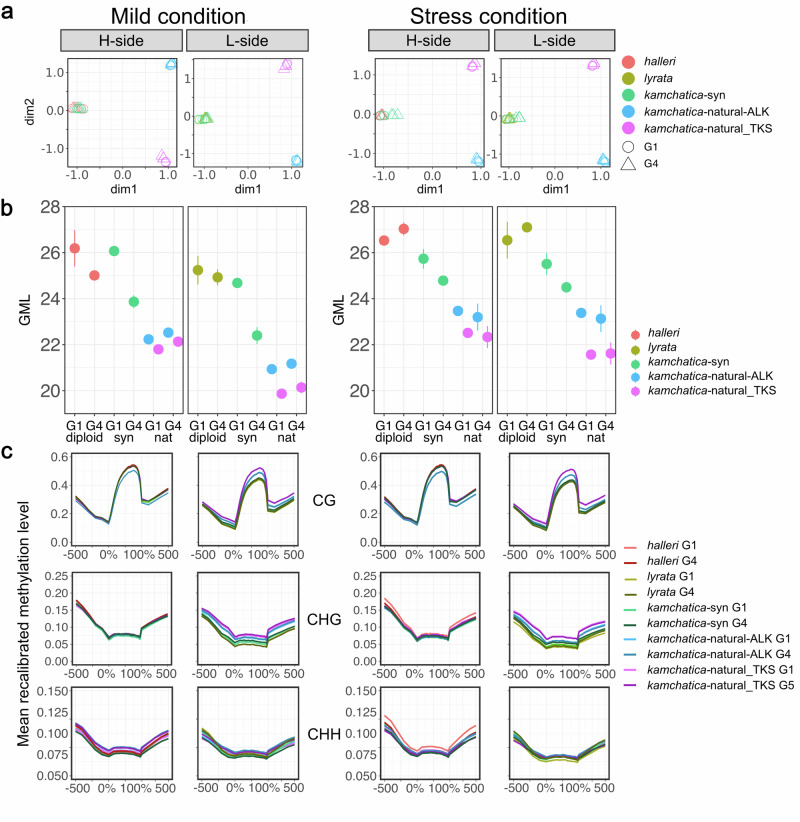
Global methylation patterns of all samples in each subgenome and in mild and stress conditions. **a** MDS plots showing the relationship of whole genome DNA methylation patterns using the top 10,000 most variable cytosines in two conditions (left: mild and right: stress) and in two subgenomes. **b** Global methylation levels (GML, %) in G1 and G4, in the same layout as in (**a**). Data presented as mean ± SD of triplicates. **c** Mean recalibrated methylation level (0–1) within gene bodies (0–100%) and around gene bodies (±500 bp) in the same layout as in (**a**). Three contexts, CG, CHG, and CHH, is shown from top to bottom.

To visualize the relationship of whole genome methylation patterns among samples, we used multidimensional scaling (MDS) on most variable shared cytosines in each subgenome (Fig. 3a). In both conditions and in both subgenomes, a similar pattern with three main clusters was found: one with progenitors and synthetics of both generations, one with ALK, and another with TKS, highlighting the similarity between progenitors and synthetics compared to the divergence of natural polyploids. The synthetic G4 was less overlapping with progenitors compared to G1. Similar patterns were found when various numbers of the most variable cytosines were considered (Supplementary Fig. 5).

By overviewing the global methylation levels (GMLs), the two progenitors had similar levels at approximately 26%, but those of ALK and TKS were lower (Fig. 3b). Their GMLs were similar between generations, but synthetics in G4 showed a decrease from G1 for both mild and stress conditions, suggesting convergence from progenitor levels toward natural lines’ levels. Overall the GMLs were slightly higher in the stress condition in any sample. Within and around the gene body, the methylation levels in CG, CHG, and CHH contexts were similar in each subgenome/genome, implying that higher changes in intergenic regions should have contributed to the change of GMLs in synthetics (Fig. 3c).

### Divergence between neotetraploids by environments

**Fig. 4:**
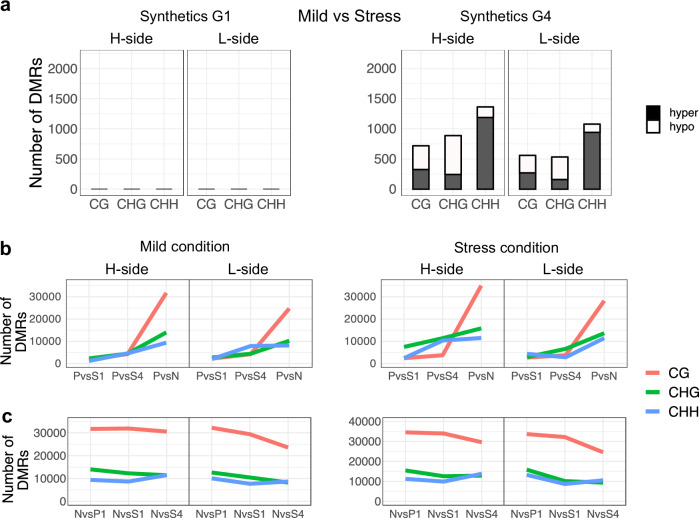
Differential methylation patterns of synthetics in each subgenome and in mild and stress conditions. **a** Number of DMRs between synthetics in two conditions at G1 (left) and G4 (right). Hypermethylation refers to the methylation increase in stress condition. **b** Number of DMRs between progenitors and synthetics G1 (PvsS1), synthetics G4 (PvsS4), or natural ALK line (PvsN) across different methylation contexts in mild (left) and stress (right) conditions. **c** Number of DMRs between natural ALK line and progenitors G1 (NvsP1), synthetic G1 (NvsS1), or synthetic G4 (NvsS4) across different methylation contexts in the same layout as in (**b**). Source data are provided as a Source data file.

To analyze the effect of environment on methylation pattern, we compared the methylation pattern of the synthetic polyploids in the mild and stress conditions. At G1, we did not find any statistically significant DMR. We found thousands at G4, suggesting a diverging pattern between the two conditions over time (Fig. 4a). We examined the DMR-associated genes (DMGs), i.e., the genes overlapping with DMRs at G4. There were 181, 255, and 201 DMGs for CG, CHG, and CHH context, respectively, at G4 on H-side, and 167, 156, and 126 DMGs on L-side. By Gene Ontology (GO) enrichment analysis on each context, we did not find enrichment in temperature-related categories in either group, potentially due to the small sample sizes. Then we combined the DMGs from three categories in each subgenome to target 225 and 164 DMGs with reciprocal best-hit genes in *A. thaliana* for the H- and L-sides, respectively, by filtering out overlaps. We found in total 46 GO categories (10 on H-side and 36 on L-side), among which GO:0009408 (response to heat, *p* = 0.045) on L-side was directly relevant to the difference of conditions reflecting the temperature difference.

To further investigate the trajectory of these environment-specific patterns in synthetic polyploids, we compared DNA methylation patterns of synthetic polyploids to progenitors at each condition and assessed their transition over generations. The number of DMRs between progenitors and synthetic polyploids increased from G1 to G4 (Fig. 4b and Supplementary Fig. 6a). This increase occurred across almost all contexts of CG, CHG, and CHH in both conditions on both subgenomes (Supplementary Fig. 6b), supporting rapidly and progressively diverging methylome patterns between progenitors and synthetic polyploids after polyploidization. Many of the DMRs found in G1 were also found in G4 (28–74%, Supplementary Table 4), suggesting the accumulation of DMRs over generations rather than a major stochastic reshuffling at each generation. More DMRs were detected on the H-side at G4 under stress conditions compared with mild conditions, and vice versa on the L-side, implying an independent effect of the environment on each subgenome.

Compared to the synthetic polyploids, progenitors and natural polyploid lines showed lower numbers of DMRs between conditions or generations (Supplementary Fig. 7), suggesting the stability of their methylome. In addition, the divergence between the two natural polyploids was not small and only slightly less than the DMRs between synthetic and natural polyploids (Supplementary Fig. 6d), indicating the influence of independent evolution at their respective habitats as well as the distinct progenitors’ genotypes at polyploidizations.

**Fig. 5:**
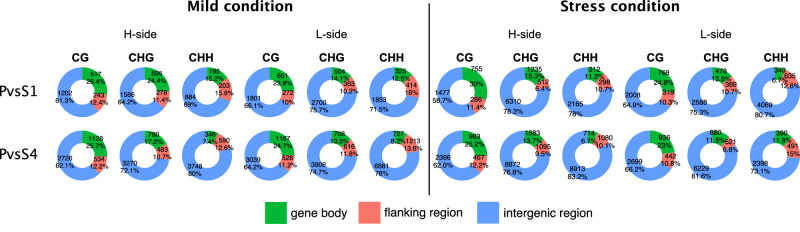
Proportions of DMRs in three genomic regions. Proportions were computed for each condition (mild and stress) and subgenome (H- and L-side), and methylation context (CG, CHG, and CHH). The first and second rows are for DMRs found when comparing progenitors to the first (PvsS1) and fourth (PvsS4) generations of synthetics. Gene bodies were defined as regions between the transcriptional start and end site of genes, flanking regions as 500 bp regions at both sides of the gene body, and intergenic regions as the remaining part of the genome. In the assembled *A. halleri* genome, flanking regions represented 19.5% of the total length, together with 55.2% of gene bodies and 25.3% of intergenic regions. For *A. lyrata*, flanking regions represented 20% of the total length, together with 56.7% of gene bodies and 23.3% of intergenic regions.

We categorized the DMRs between progenitors and synthetics in Fig. 4b into three functional genic regions: gene body, flanking region, or intergenic region (Fig. 5 and Supplementary Fig. 8). Proportions were generally similar across contexts: the majority (~66–80%) of DMRs were found in intergenic regions, while DMRs in gene bodies or flanking regions represented ~20–33%. When we focused on the DMGs, i.e., the genes overlapping with DMRs detected in gene bodies and flanking regions in each context (Supplementary Fig. 9), to detect overrepresented gene functions by GO enrichment analysis, most GOs were related to general metabolism, but only a limited number of environment tolerance-related GOs were detected both in G1 and G4. In addition, many GOs related to metal and other ions transport were detected on the L-side, both in G1 and G4, in both conditions in most contexts, and many GOs related to metal and other ions homeostasis on the H-side in many contexts (summarized in Supplementary Table 5).

To further evaluate the progress of the methylation patterns in the synthetics, we compared the number of DMRs of three samples (G1 progenitors, G1, and G4 synthetic polyploids, in the order of time) to natural polyploid lines. We found a progressive decrease in CG DMRs in both conditions (Fig. 4c and Supplementary Fig. 6a, c). This decrease was more pronounced on the L-side than on the H-side, but was not always observed in CHG and CHH contexts. The results using two natural polyploid lines (ALK, TKS) showed a very similar pattern (Fig. 4c and Supplementary Fig. 6a, c), suggesting that the methylation patterns of the synthetic polyploids became gradually closer to those of natural polyploid lines.

### Association between DNA methylation and gene expression pattern

**Fig. 6:**
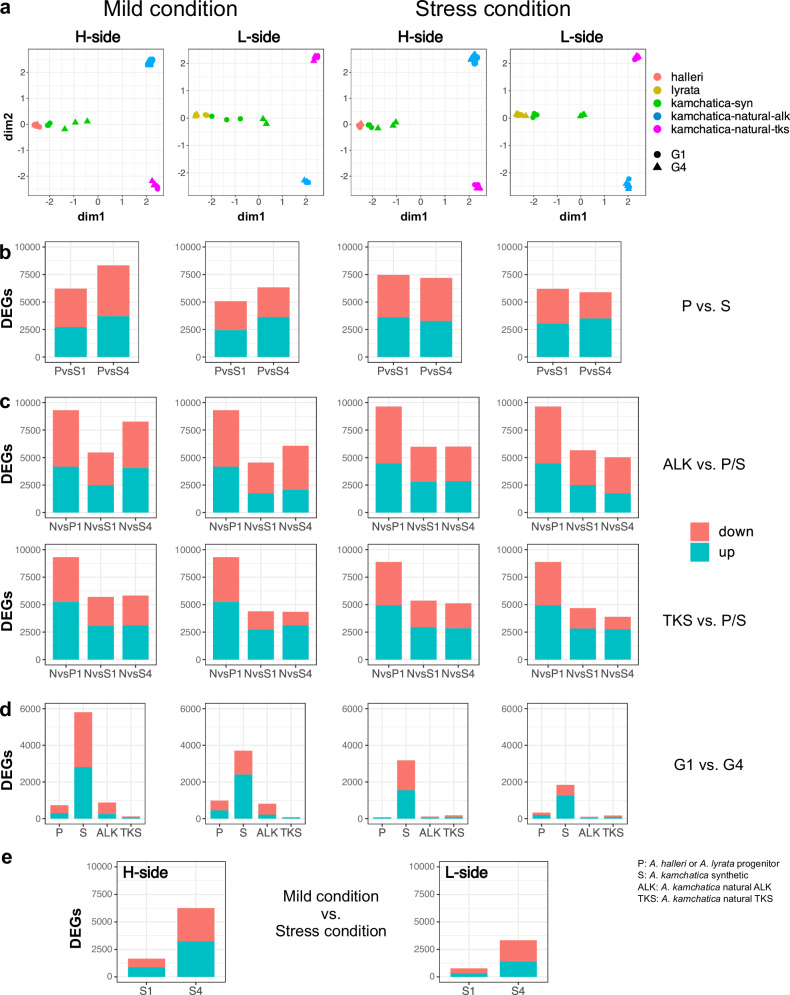
Gene expression patterns and DEG numbers in mild and stress conditions. **a** BCV plots of gene expression patterns across all samples in two conditions and in two subgenomes. The layout of the four panels is the same for (**b**–**d**). **b** Number of DEGs in synthetics G1 and G4 compared to progenitors G1 (PvsS1 and PvsS4). **c** Number of DEGs in progenitors G1 (NvsP1), synthetics in G1 (NvsS1), and synthetics G4 (NvsP4) compared to natural lines G1 (top: ALK and bottom: TKS). **d** Number of DEGs between G1 and G4 of progenitors (P), synthetics (S), and natural line (ALK), and between G1 and G5 of TKS. **e** Number of DEGs between the synthetics in mild and stress conditions (left: H and right: L) at G1 (S1) and G4 (S4) in each subgenome. Source data are provided as a Source data file.

**Fig. 7:**
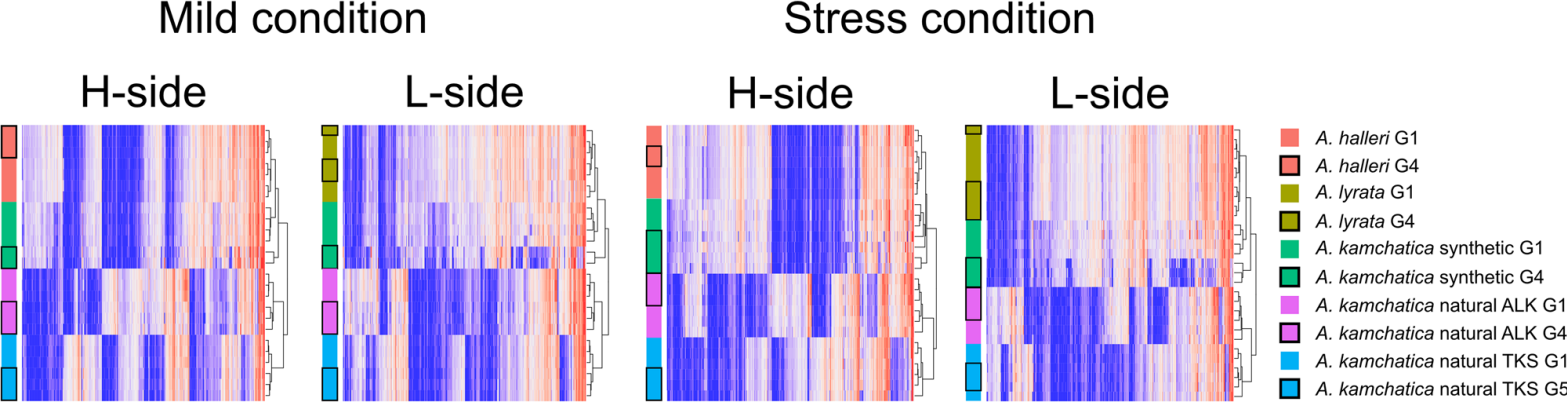
Gene expression patterns in mild and stress conditions. Heatmaps of the top 1000 most variably expressed genes across all samples in each condition (mild and stress) in each subgenome (H- and L-side). Colors indicate expression level: blue is low, white is middle, and red is high.

To compare the transcriptome pattern with the observed whole-genome DNA methylation pattern, we sequenced the RNAs from the same tissues that were used for BS-DNA seq. Clustering analyses of whole genome gene expression patterns by BCV (Fig. 6a) revealed a similar but more pronounced relationship among samples compared with the MDS plot obtained by BS-seq data (Fig. 3a), showing two distinct groups of two natural tetraploids and a looser cluster consisting of synthetic polyploids and progenitors, and the convergence of synthetics in dimension 1 towards natural lines from G1 to G4. The clustering plot using the most variably expressed 1000 genes showed a similar pattern as BCV plot (Fig. 7).

When synthetic polyploids and the progenitors were compared as we did in DMG, the number of differentially expressed genes (DEGs) represented a large proportion of all homeologous gene pairs (21,305 pairs, Table 1), approximately more than 25% in all comparison (Fig. 6b and Supplementary Fig. 10). Next, in comparison to the DMR analysis in Fig. 4c, we compared the progressive expression of three samples (G1 progenitors, G1 and G4 synthetic polyploids) to natural polyploid lines. The number of DEGs between progenitors and natural polyploid lines was the largest, but it decreased in the synthetics G1 and kept similar levels in G4 (Fig. 6c). This trend may imply an instant convergence of synthetic polyploids towards natural lines at polyploidization in the transcriptome. In contrast to the drastic changes in synthetic polyploids, no major change in gene expression pattern across generations was found in progenitors and in the two natural polyploid lines (Fig. 6d) similar to the methylation patterns (Supplementary Fig. 7). Furthermore, the number of DEGs between the synthetics in two conditions increased in G4 compared to G1 (Fig. 6e), consistent with the methylation pattern (Fig. 4a).

**Fig. 8:**
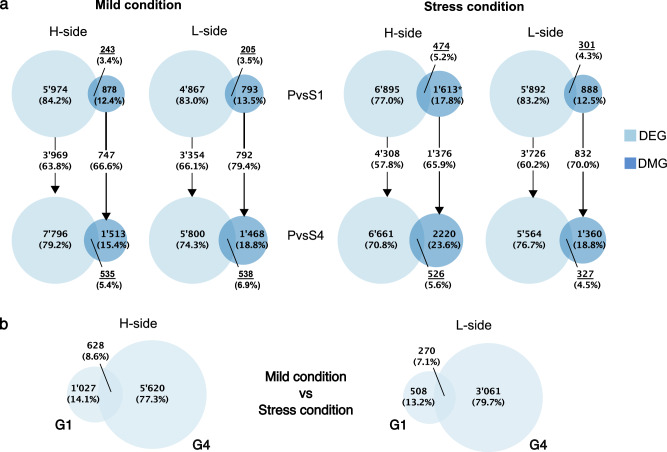
Overlap between DMGs and DEGs over generation and between conditions. **a** Number of DMGs and DEGs and their overlaps between progenitor and synthetics in two conditions (left: mild and right: stress) and two subgenomes at G1 (top) and G4 (bottom). The number of genes and the proportion (%) are indicated for each combination. Arrows with numbers connecting G1 to G4 indicate the number of conserved genes between generations and proportion (%) (G1 as reference) for DMGs and DEGs, respectively. Underlined numbers suggest the gene sets tested by GO analysis. **b** Numbers of DEGs between the synthetics in the two conditions and their overlap between G1 and G4 in each subgenome. Source data are provided as a Source data file.

To link gene expression and DNA methylation at genes, we examined the overlap between the DMGs and DEGs (Fig. 8a) by combining all DMGs across the three contexts (Supplementary Fig. 9) to compare them with DEGs. Counterintuitively, DMGs and DEGs were negatively associated, with overlaps significantly smaller than expected by chance in both conditions, as assessed by chi-square tests (Supplementary Fig. 11). This should reflect a complex correlation between DNA methylation and expression, most likely due to multiple control factors not directly related to DNA methylation. Still, we found a mild positive correlation between the methylation increase and the expression increase in these overlapping genes, implying an effect of methylation on expression (Supplementary Fig. 12). GO analysis of the overlapping genes revealed general metabolic and cellular GO terms but not environment-related terms. It was the same when the overlap of the DEGs between G1 and G4 in each condition (Fig. 8b) was analyzed.

## Discussion

We have shown a converging trend of the methylation pattern of synthetic tetraploids toward natural tetraploids. While the number of DMRs between progenitors and synthetics increased over time (Fig. 4b), the number between synthetic and natural tetraploids decreased over generations, specifically in CG context which is mostly associated with gene bodies (Fig. 4c). The GMLs of synthetic tetraploids also supported the convergence, showing the decrease of GMLs to the level of natural tetraploids from G1 to G4 in both conditions and subgenomes (Fig. 3b). In contrast, natural tetraploid lines, as well as progenitors, showed relatively stable methylation patterns over generations (Supplementary Fig. 7). Assuming that natural tetraploid lines represent a long-term stable methylation state and synthetic individuals are transitioning towards it, the transition might imply the existence of a stable point or range for DNA methylation level and pattern in the allopolyploid genome, which could be a part of diploidization process and characterized by a stable epigenetic status over generations. If so, this stabilization process could be associated with the successful establishment of a neopolyploid as a new species. Each ecological population may represent a distinct stability point, though a majority of the methylation status would be shared as seen in two contrasting populations of ALK and TKS (Fig. 3a). As the number of DMRs between progenitor and synthetics in G4 (Fig. 4b) is much greater than those between the synthetics in two conditions in G4 (Fig. 4a), we assume a stronger effect of this autonomous change toward stable points than the effect from the environment at the early stage of polyploids.

Compared to the relatively minor proportion in the genome affected by DMRs (only 1–5%, Supplementary Table 6) in G4 synthetics, the proportion of DEGs per total genes was much larger. This difference could be explained by an amplification effect by gene regulatory networks^29,30^, but we cannot exclude the effect of other (epi)genetic systems in the regulation of expression, e.g., non-coding RNAs^31^, histone modifications and chromatin structure^32^, and TEs^15^, which have not been investigated in this study. The DMRs outside genic regions could also contribute to this effect.

In addition to the larger scale of DEGs between synthetics and progenitors, the transcriptome showed a quicker pattern of convergence toward natural polyploids over generations than DMRs. This is consistent with the instant similarity of gene expression patterns reported in various contexts in polyploids: between progenitors and reciprocally produced polyploid of *Tragopogon*^33^, between two recent polyploids of *Spartina*^34^, between wild tetraploid and hybrid cottons^35^, and natural progenitors, hybrid, and synthetic polyploid of *Mimulus*^15^. This is the first example to show the further convergence over generations toward natural polyploids. These general patterns may also support the idea of epigenetic stable points in allotetraploids. This stable point might be robust against the slight intraspecific difference in progenitors’ genotypes, as many of the polyploid species are known to have multiple origins. We may refer to the process leading to it as epigenetic canalization after polyploidization^36^.

We suggested how different environmental conditions could lead to divergent whole-genome DNA methylation patterns (Figs. 3 and 4, Supplementary Fig. 6) and the gene expression patterns (Fig. 6 and Supplementary Fig. 10) in neopolyploids at the early generations after allopolyploidization. While no differences were found in G1, the epigenetic pattern in synthetics showed divergence already in G4 with a large number of DMRs between two conditions (Fig. 4a) as well as DEGs (Fig. 6e). Taken together, these results highlight higher plasticity in DNA methylation and gene expression in neopolyploids compared to diploid progenitors and established polyploids, and suggest a potential effect of environmental condition on the direction of change. This higher plasticity in DNA methylation is consistent with the previous report comparing diploid progenitor rice and a new autotetraploid, in which the higher salt tolerance of the tetraploid could be attributed to the higher induction of multiple key stress-responsive genes, most probably due to the higher plasticity of their DNA methylation in tetraploid^37^. We did not detect such a drastic change in a specific group of genes, probably because our stress could have been milder, and the relevant key responsive genes are less clear for heat stress than salt stress. In addition, as we investigated only four generations, it is still unclear how these short-term changes affect the divergence of polyploids in the longer term, as found between ALK and TKS. The speed of change at each generation should be affected by multiple reasons, including the strength of stress levels and other structural constraints.

Environmental conditions had an effect on the distribution and amount of DMRs on each subgenome in synthetic *A. kamchatica*, with significantly more DMRs on the H-side in stress conditions compared to similar levels on the L-side in two conditions (Fig. 4b and Supplementary Fig. 6a). This might be related to the difference of habitats between the two progenitors. Two ecological factors are known so far to divide the habitats of the two progenitor species. First, while *A. lyrata* is found in the Arctic Circle, *A. halleri* inhabits lower latitudes. Another difference is the strengthened heavy metal tolerance of *A. halleri* as a hyper-accumulator, frequently found on contaminated soil. The distribution of *A. kamchatica* is intermediate and partially overlapping with the progenitors’ niches^10^, and the metal-ion accumulation ability is also intermediate^38^. Their different tolerance to temperature, heavy metals, or other factors might be reflected in the difference in methylation patterns between them. Among the GO categories enriched in the DMRs detected between progenitor and synthetic tetraploid, we found many ion transport and ion homeostasis-related categories. Given that the metal-ion tolerance is one of the crucial factors between the two progenitors, this enrichment may suggest that the methylation pattern of the relevant genes might be susceptible to polyploidization due to the potential divergent methylation patterns between progenitors.

We detected HE regions from L to H subgenomes in synthetic *A. kamchatica*. Even though we have not found evidence of HE in the genome of 25 natural *A. kamchatica* accessions^22^, HE was also detected in other allopolyploids, e.g*., Brassica*^39^ and Tragopogon^40^. In another *Arabidopsis* tetraploid species, *A. suecica*, one out of 15 natural accessions was found with a small HE region^17^. The timing of these HE generations and their dynamics remain understudied. In our synthetic tetraploids, HE events may occur at either the diploid hybrid stage or following tetraploid stages, resulting in copy number ratios ranging from 0:4 to 4:0. In this study, lower DMR density was detected in such HE regions compared to non-HE regions (Supplementary Table 3), suggesting the presence and ratio of counterpart subgenomes as a potential driver of methylation pattern changes. The long-term effect of HE on our synthetic *A. kamchatica* is not clear yet, but we should note that the lower level of HE found in the stress condition may imply its negative effect on fitness under selective pressure in the long term. This speculation would be consistent with the synthetic *A. suecica* in which more HE has been found^41^, and incorrect recombination partner choice at meiosis was found to be strongly correlated with the frequency of HEs. As discussed in the previous papers on synthetic *A. suecica*^41,42^, the higher stability in meiosis, as well as fewer HEs, would be more advantageous in the long term.

While most methylation changes were found in CG and CHG contexts in *A. suecica*^20^, higher amounts of changes were found in CHG and CHH contexts in synthetic *A. kamchatica*. This difference might be caused by different genome architectures of progenitors. Previous work comparing DNA methylation patterns in *A. thaliana*, *A. lyrata*, and *C. rubella* found the expansion and reduction of repetitive sequences and TEs as the main drivers producing interspecific difference^43^. The level of synteny between progenitors’ genomes and epigenomes as a result of speciation might decide how DNA methylation is affected at polyploidization.

We investigated the trend of methylation change in synthetic *A. kamchatica* compared with progenitors by focusing on the following three features to examine how they could shape the susceptibility to change. Firstly, the distribution of DMRs along each chromosome (Supplementary Fig. 13) was similar among chromosomes, with the valleys linked to the telomeres and centromeres as well as HE regions. Second, the genetic divergence from its homeologous gene did not have a strong correlation to whether the gene was detected as DMG or not (Supplementary Fig. 14). At last, the number of homeologous pairs both of which were detected as DMGs at S1 and S4 was statistically higher than expected, especially in CG and CHG contexts (Supplementary Table 7), implying unknown reciprocal effects after polyploidization in specific homeologous pairs. This finding contrasts with the independent evolution of homeologous nucleotide sequences found in the genomes of natural tetraploids of *A. kamchatica*^22^.

In contrast to the large change in synthetic polyploids across generations, both in DNA methylation and gene expression, the change in natural polyploids, as well as in diploid progenitors, was smaller (Supplementary Fig. 7 and Fig. 6e). This implies a loss of plasticity in allopolyploids depending on their evolutionary age. The natural tetraploid genome, in which the expression and methylation patterns experienced selection and adaptation^8^, can be considered as already stabilized, while newly formed polyploids still have dynamic epigenomes and transcriptomes^16,44,45^, potentially increasing the variation in phenotype^46^ on which selection can act.

A major part of both DMGs and DEGs are conserved between G1 and G4 in synthetics in both conditions, suggesting that the largest change occurred at the earliest generation after polyploidization. Given that 60% of DMGs were maintained in three generations from G1 to G4, a major part (85%) should have been conserved at each generation on average if we assume a constant rate. This result suggests that the impact of allopolyploidization on the epigenome is the strongest at the first generation, and relatively smaller at subsequent generations. In addition to this autonomous change, environmental conditions might affect the change through selective pressures, which would eventually result in variations among ecological populations.

Despite the possible effect of environments, the GO analysis of DMGs and DEGs did not show a clear accumulation of environment-specific genes related to the conditions. This might be due to the small number of generations or relatively mild selective pressure in a growth chamber, excluding environmental fluctuations and competition among individuals. Longer-term selective cultivation in wild conditions might accelerate the accumulation of change and lead to clearer environment-related effects on DNA methylation. In addition, targeting more tissue types may lead to a comprehensive understanding of the biological significance of DNA methylation change^43,47^. Most of all, further research on reproducibility should be done to assess whether a similar pattern is achieved in multiple independent lines and also whether the divergence between synthetics in two different conditions further increases in the subsequent generations. Thus, additional analyses in further generations with replication of lines and experiments in natural conditions will be useful to advance the discussion on the reproducibility of outcomes and the effect of environments on the evolution of DNA methylation patterns in neopolyploids.

In addition to the epigenetic and transcriptomic changes of the new synthetic polyploid, we have seen another interesting phenotype in reproduction. As found in other newly synthetized polyploids in *Capsella bursa-pastoris*^48^ and *A. suecica*^17^, our synthetic tetraploid autonomously turned to be self-fertile immediately after polyploidization. This should be attributed to the combination of *S*-loci between two progenitors: one selfing progenitor with a dominant but mutated *S*-locus (*A. lyrata*) and another outcrossing progenitor with a recessive *S*-locus (*A. halleri*), as revealed in natural polyploids of *Capsela*^48^, *A. suecica*^49^, and *A. kamchatica*^50^. These immediate shifts to selfing can contribute to the establishment of new polyploids as a new independent species.

This study offered the first step towards a more comprehensive view of the early stage after allopolyploid formation, combining transcriptomic and epigenomic analysis and highlighting the role of environmental stress. Future efforts in other species and in natural conditions, as well as considering additional epigenetic mechanisms, will shed more light on the complex, yet prevalent, successful formation of polyploids. Ultimately, bridging short- and long-term responses might reveal the reasons and mechanisms behind the universality of polyploidy in the evolutionary history of land plants.

## Methods

### Plant material and sequencing

The diploid progenitors and natural tetraploids were collected from natural localities and propagated by self-fertilization several times in the laboratory before the experiment. An artificial synthetic tetraploid line was constructed by crossing *A. halleri* subsp. *gemmifera* (maternal) and *A. lyrata* subsp. *petraea* (paternal), which were genetically the same lines as used in our previous studies^22,24,25^ and shown to be the closest to the progenitors of natural tetraploids. Among 4 individuals of the F1 diploid hybrid obtained by crossing, one individual spontaneously polyploidized to produce tetraploid offspring. One individual among them was employed as the mother of all individuals used to start the incubation as G1 in two conditions.

We incubated one synthetic tetraploid line (S), two natural lines of *A. kamchatica* originating from Alaska, U.S. (ALK) and Takashima, Japan (TKS), and the two progenitor species (P), *A. halleri* subsp. *gemmifera* and *A. lyrata* subsp. *petraea* (localities in Supplementary Table 8) in two conditions, summarized in Supplementary Fig. 1. For each condition and generation, four individuals were incubated and propagated via self-fertilization except *for A. halleri*. Since *A. halleri* is self-incompatible, daughter individuals were clonally propagated instead of sexual reproduction at each generation. After the plants bolted and started flowering, 2–3 rosette leaves per individual were collected (Supplementary Fig. 1). At the end, seeds were collected from each individual, and the seeds from successful mother plants were incubated for the next generation.

At each generation for each genotype, three individuals were selected for sequencing as biological replicates (Supplementary Fig. 1). The tissue was used to extract DNA and RNA for WGBS-seq and RNA-seq, respectively. Nucleic acid was extracted by the CTAB method^51^. The solution was split into two parts: one part was used for DNA extraction by NuceoSpin gDNA Clean-up (Macherey-Nagel) and another for RNA extraction by RNeasy (QIAGEN). DNA libraries for WGBS-seq were prepared using KAPA Hyper Prep Kit with TruSeq DNA Single Indexes Set (Illumina) after BS-treatment by EZ DNA Methylation-Gold Kit (ZYMO Research). RNA libraries were synthesized using the Illumina TruSeq stranded mRNA kit. All libraries were sequenced by Illumina NovaSeq 6000 in 150 bp paired-end mode. The basic information about the result of sequencing, mapping, and BS-conversion is summarized in Supplementary Data 1.

### *A. halleri* and *A. lyrata* genome sequencing

To obtain informative spatial information about differentially methylated regions, we sequenced the genomes of the *A. lyrata* and *A. halleri* subspecies used as progenitors of the synthetic tetraploid. The high-molecular-weight DNA was extracted from leaf tissue using Genomic-tip 20 (QIAGEN). DNA was sheared with Megaruptor 3 (Dagenode), and the libraries were prepared using SMRTbell Express Template Prep Kit 2.0. The libraries were size-selected with BluePippin (Sage Science) and sequenced on one SMRT cell 8 M per library on a PacBio Sequel II instrument in HiFi mode, with a movie collection time of 30 h, using Sequel II Sequencing Kit 2.0 (Pacific Biosciences).

HiFi reads were assembled with HiFiasm (v.0.14 r312)^52^ at default settings. For *A. lyrata*, haplotigs were removed with Purge Haplotigs (v1.1.1, subcommand cov with -l 30 -m 67 -h 140 -j 80)^53^. Organellar contigs were removed from the primary contigs by BLASTN alignment (BLAST+ v.2.11.0, -evalue 1e-2 -max_target_seqs 1)^54^. Chloroplast contigs were removed if the hit had 99% or more query coverage and 85% or more similarity. A contig was classified as a mitochondrion if the hit span >70% of the query length over at least 5 kb and had at least 98% similarity. After circularization and polishing, one contig representative of the chloroplast genome was kept for each assembly. The *A. halleri* mitochondrial genome was represented by 4 contigs, *A. lyrata* mitochondrial genome consisted of one contig. Organellar contigs were removed from the assembly prior to scaffolding. Pseudomolecules were built with RagTag (v1.1.1)^55^ with the scaffold option at default parameters. Unanchored and organellar contigs were added as separate individual sequences to the two sets of 8 chromosome-level pseudomolecules. To assess completeness, assemblies were scanned with BUSCO (v.5.2.2, genome mode, eudicots_odb10 dataset)^56^ and with KAT (v.2.4.1, subcommand comp, -m 21 -H 10000000000)^57^ using unassembled reads and the final pseudomolecules, which resulted in the completeness score of 99.64% (*halleri*) and 99.85% (*lyrata*).

Genes were annotated with MAKER (v.3.01.04, -fix_nucleotides)^58^ with different datasets. Proteomes were downloaded from *A. thaliana* TAIR10, *A. lyrata*^59^, and *A. halleri*^25^. RNA-Seq evidence from *A. halleri* and *A. lyrata* was downloaded from NCBI SRA (Supplementary Table 9). Adapters were removed with fastp (v.0.20.1 -z 4 -l 35 -w 10)^60^ and trimmed reads were aligned to the assemblies with HISAT2 (v.2.2.1, --dta --max-intronlen 100000)^61^. Alignments were parsed with SAMtools (v.1.13)^62^ and used as input for StringTie2 (v.2.1.7, -m 150)^63^. Gene models were converted to GFF format with gffread (v.0.12.7, -L)^64^.

Untranslated regions were added to the MAKER gene predictions with PASA (v.2.5.3)^65^ upon alignment of trinity-assembled (v.2.8.5)^66^ RNA-Seq data to the assembly. Gene models in TE coding regions were detected by BLASTP alignment to a TE gene library, and genes with a hit spanning more than 60% of their CDS length at 40% similarity or more, with a minimum alignment of 33 amino acids, were removed. Transfer RNAs were predicted with tRNAscan-SE (v.2.0.9)^67^ and non-coding RNAs were predicted with Infernal (v.1.1.4, subcommand cmscan -Z 10000 --cut_ga --rfam --nohmmonly --fmt 2 –oskip)^68^. TEs and repeats were annotated with RepeatMasker (http://www.repeatmasker.org, v.4.1.2-p1, -qq -norna -no_is -gff -cutoff 250 -gccalc -engine ncbi) with the repeat library^69^.

Synteny analysis was performed using GENESPACE (v.1.3.1)^70^ within the automated workflow snake-GENESPACE (v0.1.0-alpha, https://github.com/kenji-yt/snake-GENESPACE). In addition to our genome assemblies of *A. helleri* and *A. lyrata*, data analyses involved the genome sequences and assemblies of *A. thaliana* (TAIR10, https://data.jgi.doe.gov), *A. lyrata* MN47 (https://phytozome-next.jgi.doe.gov/info/Alyrata_v2_1), and *A. lyrata* NT1 (https://figshare.com/projects/Arabidopsis_lyrata_genome_assemblies/162343). The conversion of FASTA-formatted assemblies and GFF annotations into GENESPACE input format (primary isoform peptide sequence FASTA and BED annotation) was done automatically by snake-GENESPACE using AGAT (v1.4.0)^71^. GENESPACE depends on Orthofinder (v.2.5.5 in default mode)^72^ and MCScanX (downloaded from GitHub on March 12th 2025)^73^.

To define and plot HE regions and compute DMR density (Supplementary Table 3), we used ad-hoc scripts made in *R* v4.0.5^74^ with the packages tidyverse v1.3.1^75^ and *GenomicRanges*^76^. We first defined a set of HE regions together with normal regions. For this purpose, we overlapped the *A. halleri* and *A. lyrata* coverage values and selected regions where *A. halleri* coverage was greater than nine-fold, and *A. lyrata* coverage was less than two-fold (Supplementary Figs. 2 and 3). With these regions, we counted the amount of overlapping DMRs and computed the density by dividing this value by the length of the regions. Additionally, we computed the ratio between hyper- and hypo-methylated DMRs for normal and HE regions.

### Sample quality assessment and basic WGBS data analysis workflow with ARPEGGIO

For a reproducible and automated WGBS data analysis, we used ARPEGGIO v2.0.0^77^ for both diploid and tetraploid samples, but for the diploid datasets, the read classification step by EAGLE-RC was abbreviated, and the alignment by Bismark for the following steps. In short, after a quality check with FastQC v0.11.8^78^, reads were trimmed with TrimGalore v0.6.5^79^ 10 bp 3′ and 5 bp 5′ to remove adapter sequences. Alignment and deduplication, both with Bismark v0.22.3^80^, and read classification with EAGLE-RC v1.1.2^81^ were run using the *A. halleri* genome assembly v2.2^25^, the *A. lyrata* genome assembly v2.2^22^, and their corresponding annotation. An additional alignment to the *A. halleri* chloroplast assembly^82^ was executed for in silico BS conversion check, assuming the lack of DNA methylation in the chloroplast genome, to calculate conversion efficiency. All alignments were analyzed with QualiMap v2.2.2d^83^ to obtain coverage information. The quality reports from FastQC, TrimGalore, Bismark, and QualiMap were combined in a single document via MultiQC v1.8^84^. For the samples displaying a significant proportion of overrepresented sequences, adapter sequences, and/or duplication, standard trimming procedures combined with deduplication after alignment mitigated most of the initial issues, leading to a limited reduction in coverage. After applying these procedures to mitigate overrepresentation, all samples passed standard read-quality checks. The intermediate results of quality checks, trimming, in silico conversion checks, alignment, deduplication levels, coverage, bias, and confidence of methylation calls are all included in a sample quality report available in the same repository as codes.

After read classification, methylation information was extracted from reads with Bismark v0.22.3^80^. This information was used for two downstream analyses. First, the confidence of methylation calls was assessed, and then GML and methylation levels within and around gene bodies were computed. Second, we performed differential methylation analysis in R v3.6.2^74^ with dmrseq v1.6.0^85^ to obtain information about regions showing significant change in methylation. ARPEGGIO was run with Conda-only mode, using twelve CPU cores Intel(R) Xeon(R) CPU E5-4640 at 2.40 GHz.

### Global methylation level and average methylation levels around gene bodies

To estimate the GML, we used imputed cytosine calls from METHImpute v1.10.0^86^, which is known to output a whole genome methylation level consistent with the weighted methylation level^87^. In short, METHImpute applies a hidden Markov model to WGBS data to impute methylation levels for all cytosines in the genome, enabling robust analysis even with low-coverage datasets. All of the predictions are accompanied by a posterior probability estimating their confidence. High-quality calls were defined as calls with posterior probability >0.9. The proportion of high-quality cytosine calls was also included in the quality assessment of samples. The GML for each species was calculated with the average of the sum of all (imputed) methylated cytosines divided by the total number of cytosines. To exclude effects from HE regions, the imputation was done on masked input data where low-coverage regions were excluded. For each condition separately, the coverage of G4 synthetics was used to mask any 100 kb window with coverage less than two-fold from all samples.

In addition to GML, METHImpute was also used to compute the average methylation rate within gene bodies and in their 500 bp flanking regions for each methylation context: CG, CHG, and CHH. Gene bodies were defined as the regions between the transcription start site (TSS) and the transcription termination site. With 500 bp, flanking regions represented 13% of the *A. halleri* assembly, together with 27% from gene bodies, leaving 60% to intergenic regions. For *A. lyrata*, flanking regions represented 14% of the assembly, gene bodies 28%, and intergenic regions 58%.

### MDS analyses

To construct MDS plots, we used previous approaches^88–90^. In short, coverage data from *Birsmark* was first filtered to only take into account overlapping Cs across replicates and samples with a certain amount of coverage (≥ 3X). Next, methylation proportions were arcsin transformed to stabilize the variance (to prevent signal driven by the mean since a higher mean leads to higher variance). The whole dataset was split into two conditions, and for each condition, there were two separate progenitors’ sides (a total of four plots). Since MDS depends on the amount of Cs selected, we used several thresholds to assess if the relationship between samples would change significantly. For each threshold (10,000, 100,000, 1,000,000, and all), the “top” Cs with the maximum difference in transformed methylation proportion between samples were selected.

### Differential methylation and downstream analyses

Differential methylation was done separately for each methylation context (CG, CHG, and CHH) through ARPEGGIO. The output from each of these analyses provided a list of regions showing differential methylation (DMRs), together with their coordinates in the genome. The numbers and the length of DMRs are summarized in Supplementary Table 6. With these lists, ARPEGGIO ran downstream analyses to find and output all DMRs overlapping with annotated gene regions. ARPEGGIO was run with Conda-only mode, using twelve CPU cores Intel(R) Xeon(R) CPU E5-4640 at 2.40 GHz.

Additional downstream analyses were done for DMRs. First, we computed and visualized the number of hypo- and hypermethylated DMRs for each context and progenitor’s side (Fig. 3). Second, we checked the overlap between DMRs and genic, intergenic, or flanking gene regions (Fig. 5). The overlap was defined as at least one bp in common between a functional region and a DMR. If a DMR would overlap with several functional regions, the priority was set first to genic regions, then flanking regions, and last intergenic regions, meaning that a DMR overlapping with all three would be classified as overlapping with genic regions only. Genic regions were defined as starting from the transcription start site until the transcription end site. Also, genic regions with at least one overlapping DMR were defined as DMR-associated genes (DMGs).

All of these analyses were performed with R v4.0.2^74^. To import large files, we used data.table v1.13.0. For data wrangling, barplots, and donut charts, we used tidyverse v1.3.0^75^ and gridExtra v2.3^91^. For all analyses involving overlaps between regions, we used GenomicRanges v1.40.0^76^.

### Gene overrepresentation analyses

A list of differentially expressed and methylated genes (DEMGs) was used for gene overrepresentation tests with TopGo v2.52.0^92^. The gene universe set was defined on expressed genes for H- and L-side (see “Differential expression analysis” section), setting elim and fisher for the algorithm and statistic parameters to reduce the propagation of false positives within the GO directed acyclic graph, and the *p*-value was calculated using this algorithm. Significance threshold was set to *p* < 0.05. To assign *A. thaliana* genes comparable to *A. halleri* and *A. lyrata* genes, we checked for orthologous genes based on BLAST reciprocal best hit between genes of our reference (*A. halleri* or *A. lyrata*) and *A. thaliana* (TAIR10), and 20,418 (H-side) and 20,435 (L-side) genes were considered for GO analysis in each subgenome, respectively (Table 1). The overrepresentation test (OT) was performed for DEMGs for each condition, progenitors’ side, and generation, for a total of 8 tests (Fig. 5a).

### RNA-seq workflow

To analyze RNA-seq data, reads were first quality checked with FastQC v0.11.8^78^, and all quality reports were merged with MultiQC v1.8^84^. Next, reads were aligned with STAR v2.7.3a^93^ to our genome assemblies of *A. halleri* and *A. lyrata*. For synthetic and natural *A. kamchatica* data, reads were mapped to both genome assemblies and classified to progenitors’ sugenomes (with EAGLE-RC v1.1.2^81^ genotype information) and --paired (for paired-end reads). As a final step, counts were obtained with featureCounts from Subread v2.0.1^94^.

### Differential expression analysis

For differential expression (DE) analyses, we used edgeR v3.32.1^95^. All samples were analyzed together, and reads were filtered with default parameters, resulting in 19,006 genes passing filters for *A. halleri* and H-side genes (out of 32,527) and 17,939 genes passing filters for *A. lyrata* and L-side genes (out of 28,737). Library sizes were scaled, and the relationship between samples was investigated through the biological coefficient of variation (BCV) (Fig. 6a). DE was computed for all pairwise comparisons planned for the experiment (Fig. 1a) and between generations from the same line. Gene-wise exact tests were used to compute DE, and *p*-values were adjusted with the false discovery rate to control for multiple testing. For all pairwise comparisons, a volcano plot or bar plot was used to visualize DEGs (Fig. 6 and Supplementary Fig. 10). As for BS-seq data, we removed all DE genes falling in low-coverage regions to exclude signal from HE events. We did this for all pairwise comparisons involving synthetic polyploids. Chromosome plots were done with karyoploteR v1.16.0^96^.

### Heatmaps with expression data

To visualize expression profiles for all samples and their relationship, we generated a heatmap for the expression of the most variable genes for each progenitor’s side and condition (Fig. 7). To do this, we imported raw counts for all samples, and we filtered out genes falling in low coverage regions defined beforehand with BS-seq data (coverage less than 2-fold). Next, we applied a variance stabilizing transformation to have the expression variance approximately independent from the mean expression^97^. As a final step, the expression variance of all genes across all samples was computed, and the 1000 genes with the highest variance were selected to generate a heatmap and cluster the expression profiles of all the samples.

### Combining transcription and methylation data

To compare methylation and expression changes, we first computed raw methylation changes for all DMRs found between synthetic *A. kamchatica* and progenitors (PvsS1 and PvsS4). We created a command-line R script requiring as arguments (1) the RData file resulting from a given comparison from ARPEGGIO, (2) the output name, (3) the set of Bismark coverage files from a given progenitor, and (4) the coverage files for a given synthetic. This script was executed for all conditions, comparisons, progenitors’ sides, and contexts. With the raw methylation values, we assigned significant gene expression changes (log fold-change values) to genes showing differential methylation (if significant expression changes occurred). Next, we plotted the association between raw methylation change and logFC for all contexts, comparisons, and subgenomes (Supplementary Fig. 12). For each plot, we computed a linear regression to assess the relationship between methylation and expression and also tested for correlation between the two with Pearson’s product moment correlation coefficient (cor.test function with Rv4.0.2)^74^.

To visualize the overlap between DEGs and DMGs, we plotted Venn diagrams and column graphs for PvsS1 and PvsS4 comparisons for both progenitors’ sides and conditions (Supplementary Fig. 8). For these diagrams, differentially methylated genes were defined as genes showing differential methylation in at least one context. Data wrangling was performed with tidyverse v1.3.0^75^, and VennDiagram v1.7.0 was used to plot diagrams.

### Reporting summary

Further information on research design is available in the Nature Portfolio Reporting Summary linked to this article.

## Supplementary information

Supplementary Information

Peer Review File

Description of Additional Supplementary Files

Supplementary Data 1

Reporting Summary

## Source data

Source Data

## Data Availability

The genome assemblies are registered in NCBI: *A. halleri* genome as JAJUOU000000000 under BioProject PRJNA788966 and *A. lyrata* genome as JAJUOV000000000 under BioProject PRJNA788965. BS-DNA sequencing datasets, DNA sequencing datasets, and RNA sequencing datasets are accessible at DDBJ under accessions PRJDB12567, PRJDB39671, and PRJDB12582, respectively. Source data are provided with this paper.

## Code availability

All code used in this paper is available at Zenodo [10.5281/zenodo.20304945].
