# Supplementary material for: Convergence and divergence of DNA methylation and gene expression patterns in neopolyploid Arabidopsis kamchatica
Source: Nat Commun. 2026 Jun 30;17:8419. doi: 10.1038/s41467-026-75042-4 (PMC13478183; doi:10.1038/s41467-026-75042-4)
Supplement: Supplementary file 1 [file 41467_2026_75042_MOESM1_ESM.pdf]

Supplementary Table 1 | The statistical evaluation of survival and fertility levels of the used lines between mild and stress conditions. The number of total, survived, and fertile (i.e., producing seeds) individuals in each genotype. *p*-values by the Fisher's exact test (two-sided) between the number of individuals survived/non-survived and fertile/others between two incubating conditions are shown in each genotype. Significant results ($p < 0.05$) are highlighted in bold. The fertility of *A. halleri* was not considered, as it is self-incompatible and propagated clonally.

		sum of G1 to G4/G5				
		total	survived	fertile	survival	fertility
genotype	condition	number of indivicuals			*p*	*p*
halleri	mild	16	16	n.a.	1	n.a.
	stress	16	16	n.a.		
lyrata	mild	16	16	13	1	1
	stress	16	16	14		
ALK	mild	17	17	17	1	**0.0445**
	stress	16	16	12		
TKS	mild	22	22	22	1	1
	stress	20	20	20		
synthetic	mild	24	24	18	0.0567	**0.0245**
	stress	16	13	6		

Supplementary Table 2 | Expected genome sizes with and without predicted chromosome losses. Expected chromosome losses were computed by measuring the length of all regions with less than 2x coverage of BS DNA-seq read-mapping. The expected genome size represents the theoretical sum of two progenitors' genomes. The actual genome sizes of the individuals of later generations of synthetics (at G6 for mild condition and at G7 for stress condition) were measured by flow cytometry (Cyflow Space, Sysmex).

	A. kamchatica synthetics (G4)			
Condition	Mild		Stress	
Expected genome size	475 Mbp			
Sub-genome	H	L	H	L
Expected chromosome loss	26 Mb	63 Mb	26 Mb	51 Mb
Expected genome size with chromosome losses	386 Mbp		398 Mbp	
Genome size measured by flow cytometry	479 Mbp (G6)		473 Mbp (G7)	

Supplementary Table 3 | DMR density in homeologous exchanged (HE) regions and representative normal regions. The number of DMRs and the density of DMRs per Mbp between G1 progenitors and the G4 synthetics were calculated in the HE region and the non-HE region, as indicated in Fig. S2 in pink and green, respectively. The representative non-HE regions were arbitrarily chosen to match the length of the HE regions (Mbp).

Condition	Mild		Stress	
Region	HE	non-HE	HE	non-HE
Length	24.8Mb	22Mb	10.6Mb	17.5Mb
DMRs number	1’430	1’570	1’095	2’501
DMRs density (per Mb)	57.66	71.36	103.30	142.91
Hyper DMRs number (density per Mb)	404 (16.29)	298 (13.54)	123 (11.60)	169 (9.66)
Hypo DMRs number (density per Mb)	1026 (41.37)	1272 (57.82)	972 (91.70)	2332 (133.26)

Supplementary Table 4 | Overview of overlapping DMRs found in G1 and G4 synthetics against G1 progenitors.

Mild condition

	CG context		CHG context		CHH context	
	H	L	H	L	H	L
**DMRs overlap between PvsS1 & PvsS4**	**1’352**	**2’025**	**1’413**	**2’047**	**471**	**1’627**
Total DMRs in PvsS1	1’962	2’724	2’439	3’567	1’282	2’592
Overlap % within G1	68.9 %	74.3 %	57.9 %	57.4 %	36.7 %	62.8 %
Total DMRs in PvsS4	4’388	4’734	4’533	5’230	4’684	8’821
Overlap % within G4	30.8 %	42.8 %	31.2 %	39.1 %	10.1 %	18.4 %

Stress condition

	CG context		CHG context		CHH context	
	H	L	H	L	H	L
**DMRs overlap between PvsS1 & PvsS4**	**1’544**	**2’150**	**3’757**	**2’108**	**1’797**	**1’429**
Total DMRs in PvsS1	2’518	3’095	8’057	3’435	2’775	5’044
Overlap % within G1	61.3 %	69.5 %	46.6 %	61.4 %	64.8 %	28.3 %
Total DMRs in PvsS4	3’826	4’077	11’550	7’630	10’707	3’279
Overlap % within G4	40.4 %	52.7 %	32.5 %	27.6 %	16.8 %	43.6 %

Supplementary Table 5 | Summary of the GO terms related to ion-transport and ion-homeostasis overrepresented in the DMGs in G1 and G4 synthetics against G1 progenitors. The GO categories detected in each context are marked as y.

	pair	syn G1 vs. pro G1 Mild			syn G4 vs. pro G1 Mild			syn G1 vs. pro G1 Stress			syn G4 vs. pro G1 Stress			syn G1 vs. pro G1 Mild			syn G4 vs. pro G1 Mild			syn G1 vs. pro G1 Stress			syn G4 vs. pro G1 Stress		
	subgenome	halleri side												lyrata side											
	context	CG	CHG	CHH	CG	CHG	CHH	CG	CHG	CHH	CG	CHG	CHH	CG	CHG	CHH	CG	CHG	CHH	CG	CHG	CHH	CG	CHG	CHH
GO:0000041	transition metal ion transport													y						y			y		
GO:0030001	metal ion transport						y						y	y						y			y		
GO:0010959	regulation of metal ion transport																			y					
GO:0006826	iron ion transport																y								
GO:0006816	calcium ion transport						y																		
GO:0015691	cadmium ion transport																			y					
GO:0006813	potassium ion transport																			y			y		
GO:0006814	sodium ion transport																								y
GO:0006817	phosphate ion transport																				y				
GO:0006811	monoatomic ion transport									y			y												
GO:0015698	inorganic anion transport																		y						
GO:0070574	cadmium ion transmembrane transport																y			y					
GO:0034755	iron ion transmembrane transport																y								
GO:0071421	manganese ion transmembrane transport																y								
GO:0070588	calcium ion transmembrane transport						y				y														
GO:0035435	phosphate ion transmembrane transport																	y			y				
GO:0071805	potassium ion transmembrane transport																			y		y	y		y
GO:0098655	monoatomic cation transmembrane transport						y		y											y					
GO:0098660	inorganic ion transmembrane transport						y						y							y	y		y		
GO:0098661	inorganic anion transmembrane transport						y						y					y	y	y	y		y		
GO:0034220	monoatomic ion transmembrane transport						y		y	y			y							y					
GO:0043266	regulation of potassium ion transport																			y		y	y		
GO:0044070	regulation of monoatomic anion transport	y					y	y	y		y														
GO:0034765	regulation of monoatomic ion transmembrane transport																			y		y			
GO:0032412	regulation of monoatomic ion transmembrane transporter activity	y					y	y	y		y									y					
GO:0006873	intracellular monoatomic ion homeostasis											y													
GO:0006879	intracellular iron ion homeostasis											y													
GO:0006882	intracellular zinc ion homeostasis				y				y			y													
GO:0030003	intracellular monoatomic cation homeostasis											y													
GO:0050801	monoatomic ion homeostasis																y						y		
GO:0055062	phosphate ion homeostasis		y		y	y			y			y													
GO:0055071	manganese ion homeostasis								y																
GO:0055072	iron ion homeostasis											y													
GO:0055080	monoatomic cation homeostasis											y					y								

Supplementary Table 6 | General statistics on DMRs found in the comparisons between progenitors and first (PvsS1) and fourth (PvsS4) generation synthetics. For each condition, subgenome, context and direction of methylation (hyper = increase of methylation in the synthetic, hypo = decrease of methylation in the synthetic), several statistics were computed: total number of DMRs (total), average size and standard deviation (avg and sd), median size (median), maximum size of DMR lengths (max bp), the total length of all DMRs (length), and the total number of cytosines covered in all DMRs (totC).

			Cold conditions							Hot conditions						
	side	context	type	total	avg	sd	median	max bp	length	totC	total	avg	sd	median	max bp	length	totC
PvsS1	halleri	CG	all	1962	502bp	383bp	432bp	5.2kbp	1Mb	40305	2518	494bp	362bp	441bp	5kbp	1.2Mb	62203
			hyper	1128	586bp	432bp	504bp	5.2kbp	0.7Mb	25043	1471	549bp	403bp	475bp	5kbp	0.8Mb	39230
			hypo	834	389bp	266bp	348bp	1.9kbp	0.3Mb	15262	1047	416bp	276bp	388bp	2.1kbp	0.4Mb	22973
		CHG	all	2439	590bp	564bp	485bp	10.9kbp	1.4Mb	57087	8057	885bp	771bp	688bp	16.8kbp	7.1Mb	353148
			hyper	985	646bp	692bp	510bp	10.9kbp	0.6Mb	22969	681	602bp	729bp	476bp	10.7kbp	0.4Mb	19426
			hypo	1454	552bp	455bp	468bp	7.6kbp	0.8Mb	34118	7376	911bp	770bp	709bp	16.8kbp	6.7Mb	333722
		CHH	all	1282	493bp	358bp	462bp	7.6kbp	0.6Mb	123843	2775	525bp	535bp	467bp	12.6kbp	1.5Mb	309434
			hyper	875	510bp	308bp	480bp	5.1kbp	0.4Mb	87365	437	505bp	290bp	485bp	2.6kbp	0.2Mb	50874
			hypo	407	456bp	446bp	409bp	7.6kbp	0.2Mb	36478	2338	528bp	569bp	464bp	12.6kbp	1.2Mb	258560
	lyrata	CG	all	2724	573bp	544bp	469bp	9.4kbp	1.6Mb	65729	3095	575bp	519bp	472bp	8.1kbp	1.8Mb	87321
			hyper	1874	640bp	606bp	514bp	9.4kbp	1.2Mb	49462	2207	615bp	564bp	499bp	8.1kbp	1.4Mb	67512
			hypo	850	425bp	329bp	370bp	3.8kbp	0.4Mb	16267	888	474bp	365bp	419bp	3.3kbp	0.4Mb	19809
		CHG	all	3567	685bp	752bp	501bp	9.7kbp	2.4Mb	92556	3435	737bp	690bp	572bp	8.9kbp	2.5Mb	121076
			hyper	2449	795bp	836bp	563bp	9.7kbp	1.9Mb	71767	2663	795bp	713bp	608bp	8.2kbp	2.1Mb	102509
			hypo	1118	443bp	437bp	386bp	9.2kbp	0.5Mb	20789	772	534bp	560bp	442bp	8.9kbp	0.4Mb	18567
		CHH	all	2592	487bp	331bp	456bp	6.4kbp	1.2Mb	268023	5044	555bp	382bp	507bp	10kbp	2.8Mb	600172
			hyper	822	525bp	457bp	460bp	6.4kbp	0.4Mb	88216	4242	571bp	404bp	518bp	10kbp	2.4Mb	514475
			hypo	1770	469bp	249bp	456bp	3.3kbp	0.8Mb	179807	802	467bp	218bp	468bp	1.6kbp	0.4Mb	85697
PvsS4	halleri	CG	all	4388	453bp	330bp	403bp	5.5kbp	2Mb	83483	3826	478bp	376bp	421bp	8.7kbp	1.9Mb	85292
			hyper	1898	511bp	364bp	450bp	5.5kbp	1Mb	38080	1434	526bp	381bp	455bp	5.3kbp	0.8Mb	32803
			hypo	2490	409bp	295bp	368bp	4.2kbp	1Mb	45403	2392	449bp	370bp	402bp	8.7kbp	1.1Mb	52489
		CHG	all	4533	668bp	528bp	561bp	10.1kbp	3.1Mb	116542	11550	723bp	575bp	604bp	16kbp	8.4Mb	325902
			hyper	743	617bp	646bp	501bp	10.1kbp	0.5Mb	17498	630	620bp	678bp	501bp	10.3kbp	0.4Mb	16859
			hypo	3790	678bp	501bp	574bp	7.9kbp	2.6Mb	99044	10920	729bp	568bp	611bp	16kbp	8Mb	309043
		CHH	all	4684	580bp	387bp	531bp	8.3kbp	2.7Mb	515615	10707	627bp	437bp	557bp	14.7kbp	6.7Mb	1270754
			hyper	247	462bp	356bp	442bp	3.3kbp	0.1Mb	22061	250	458bp	301bp	428bp	2kbp	0.1Mb	26032
			hypo	4437	587bp	387bp	535bp	8.3kbp	2.6Mb	493554	10457	631bp	439bp	560bp	14.7kbp	6.6Mb	1244722
	lyrata	CG	all	4734	520bp	472bp	434bp	9kbp	2.5Mb	102018	4077	556bp	520bp	460bp	10.8kbp	2.3Mb	97682
			hyper	2610	594bp	557bp	484bp	9kbp	1.6Mb	63563	2420	617bp	613bp	493bp	10.8kbp	1.5Mb	63776
			hypo	2124	430bp	316bp	388bp	3.6kbp	0.9Mb	38455	1657	467bp	322bp	420bp	4kbp	0.8Mb	33906
		CHG	all	5230	748bp	926bp	510bp	12.3kbp	3.9Mb	129660	7630	972bp	940bp	685bp	16.8kbp	7.4Mb	321922
			hyper	2027	1050bp	1323bp	598bp	12.3kbp	2.1Mb	61479	5449	1135bp	1033bp	806bp	16.8kbp	6.2Mb	272602
			hypo	3203	557bp	444bp	475bp	6.7kbp	1.8Mb	68181	2181	564bp	441bp	481bp	5.5kbp	1.2Mb	49320
		CHH	all	8821	613bp	436bp	552bp	9kbp	5.4Mb	1107335	3279	490bp	447bp	448bp	8.9kbp	1.6Mb	328362
			hyper	563	464bp	486bp	391bp	8kbp	0.3Mb	49408	1229	485bp	594bp	398bp	8.9kbp	0.6Mb	124844
			hypo	8258	623bp	431bp	562bp	9kbp	5.1Mb	1057927	2050	494bp	328bp	470bp	5.8kbp	1Mb	203518

Supplementary Table 7 | Homologous DMG pairs on PvS1 and PvS4. The number of DMGs on each H- or L-side, and the number of homeologous pairs among them are summarized. Statistical significance was determined using a two-group Chi-square test and the result was shown in the *p*-value (***: $p<0.001$, **: $p<0.01$, *: $p<0.05$).

Condition	Comparison	Context	DMGs on H-side	DMGs on L-side	Homeologous pairs	*p*-value	
						category	value
Mild	PvsS1	CG	338	350	17	***	7.9064E-07
		CHG	295	278	22	***	6.7655E-21
		CHH	121	222	3		0.11842041
	PvsS4	CG	770	765	62	***	1.226E-11
		CHG	385	432	23	***	2.9547E-08
		CHH	259	536	12	*	0.02857969
Stress	PvsS1	CG	506	448	18	*	0.02100057
		CHG	516	270	23	***	5.4511E-11
		CHH	189	266	9	***	1.2461E-05
	PvsS4	CG	624	557	39	***	7.613E-09
		CHG	748	406	45	***	5.7386E-17
		CHH	443	268	15	***	4.878E-05

Supplementary Table 8 | Locality information of the used genotypes.

Species	Subspecies		Locarity	Latitude		Longitude	
Arabidopsis halleri	*gemmifera*	H	Inagawa, Hyogo, Japan	N	34.89	E	135.35
Arabidopsis lyrata	*petraea*	L	Coryma river, Yakut, Russia	N	68.8	E	160.3
Arabidopsis kamchatica	*kamchatica*	ALK	Darling Creek, Alaska, USA	N	63.42	W	145.84
Arabidopsis kamchatica	*kawasakiana*	TKS	Takashima, Shiga, Japan	N	35.45	E	136.06

Supplementary Table 9 | The list of RNA-Seq datasets of *A. halleri* and *A. lyrata* used for the gene annotation.

A. halleri	*A. lyrata*
SRR5488374	SRR3020591
SRR5488375	SRR3020592
SRR5488376	SRR3020593
SRR5488377	SRR3020594
SRR5488378	SRR3020595
SRR5488379	SRR3020596
SRR5488380	SRR3020597
SRR5488381	SRR3993766
SRR5488382	SRR3993768
SRR5488383	SRR6053250
SRR5488384	SRR6053251
SRR5488385	SRR6053252
DRR054435	DRR054447
DRR054436	DRR054448
DRR054437	DRR054449
DRR054438	DRR054450

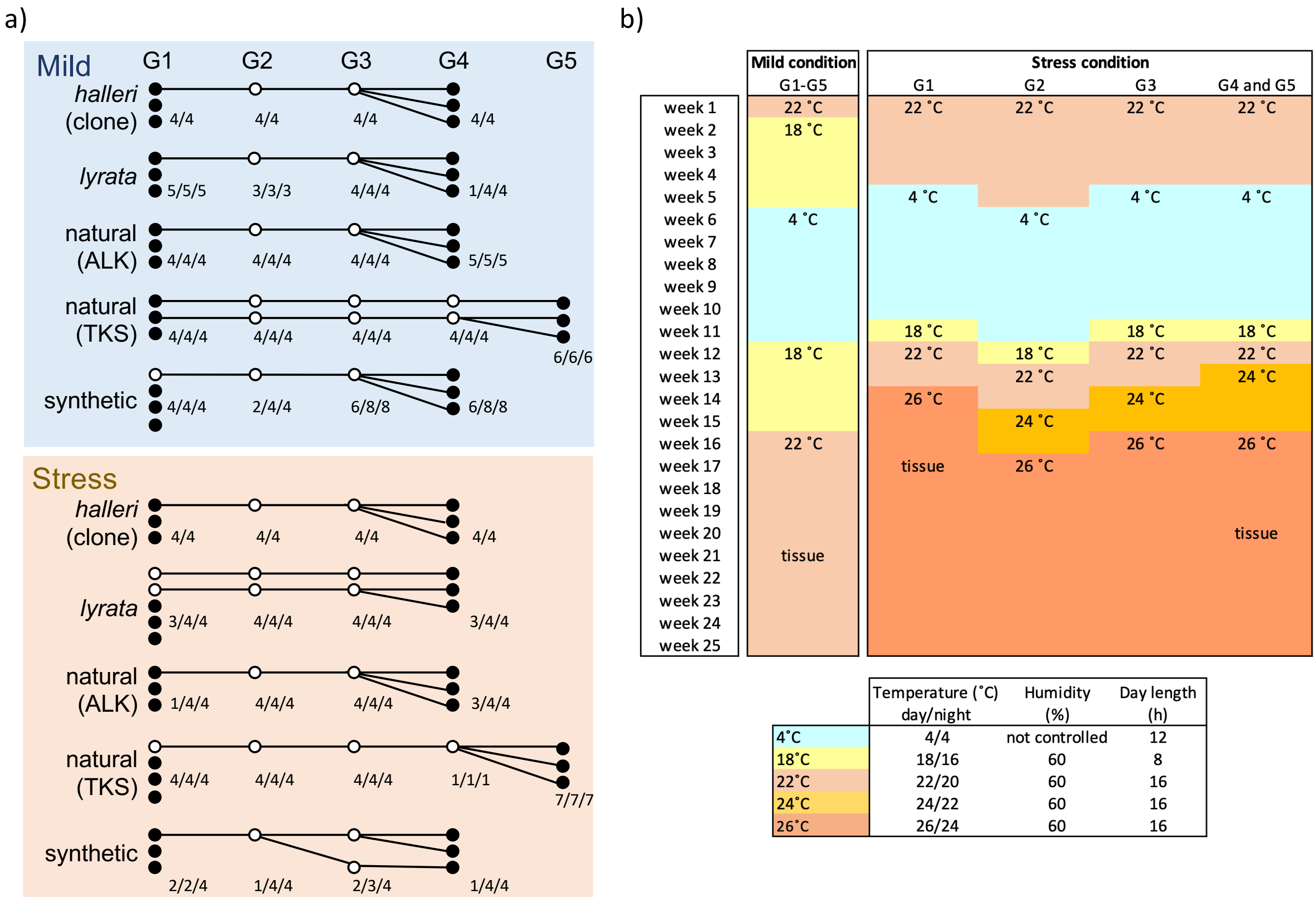

Supplementary Figure 1 | The pedigree of analyzed individuals and the details of two experimental growth conditions. a, Pedigree lineage for all lines included in this study grown in two conditions. The closed circles represent the sequenced and analyzed individuals. The vertical line indicates the mother (left) and the offspring (right). The numbers noted on each generation of each genotype denote the numbers of (reproductively successful individuals) / (individuals survived until the reproductive stage) / (incubated individuals). The G1 individuals of each line used for the two conditions were the siblings. **b**, Experimental growth conditions for each generation and condition. Each color indicates a specific combination of day/night temperatures, humidity, and day length. Each row from the top table indicates a specific week from the start of incubation. The timing of tissue sampling is suggested as 'tissue'.

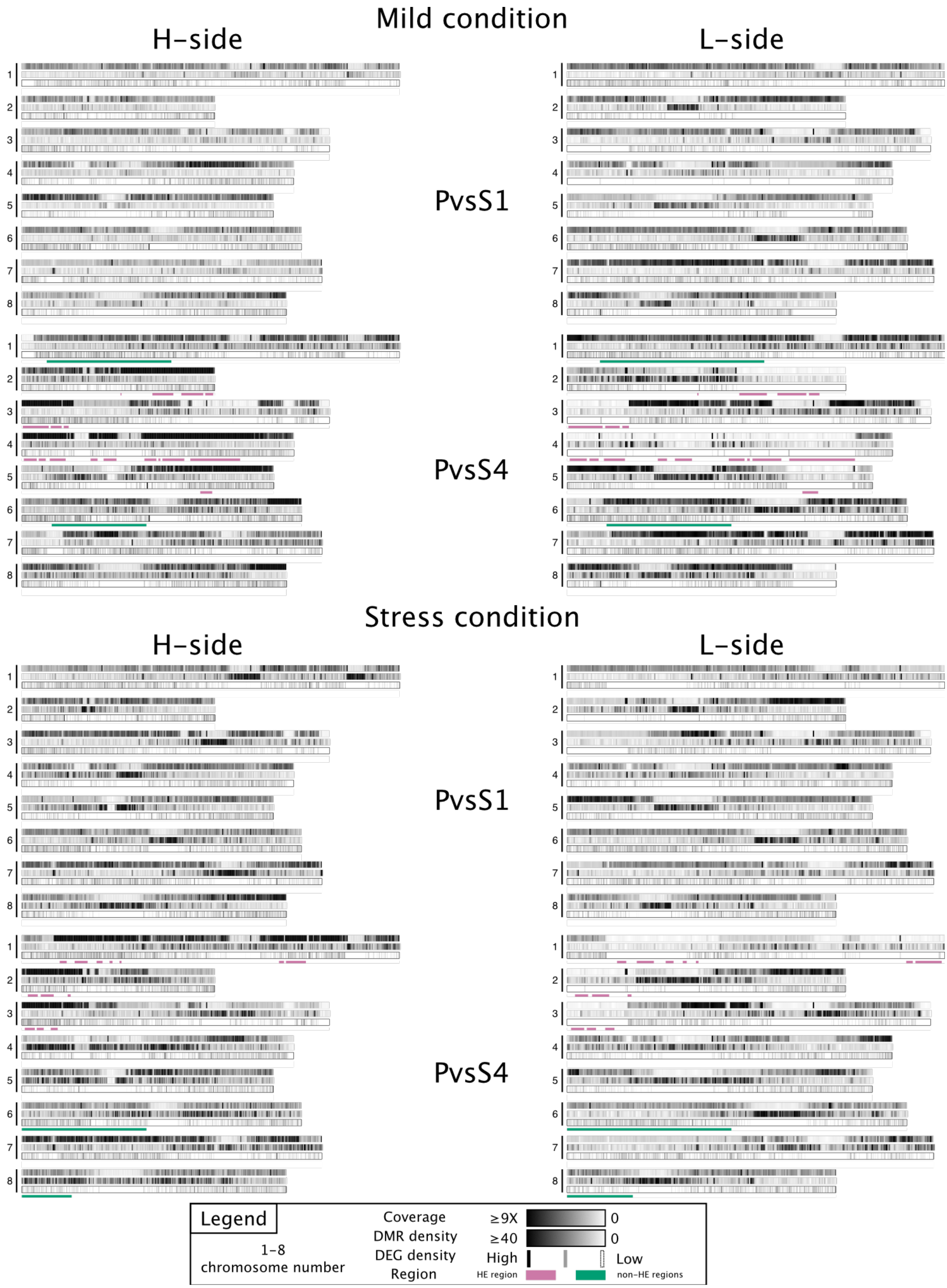

Mild condition
H-side
L-side
PvsS1
PvsS4
Stress condition
H-side
L-side
PvsS1
PvsS4
1
2
3
4
5
6
7
8
Legend
1-8
chromosome number
Coverage
DMR density
DEG density
Region
≥9X
0
≥40
0
High
Low
HE region
non-HE regions

Supplementary Figure 2 | Distribution of sequencing read coverage, DMG, and DEG along chromosomes for PvsS1 and PvsS4 of BS DNA-seq. Top panels are for mild condition and bottoms for stress condition. The left and right panels are for H-side and L-side, respectively. Each panel shows two comparisons, PvsS1 (progenitors vs. synthetic G1) and PvsS4 (progenitors vs. synthetic G4), and in each comparison 8 chromosomes are indicated. Three rectangles are allocated in each chromosome: top rectangle represents BS DNA-seq coverage (Low: 0X to High: 9X or more) of S1 or S4 samples, and the middle and bottom rectangles show relative DMG density and DEG density, respectively. The colored bars located below the rectangles indicate the regions considered as HE (pink) or non-HE (green) for the DMR density analyses in Supplementary Table2 (only in G4). To prevent the detection of false positive DMGs, the low coverage regions marked here as potential HE regions by pink (<2x by BS DNA-seq) were filtered out in the downstream analysis.

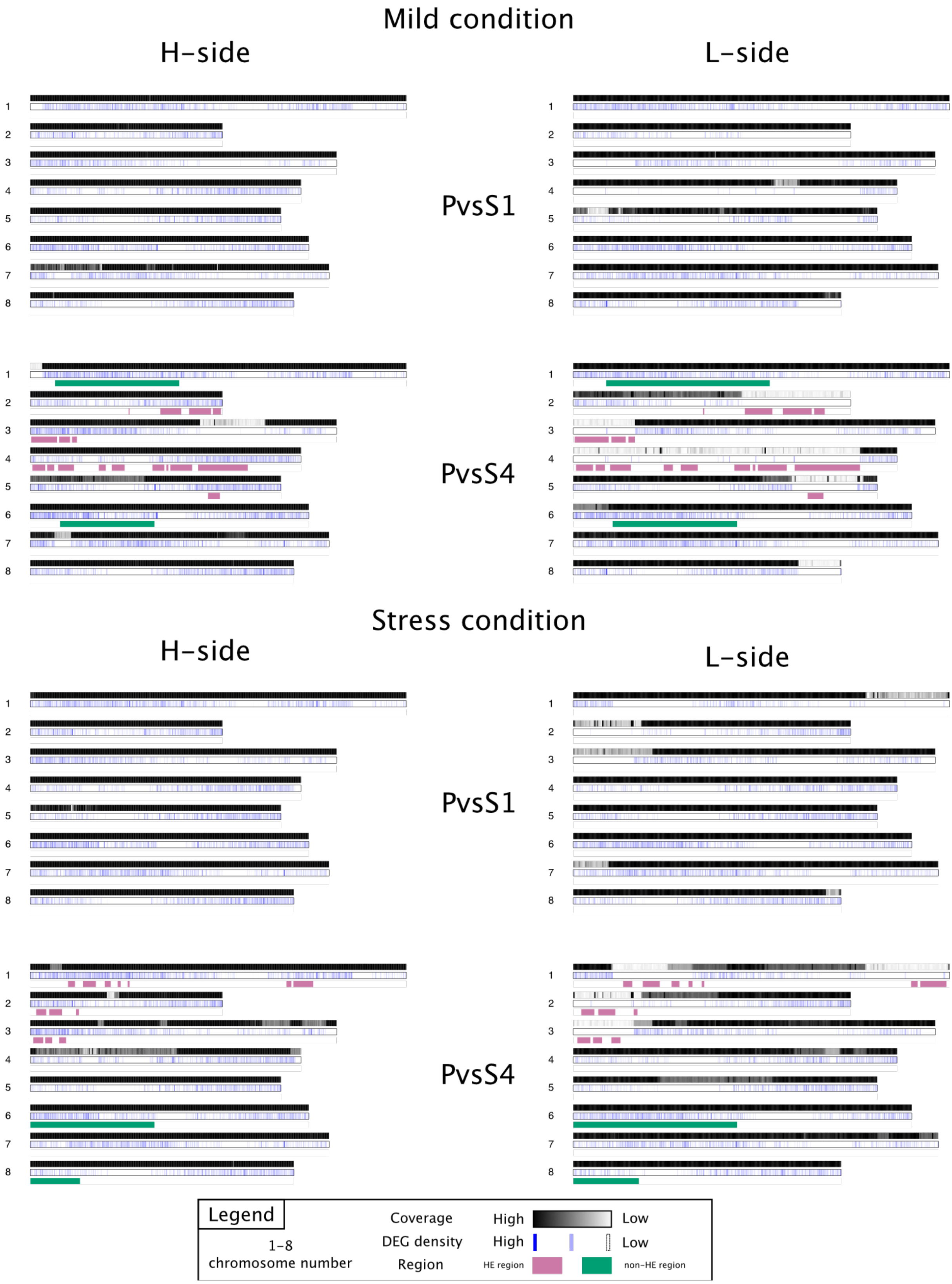
Mild condition
H−side
L−side
PvsS1
PvsS4
Stress condition
H−side
L−side
PvsS1
PvsS4
Legend
1−8
chromosome number
Coverage
High
Low
DEG density
High
Low
Region
HE region
non−HE region

Supplementary Figure 3 | Distribution of sequencing read coverage, DMG, and DEG along chromosomes for PvsS1 and PvsS4 of non-BS DNA-seq. The order of panels is the same as Supplementary Figure 2. Each panel shows two comparisons, PvsS1 (progenitors vs. synthetic G1) and PvsS4 (progenitors vs. synthetic G4), and in each comparison 8 chromosomes are indicated. Two rectangles are allocated in each chromosome: top rectangle represents DNA-seq coverage (Low: 0X to High: 40X or more), and the middle rectangles show relative DEG density with one line representing one DEG. The colored bars located below the rectangles indicate the regions considered as HE (pink) or non-HE (green) detected in Supplementary Figure 2.

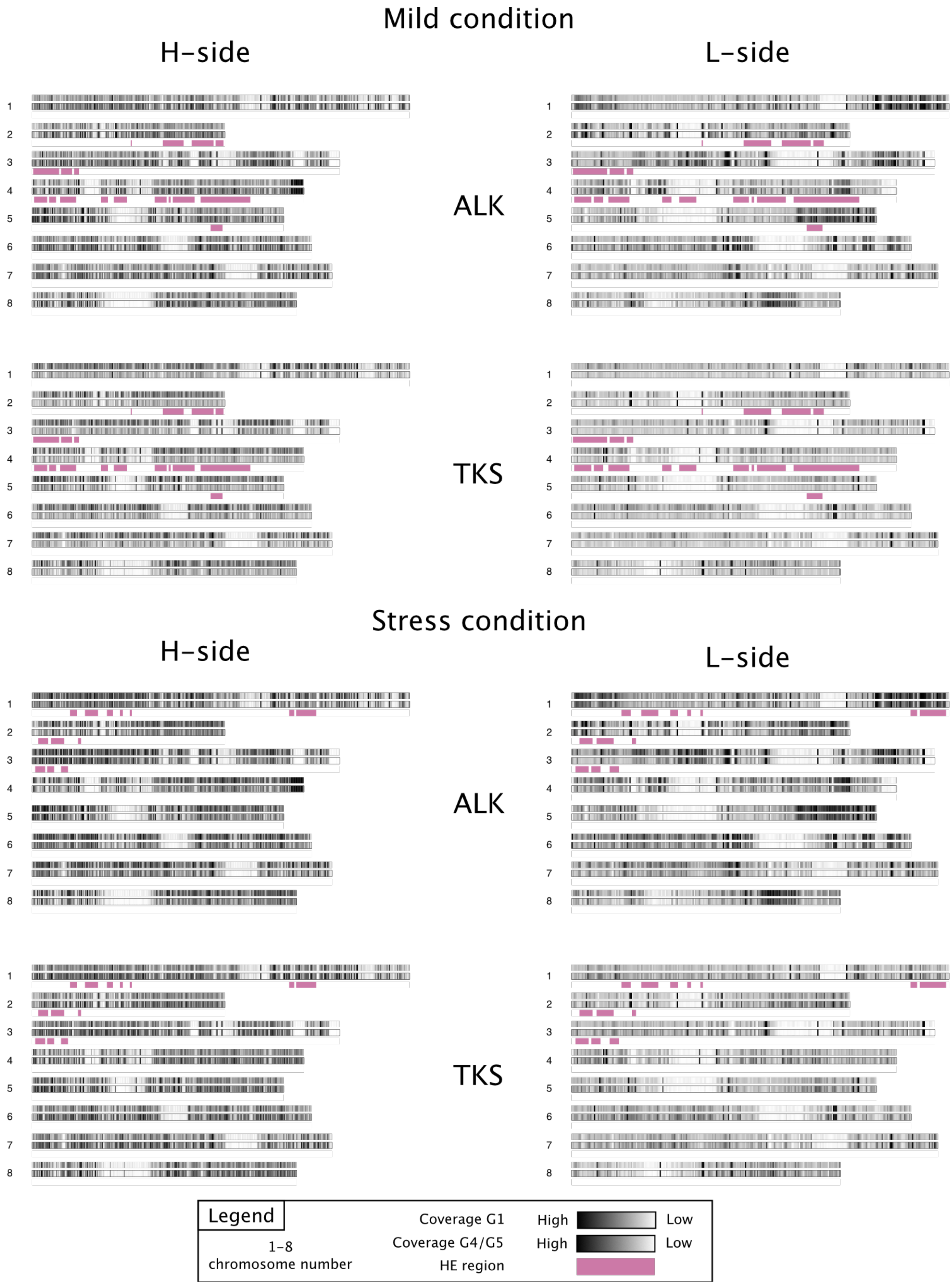

Supplementary Figure 4 | Distribution of BS DNA-sequencing read coverage along chromosomes for ALK and TKS in two conditions and at G1 (upper

rectangle) and G4/G5 (lower rectangle). Top panels are for mild condition and bottoms for stress condition. The right and left sides are for H-side and L-side, respectively. Each panel shows the mapping coverages of two natural tetraploids (ALK and TKS) in 8 chromosomes. Two rectangles are allocated to each chromosome, representing represent BS DNA-seq coverage at G1 (top) and G4 (ALK) /G5 (TKS) (bottom) (Low: 0X to High: 9X or more). The colored bars located below the rectangles indicate the regions considered as HE (pink) detected in synthetic tetraploid samples in Fig.S2a.

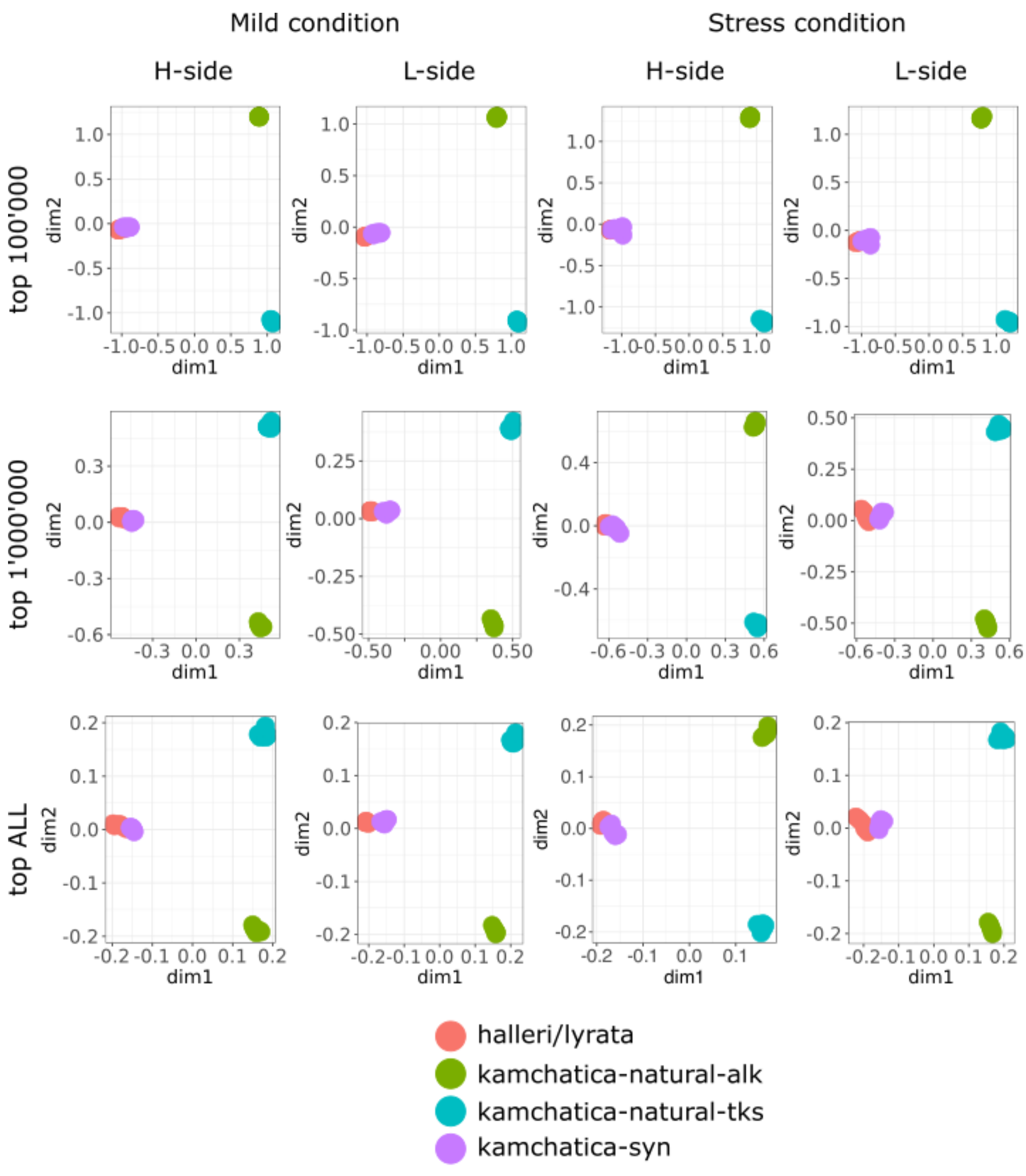

Supplementary Figure 5 | MDS plots of methylation pattern of cytosines by three different thresholds in two conditions and in two subgenomes. Three thresholds were selected to assess the relationship across samples: top (most variable) 100’000, 1’000’000, and all cytosines (mild condition: 10’589’212 H-side and 6’360’582 L-side, stress condition: 15’187’216 H-side and 8’046’230 L-side).

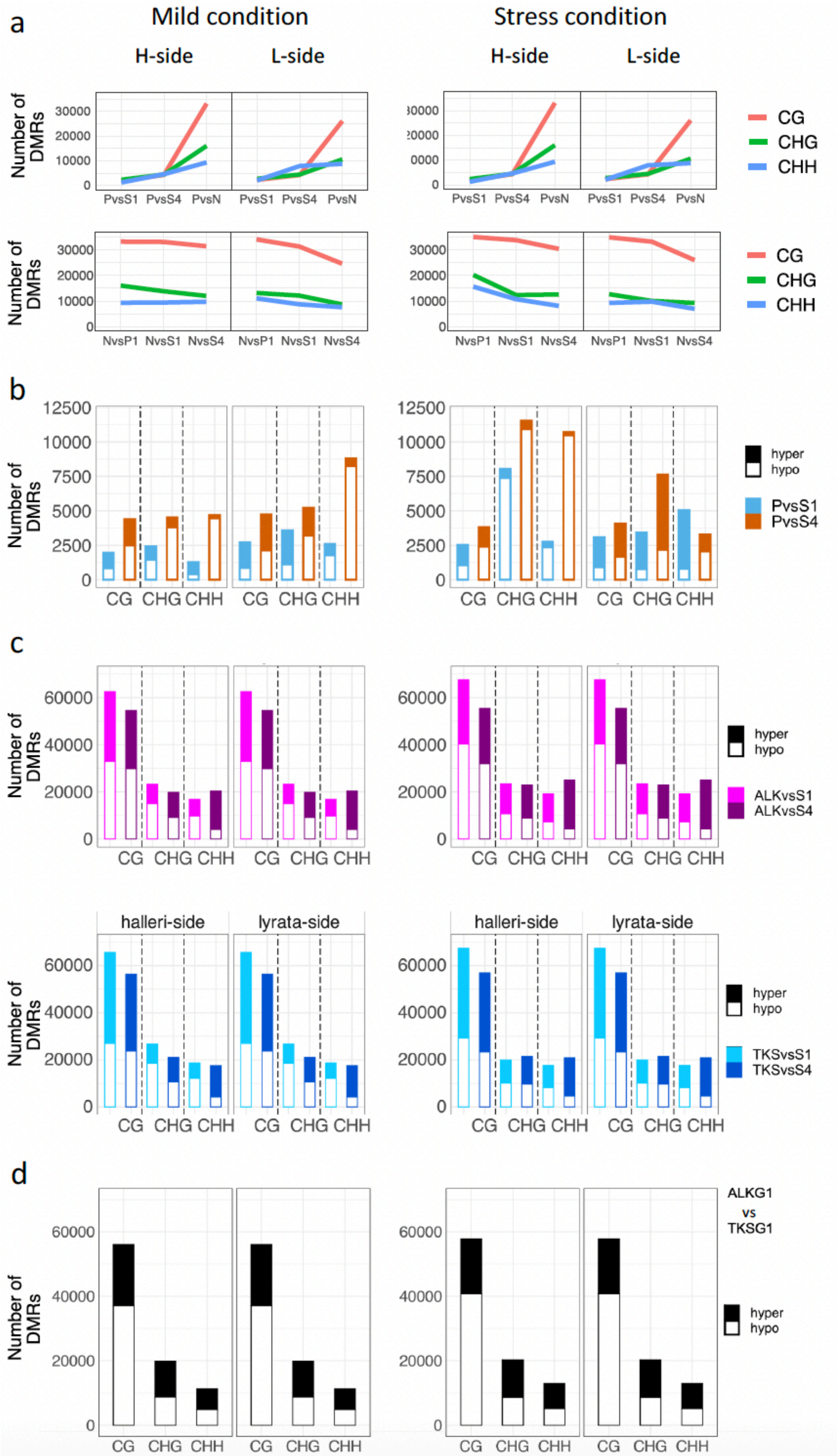

a
Mild condition
Stress condition
H-side
L-side
H-side
L-side
Number of DMRs
PvsS1 PvsS4 PvsN
NvsP1 NvsS1 NvsS4
CG
CHG
CHH
b
hyper
hypo
PvsS1
PvsS4
c
ALKvsS1
ALKvsS4
halleri-side
lyrata-side
TKSvsS1
TKSvsS4
d
ALKG1 vs TKSG1

Supplementary Figure 6 | The pattern and the details of the number of DMRs. **a**, Number of DMRs in S1, S4 and N (TKS) compared to P, and that in P1, S1, S4 compared to N (TKS). They are sibling figures to Fig. 4 b and c, which are drawn on the data of ALK as N. **b** to **d,** Compositions of hyper and hypo-methylation in DMRs between progenitors and synthetics G1 or G4 (b), between synthetics and natural lines (ALK and TKS) (c), and between two natural lines (d). Number of DMRs in each context (CG, CHG and CHH) in two conditions (left: mild and right: stress). Hypermethylation refers to the methylation increase in synthetics (b and c) and TKS (d).

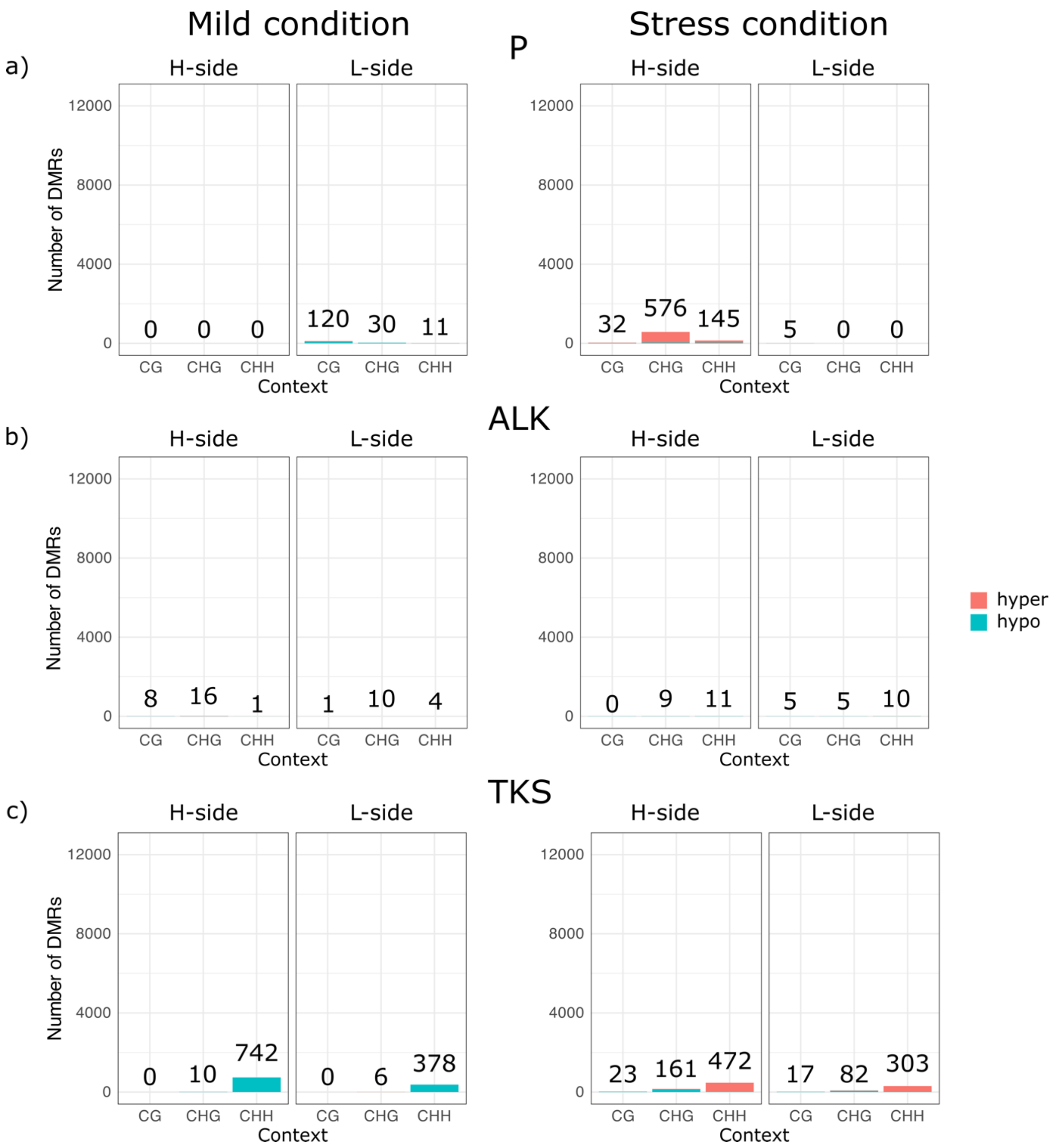

Supplementary Figure 7 | Total DMRs found between G1 and G4. Hyper- and hypo-methylated DMRs at G4 compared to G1 in mild (left) and stress (right) conditions in each subgenome (H-side and L-side) and context (CG, CHG and CHH) in progenitors (a) and natural tetraploids ALK (b) and TKS (c). The y-axis was set the same range as in Fig. S5a to make the comparison easier. For readability the exact number of DMRs is shown at the top of each bar.

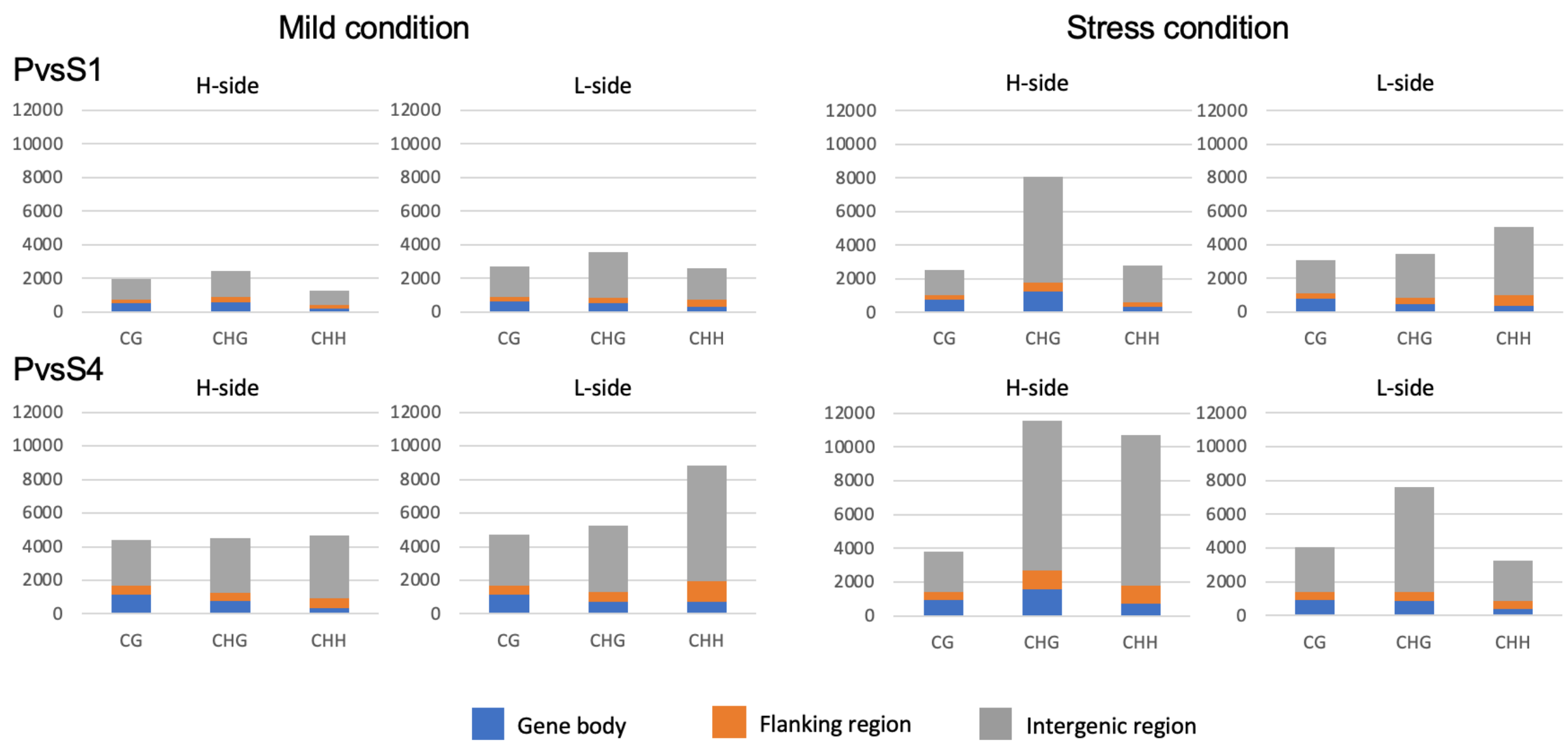

Supplementary Figure 8 | Number of DMRs in three genomic regions. The proportions of DMR in Fig. 5 was converted to bar chart representing the actual numbers in each region.

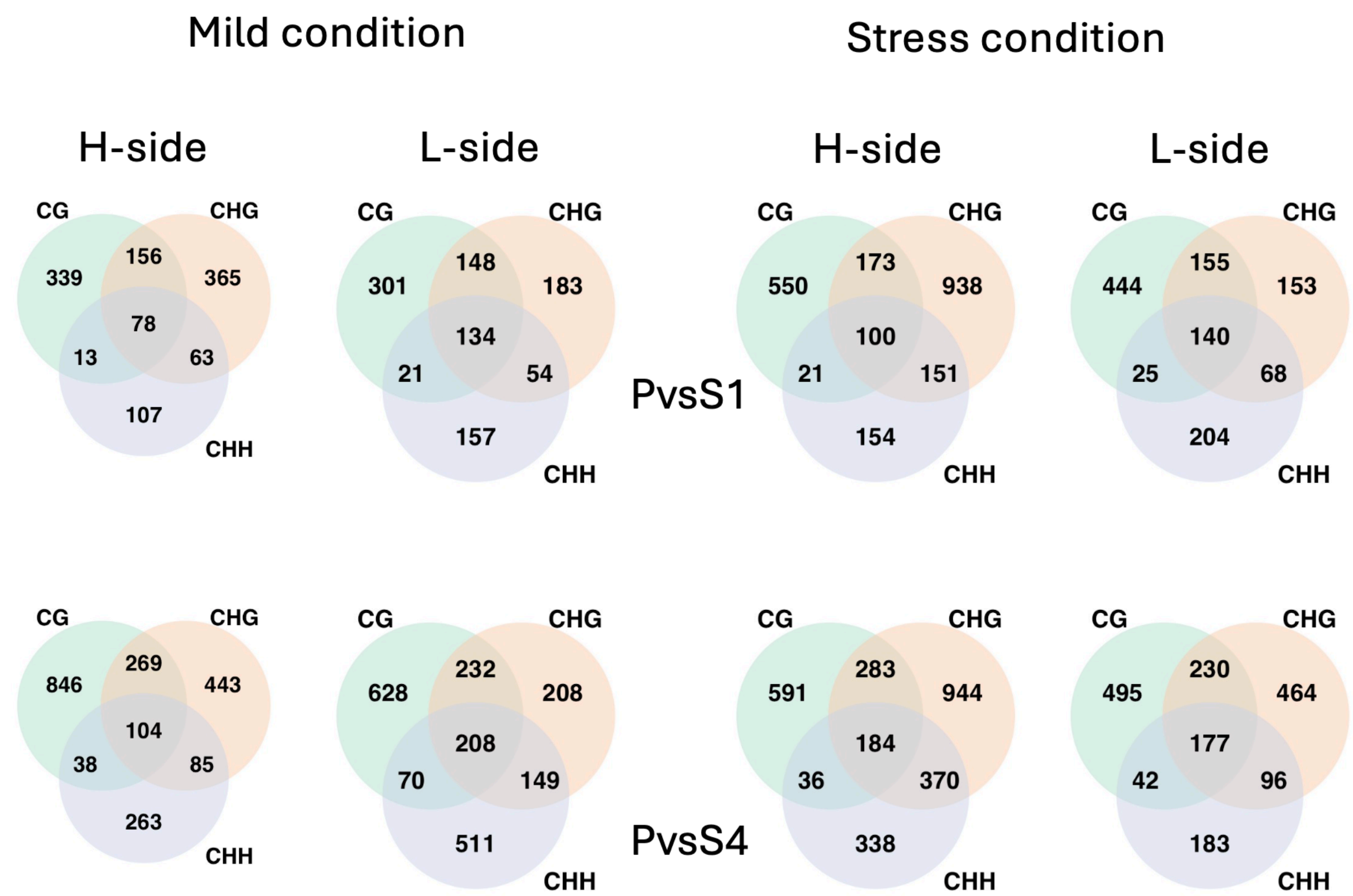

Supplementary Figure 9 | The number of DMGs found in three DNA-methylation contexts in the comparisons between progenitors and synthetic G1 or G4 in each condition. The Venn diagrams represent the number of DMGs detected in each context (CG, CHG, and CHH) DMRs and their overlaps in each comparison. The top and bottom plots are for PvsS1 and PvsS4, and mild and stress conditions are shown on the left and right, respectively.

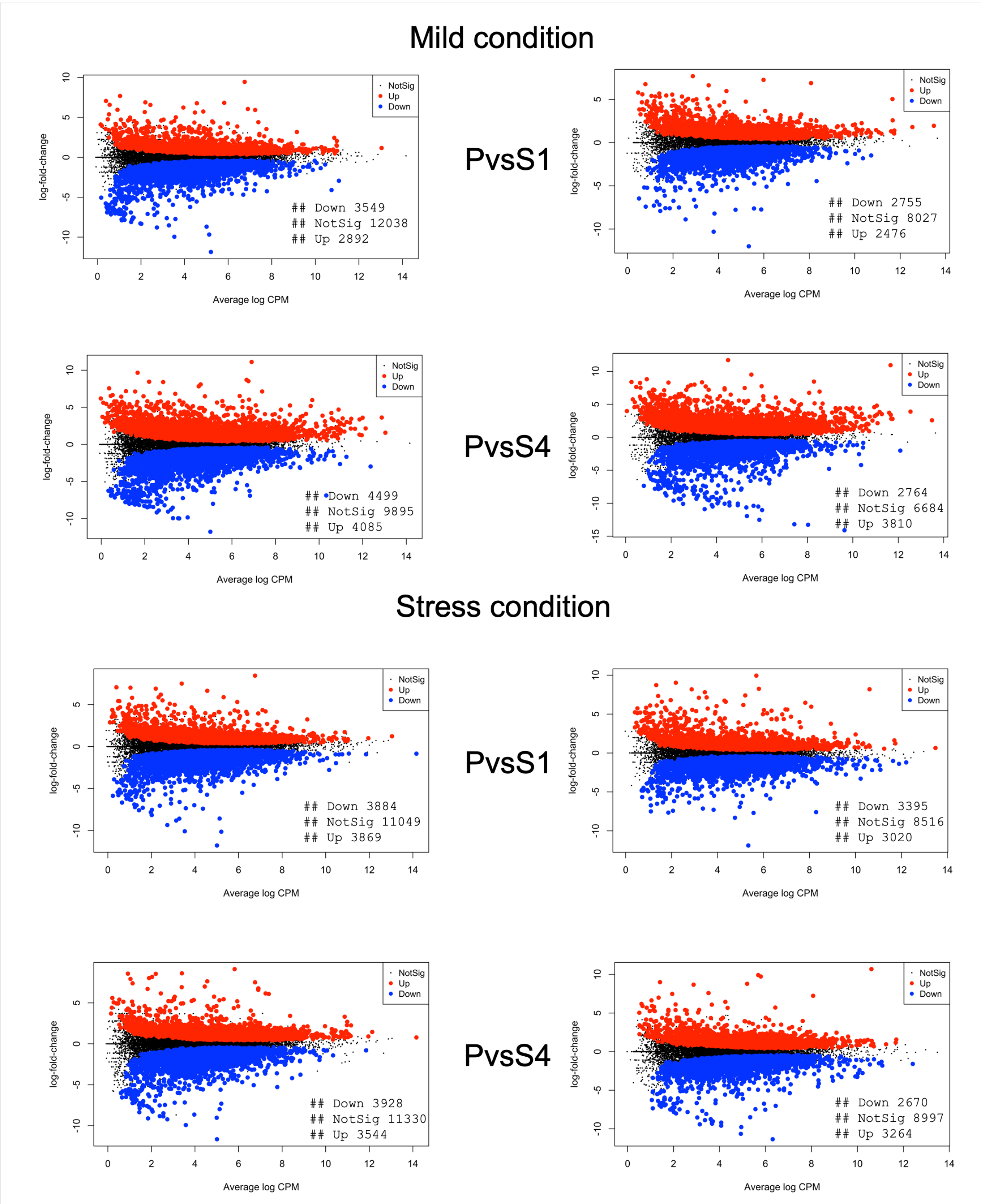

Supplementary Figure 10 | The gene expression pattern of synthetics in G1 and G4 against G1 progenitors as a reference in mild and stress conditions. The top and bottom plots are for mild and stress conditions, and H- and L-sides are shown on the left and right, respectively. Red and blue dots represent significantly up- and down-regulated genes in synthetics, respectively, while black dots represent the genes showing no expression change. The number of genes for each category is noted at the bottom right of each plot.

Mild condition

PvsS1

H-side

χ^2		Expression change	
		Yes	No
Methylation change	Yes	243	878
	No	5974	14423

X-squared = 29.965, df = 1, p-value = 4.398e-08

L-side

χ^2		Expression change	
		Yes	No
Methylation change	Yes	205	793
	No	4867	13730

X-squared = 15.356, df = 1, p-value = 8.9e-05

PvsS4

H-side

χ^2		Expression change	
		Yes	No
Methylation change	Yes	535	1513
	No	7796	11674

X-squared = 151.29, df = 1, p-value < 2.2e-16

L-side

χ^2		Expression change	
		Yes	No
Methylation change	Yes	538	1468
	No	5800	11789

X-squared = 30.898, df = 1, p-value = 2.719e-08

Stress condition

PvsS1

H-side

χ^2		Expression change	
		Yes	No
Methylation change	Yes	474	1613
	No	6985	12446

X-squared = 145.77, df = 1, p-value < 2.2e-16

L-side

χ^2		Expression change	
		Yes	No
Methylation change	Yes	301	888
	No	5892	12514

X-squared = 23.165, df = 1, p-value = 1.487e-06

PvsS4

H-side

χ^2		Expression change	
		Yes	No
Methylation change	Yes	526	2220
	No	6661	12111

X-squared = 287.13, df = 1, p-value < 2.2e-16

L-side

χ^2		Expression change	
		Yes	No
Methylation change	Yes	327	1360
	No	5564	12344

X-squared = 100.15, df = 1, p-value < 2.2e-16

Supplementary Figure 11 | Chi-square tests for the overlaps between DEGs and DMGs in all comparisons between G1 and G4 synthetics and G1 progenitors. Each contingency table shows the number of genes showing changes in DNA methylation and gene expression. At the bottom of each table, details of the chi-square test are noted. The top and bottom plots are for mild and stress conditions, and H- and L-sides are shown on the left and right, respectively.

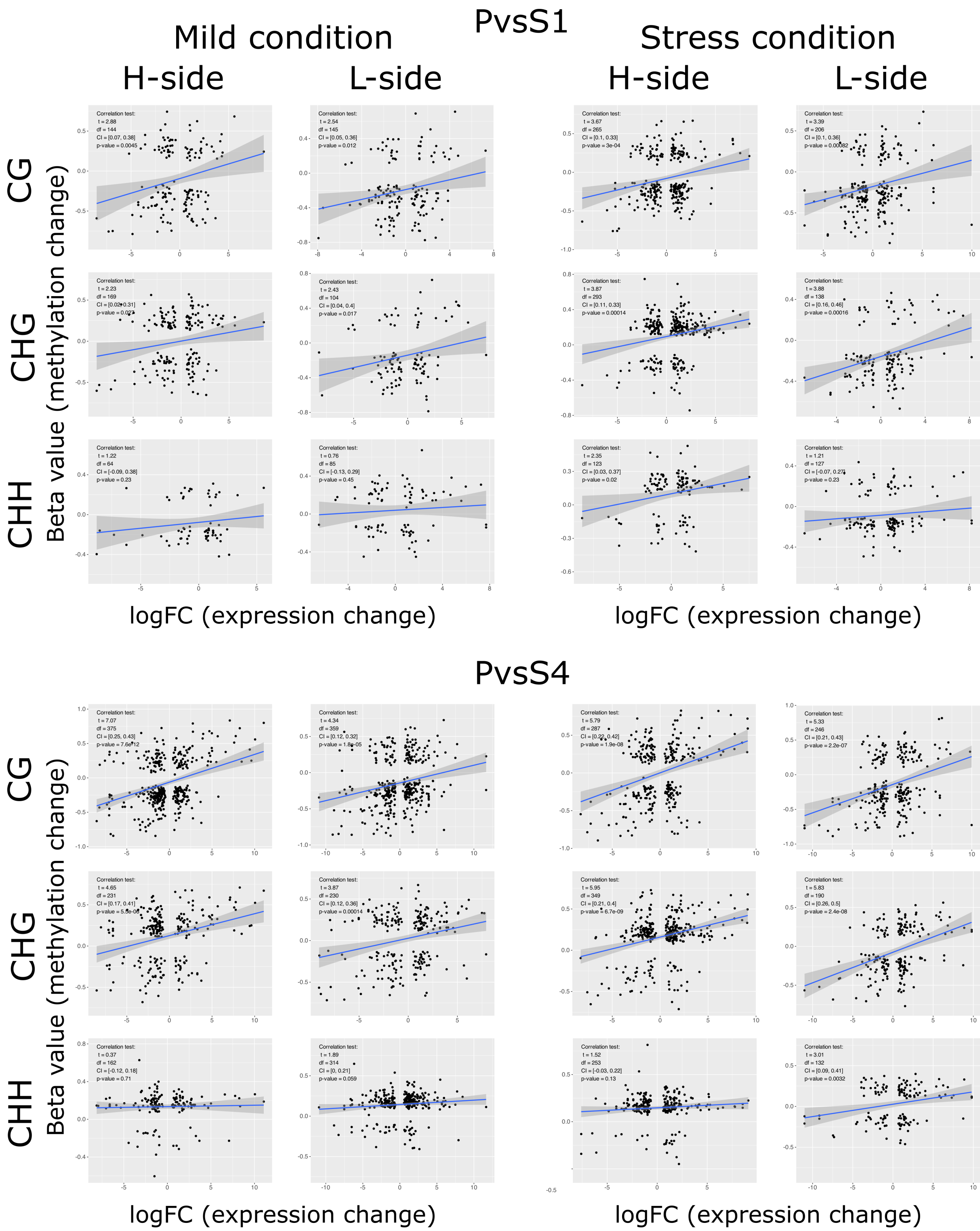

Supplementary Figure 12 | Relationship and correlation between expression and methylation changes in PvsS1 across conditions, subgenomes, and methylation contexts. The genes detected as both DMG and DEG are targeted to

plot the methylation change (change of beta values) of the cytosines included in the gene body on the x-axis and the corresponding gene expression changes in log fold on the x-axis, with each dot representing a gene. The three methylation context context (CG, CHG or CHH) was independently plotted. The beta values refer to the methylation proportion: 0 indicates no methylation and 1 means full methylation. The change of beta values on x-axis ranges from -1 to 1, where the positive value indicates an increase in methylation proportion in synthetics. For each scatter plot, a linear regression was computed with its confidence interval (shaded area around the regression line) together with a Pearson's correlation test with results shown at the upper left of each plot. A significant *p*-value indicates significant correlation between methylation and expression changes. Each column represents a progenitors' side (H or L) at a given condition (mild or stress).

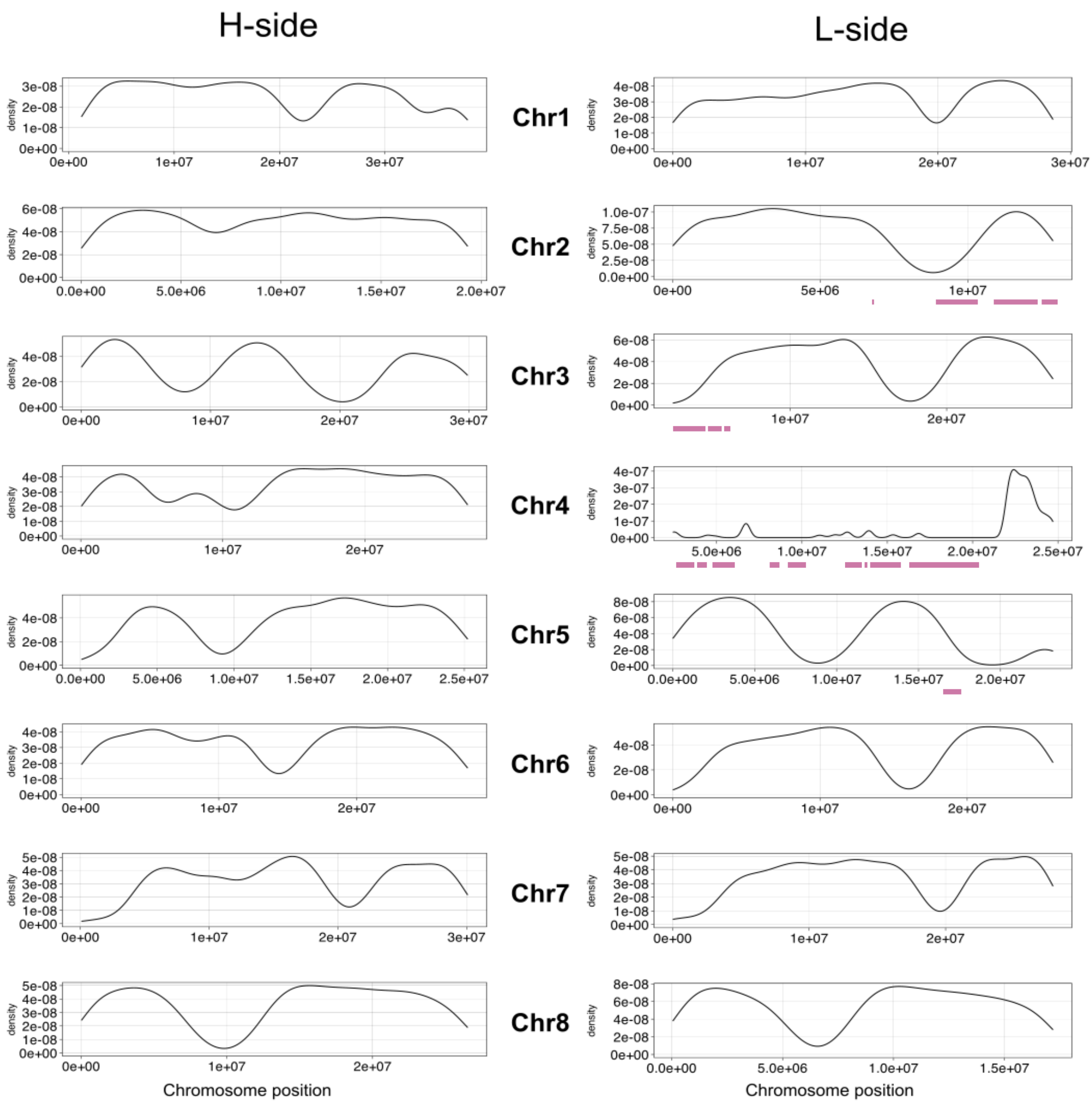

Supplementary Figure 13 | Density of DMRs on each chromosome between synthetic G4 and natural (ALK) in mild condition. Each column represents one progenitor side (H-side and L-side) and each row represents all chromosomes from 1 to 8. Each plot shows the kernel density estimate on y-axis (geom_density() on the ggplot2 R package) and position along the chromosome in bp on the x-axis. The density estimate was calculated using the significant DMRs in our analyses, filtering out any DMRs from the low coverage (HE) regions suggested by pink bars in Fig. S2a. The effect of potential misassemblies of the *A. lyrata petraea* genome at the edge of chromosomes 7 and 8 was not clear, most probably due to the positions.

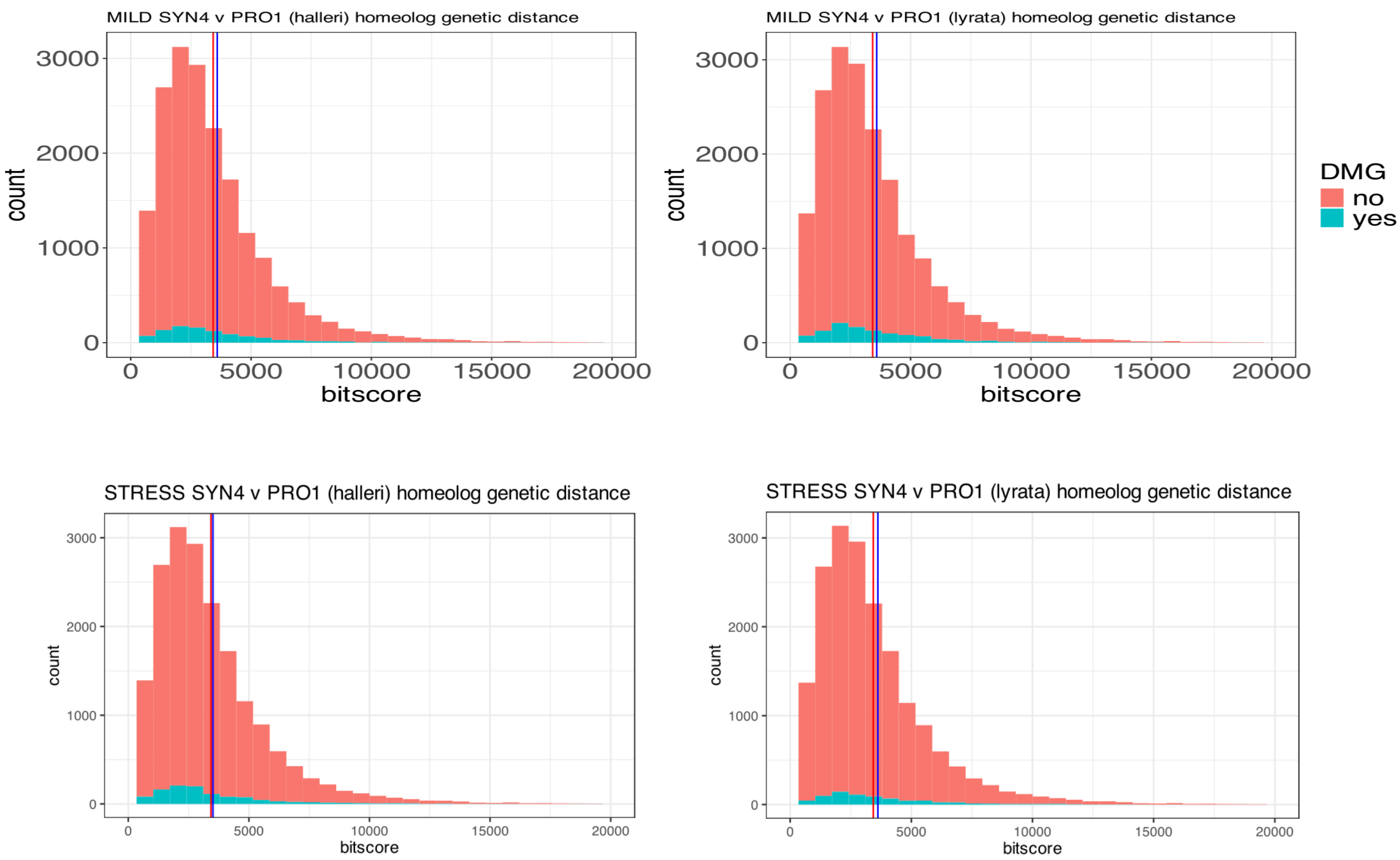

Supplementary Figure 14 | Genetic divergence between homeologous genes and differential methylation in DMG and non-DMG. The genetic divergence of homeologous pairs in coding region was evaluated by BLAST bitscore. The distribution of bitscore was plotted for the homeologous pairs detected as DMGs (blue) and non-DMGs (red) between progenitors (left: halleri, right: lyrata) and their corresponding subgenome of the fourth generation of synthetics (PvsS4) in mild conditions (top) and stress condition (bottom). The blue line indicates the average bitscore of DMG homeologous pairs, while the red line indicates that of non-DMG homeologous pairs. Only one comparison (L-side in mild condition, top right) showed a significant difference by *t*-test (p=0.012).
