# Supplementary material for: Convergence and divergence of DNA methylation and gene expression patterns in neopolyploid Arabidopsis kamchatica
Source: Nat Commun. 2026 Jun 30;17:8419. doi: 10.1038/s41467-026-75042-4 (PMC13478183; doi:10.1038/s41467-026-75042-4)
Supplement: Supplementary file 2 [file 41467_2026_75042_MOESM2_ESM.pdf]

Peer Review File

Convergence and divergence of DNA methylation and gene expression patterns in neopolyploid Arabidopsis kamchatica

Corresponding Author: Dr Rie Shimizu-Inatsugi

This file contains all reviewer reports in order by version, followed by all author rebuttals in order by version.

Version 0:

Reviewer comments:

Reviewer #1

(Remarks to the Author)
The major aim of this manuscript was to investigate the combined effects of polyploidization and environment on DNA methylation and gene expression in the short-term. For this purpose, the authors first assembled high-quality genomes of one accession of each of the two diploid progenitor species, Arabidopsis halleri and A. lyrata, which are the exact parents of a synthetic allotetraploid used in this study. Then, authors examined the methylome and transcriptome changes (both in total scale and subgenome-level) in the synthetic allotetraploid, together with two accessions (adapted to different habitats) of the natural allotetraploid (A. kamchatica), and the two progenitor species, under two environmental conditions. Authors made several interesting observations such as a major effect of allopolyploidization on both DNA methylation and gene expression, divergent changes in DNA methylation under different environmental conditions, and convergence of DNA methylation in the synthetic allotetraploids with progression of generations towards that of the natural allotetraploid species, A. kamchatica. The study topic is interesting and complementary to what is already known in the field. The contents are suited to a journal with readers of broad interest. The manuscript overall is well-written with easy to follow logic. However, I find the analyses are overtly crude and superficial leaving the data largely underexplored. Many places of the analyses require extensive confirmation or clarification. More critically, more in-depth exploration of the functional implications of the changed DNA methylation patterns both with respect to immediate adaptation to whole-genome doubling and short- and long-term adaptation to the environmental conditions should be endeavored. Also, no mechanistic insights underpinning the changes in the DNA methylation and gene expression (although briefly speculated for gene expression changes) are provided. Apart from these general deficiencies, I have the following specific comments and concerns, which hopefully may enable further improvement of the manuscript.
-Lines 116-120: Authors said that the synthetic tetraploids showed lower survival rates and decreased reproductive success under the stress condition. What about the other genotypes, including the natural tetraploids and progenitor species? Also, I could not find any information indicating survival rates in Figure S1. This set of information is critical. For example, if one or both accessions of the natural tetraploid species (A. kamchatica) turned out to be non-tolerant to the stress condition (i.e., showing greatly lowered fitness that the synthetics), then, either the changes did not make sense (inconsequential to environmental adaptation) or the real progenitors of A. kamchatica at the time of formation of the natural tetraploid are substantially different from the still extant diploid species. Also, related to this concern, how the differential adaptation of the two chosen accessions of A. kamchatica to their habitats are related to the conditions chosen for the study? If there is no presumed correlation (between short-term epigenetic changes and long-term adaptation to specific environmental conditions), what is the rational to choose the two specific accessions of he natural tetraploid species? All these information is missing.
-Lines 130-142: Authors observed regions with extremely low coverage on the A. lyrata subgenome in all synthetic samples and attributed this to homeologous exchange (HE)-mediated complete alternate allele replacement (i.e., 4:0). However, HE would inevitably result in a range of homeologous ratios (0:4, 1:3, 2:2, 3:1, 4:0) in early-generation synthetic tetraploids; it is unlikely (totally impossible in G1) to only observe only a 0:4 or 4:0 ratio in the early generations of all individuals. This raises concerns of the analytical details, and reliability of the results. For example, during the mapping process, such as the treatment of multiple hits, might lead to lower coverage in some genomic regions. Authors should verify this by mapping an in silico hybrid/tetraploid using equal amounts of parental data. Furthermore, I suggest the author to examine HEs, resequencing these samples and quantifying the homeologous ratios of chromosomal windows using methods from previous studies. There are several published studies on the topic.
-Lines 147-154: Authors found whole-genome methylation patterns of the synthetic subgenomes clustered with the diploid progenitors and diverged from those of the natural tetraploid species. This observation is not particularly meaningful, as the

synthetics inherited genetic and epigenetic information directly from the two progenitors (parents), while the progenitor species have evolved independently from the natural tetraploid species for a long time. Authors noted that synthetic G4 became slightly closer to ALK and TKS compared to G1, particularly under the stress condition. However, MDS is primarily a dimensional reduction approach and is not suitable for quantifying relationships between samples. For quantification, pairwise comparisons and hierarchical clustering should be conducted.
-Lines 155-161: Why did authors use imputed cytosine calls to estimate global methylation levels? Does this method outperform others? Additionally, I could not find the conversion rates for any sample in the manuscript. I suggest calculating the weighted methylation level using the method proposed by Schultz et al. (2012).
- Lines 233-224: “Counterintuitively, DMGs and DEGs were inconsistent, as the overlaps were significantly smaller than random in both conditions”. If the overlaps are not significantly different from that expected by random, it indicates DMGs and DEGs are not directly correlated, and this makes sense. But what is meant by “significantly smaller than random”?
-What is the size of the DMRs? How many DMGs were homeologous pairs? Was GO:0009408 significantly over-represented? For GO enrichment analysis, the adjusted p-value, rather than the raw p-value, should be used due to multiple comparisons. What was the adjusted p-value for the significantly over-represented GO terms? The range of the y-axis should be reduced in Figure 4.
-Some figures are difficult to understand, such as Figure 6. Additionally, some data analysis procedures are not clearly described and lack of details. For example, how were the WGBS and RNA-seq data of diploid progenitors analyzed? Were they mapped and analyzed as in silico tetraploids using equal data from both diploid parents? How was the reference genome set for the analysis? Was an in silico tetraploid genome constructed using both Arabidopsis halleri genome assembly v2.2 and A. lyrata genome assembly v2.2 as the reference genome?
-In Fig. 4, only the methylation differences identified in the progenitor species and the two accessions of the natural allotetraploid, ALK and TKS, at G1 and G4, are shown. Comparisons between the natural and the synthetic allotetraploids of both generations should be shown as these are central contents of the manuscript.

Reviewer #2

(Remarks to the Author)
The authors constructed a good model to study the evolutionary trajectories of neopolyploids. Based on their analyses of DNA methylation and gene expression in synthetic neo-tetraploids, natural tetraploids, and their progenitors, they identified similar patterns of change. Specifically, they found that neo-tetraploids evolved away from their progenitors and closer to natural tetraploids. Notably, this change was primarily autonomous following polyploidization. Such convergent patterns may reflect similar survival pressures experienced by both synthetic and natural A. kamchatica. The paper is well-organized, though a few questions require clarification.

1, The authors assembled the genomes of two progenitors and found a high syntenic relationship between them, with some exceptions on several chromosomes. However, I observed that genomic structural variations are primarily present in the new assembly of A. lyrata subsp. petraea, while A. lyrata MN47 exhibited a very high syntenic relationship with A. halleri, without inter-chromosome rearrangements. For instance, A. lyrata MN47 and A. halleri displayed intact synteny on chromosome 1, while both showed a translocation between chromosomes 1 and 2 when compared with A. lyrata subsp. petraea. Additionally, a translocation between chromosomes 7 and 8 was identified among A. lyrata subsp. petraea, A. lyrata MN47, and A. halleri. I suspect there may be errors in the assembly of A. lyrata subsp. petraea, but I could not confirm this due to the absence of Hi-C contact maps. The authors should carefully review their genome assembly for A. lyrata subsp. petraea. Furthermore, all raw sequencing data should be uploaded to a public database, and accessible links should be provided for verification of results and future use.

2, T Some figures are difficult to interpret or contain errors. In Figure 1a, the arrow labeled PvsS1 indicates the comparison between the progenitor A. halleri and the halleri subgenome of the synthetic tetraploids. However, PvsS1 should also include comparisons with A. lyrata subsp. petraea and the lyrata subgenome of the synthetic tetraploids, which is not clearly represented. In Figure 2b, the different shapes and colors represent various samples, but the shapes can easily be confused with the error bars. It would be more effective to use different colors to distinguish samples and provide a clearer box diagram.

3, It is surprising that there are no differentially methylated regions (DMRs) observed when comparing the different treatments (Mild vs. Stress) in synthetic G1. Many studies have shown that stress treatments alter methylation and expression patterns. The authors should provide additional evidence to support the validity of their results. Moreover, the WGBS and RNA-seq workflows uploaded to GitHub are currently inaccessible (https://github.com/supermaxiste/EnvironmentalStressPolyploidEvolution).

4, The authors presented a strong discussion, but I believe two additional aspects should be included or strengthened. Firstly, the results indicate a rapid and intense autonomous change in methylation and expression post-polyploidization. Is it possible that these changes correlate with the rebuilding of genome stability necessary for survival? Secondly, there is no significant accumulation of environment-specific genes between the different treatments. One possible reason is that the considered "stress" condition may not represent a sufficiently harsh environment for the synthetic tetraploid. Evidence from natural A. kamchatica shows stable or decreased methylation levels under stress conditions and increased methylation levels under mild conditions, suggesting that the considered "stress" condition may be a weak stressor for the tetraploid.

5, Line 137 incorrectly refers to figure S3; please correct it to refer to the appropriate figure.

Reviewer #3

(Remarks to the Author)
The authors present a study of the dynamic rewiring of methylation patterns in the model allopolyploid Arabidopsis kamchatica, using natural and resynthesized accessions under different environmental stresses.
The progenitors of resynthesized accessions are assembled in high-quality genomes presented in this work, including the assembly of the A. halleri genome, which can be very valuable for the scientific community. However, large non-syntenic regions between A. halleri and A. lyrata could be an assembly artifact of A. halleri genome since no Hi-C data was used, which could be mentioned. Overall, it is not problematic for the paper, where the SVs are not the point of, and the short reads of methylation data will be mapped to such assembly without issues.
The pattern of convergence of methylation from neopolyploids to stable natural polyploids is very interesting. It would also be interesting to discuss the mechanisms of such convergence. When analyzing the methylation patterns, comparing them to the genetic distance would be good. For example, is the dynamics of DMRs somehow related to the genetic distance between the homeologs, or if the convergence of specific methylation patterns can be explained by the closer genetic distance of these regions between the synthetic and natural accessions?
In lines 267-268 you also write “Transcriptome showed a quicker pattern of convergence toward natural polyploids over generations than DMRs” In this case what could be the mechanism?
On Figure 4 you present a comparison of methylation levels in synthetics opposed to progenitors and natural accessions. Did you compare methylation between the two natural A. kamchatica and parents? Are there some DMRs that could be tetraploid-specific and are present both in natural and synthetic A. kamchatica in contrast to the progenitors? Are there specific regions on which methylation levels converge in natural and synthetic G4 A. kamchatica?
The paper could be also placed better in the context of literature. For example, It is worth mentioning epigenetic rewiring under stress conditions after polyploidization observed in tetraploid rice (Wang et al, 2021). The paper also describes two interesting observations. First, the spontaneous doubling of one of the four resynthesized diploid hybrids leading to fertile allotetraploid used in the study. I think it would make sense to mention a similar observation made in Capsella-pursa pastoris (Bachmann et al, 2021, as well as a study suggesting this mechanism in Arabidopsis suecica origin (Burns et al, 2024). Second, the high amount of potential homeologous exchanges in the resynthesised A. kamchatica and their relatively limited amount in the natural accessions. Similar observations were mede in A. suecica, which authors mention. Together they suggests meiotic stabilization of A. suecica (Burns et al 2024, Cheron et al 2023) and A. kamchatica and could be discussed briefly.

Data availability:
Make sure that the github repository is available

Notes on the figures:
Figure 1b : why not using a newer corrected version of MN47 reference (Kolesnikova et al 2023)?

Figure 1d is a bit hard to read, here are some suggestions: indicate each chromosome number and subgenome on each part of the circos plot. For each layer of the plot indicate species and generation instead of 1 to 10. Make figure bigger.

Figure 2c: Lines are overlapping, it is hard to distinguish colors and dashed lines. I would suggest not mixing dashed and normal lines in one plot.

Missing supplementary figure: “In natural tetraploids ALK and TKS, we did not detect a sign of HE by coverage (Fig. S3).”

Fig. 4 why such large y-axis if maximum number of DMR-s goes to less than 1000

Figure 5: Proportions on the circular plot are hard to read and compare. I would suggest using stacked bars instead of circles. Proportions are overall very similar. A text description could be enough, the figure could be moved to supplementary data.

Notes on Materials and Methods:
Line 487-489: Where were A. lyrata and A. halleri collected, coordinates, are they from different populations? A. kamchatica originated from multiple crosses between A. lyrata and A. halleri. Are these A. lyrata and A. halleri potential progenitors to any of the natural A. kamchatica they are compared to?

Line 544: What kind of TE library was used? Reference?

Other notes on the text:

Title: ..in neopolypIoid Arabidopsis kamchatica (remove “of”)

l50: Allopolyploidization? Polyploidization might not involve merging of two genomes (autopolyploidization, after improper segregation of chromosomes) Rephrase the definition of polyploidy?
l61 genetic
l71 which findings?

Version 1:

Reviewer comments:

Reviewer #1

(Remarks to the Author)
I am sorry to say that I feel a bit disappointed at the revisions. The revised manuscript addresses some of my previous comments. However, there are still significant deficiencies in the in-depth exploration of data, functional relevance, and mechanisms, as noted in my earlier report. There remains a lack of rigorous evidence to support serval of the main conclusions.

Lines 116-126: How was the survival rate evaluated? How many replicates were used, and was the difference statistically significant?

Lines 120-121: "More than half of the synthetics produced seeds in mild conditions, but more than half failed to produce seeds in stress conditions." What did this vague statement mean? Need to provide the exact proportions along the text and indicate statistical significance if it meant to highlight differences in seed production between the two conditions.

Lines 170-174: "Synthetics in G4 showed a decrease from G1 for both mild and stress conditions, suggesting convergence from progenitor levels toward natural lines' levels." Please include the global methylation levels of all replicates of each genotype (or the mean and standard deviation) in Figure 3b. Paradoxically, authors also said the synthetics showed divergent changes under different conditions, so under which condition do the synthetics converge to their natural counterpart?

I still do not understand why imputed cytosine calls are used for calculating global methylation levels, considering that global methylation can be calculated for each cytosine context across all sites.

Lines 177-226: DNA methylation is divided into three sequence contexts: CG, CHG, and CHH, each regulated by different pathways and exhibiting different global methylation levels. Typically, DMRs and DMGs should be calculated separately for each cytosine context using different thresholds and presented or discussed separately. Which context is the "DMR with at least 10% methylation difference" referring to? Which categories—CG, CHG, CHH, and hyper- or hypo-methylation—do the DMRs and DMGs in this section pertain to?

I cannot find any information in Figure 4a.

Was GO:0009408 associated with an adjusted p-value less than 0.05? If so, it should be listed with the adjusted p-value.

In this revised version, the authors have not addressed why various homeologous ratios (0:4, 1:3, 2:2, 3:1, 4:0) were not detected in early-generation synthetic tetraploids.

In studies involving hybrids or allopolyploids, in silico hybrids/polyploids (created by mixing equal amounts of sequencing data from both parental species) are often used to minimize biases in analysis, such as read mapping. I recommend that the authors consider using this method, if feasible, to validate the HEs and DMRs detected in synthetic samples.

In the revised version, the authors now state that "DMGs and DEGs were not associated, as the overlaps were significantly smaller than statistically expected in both conditions by chi-square test (Fig. S10)." From a statistical point of view, however, an overlap that is significantly smaller than expected under a random model would usually be interpreted as a negative association / under-representation—that is, DMGs are less likely to be DEGs than expected by chance, which implies a certain degree of avoidance of overlap.

Reviewer #2

(Remarks to the Author)
I have no further conern.

Reviewer #3

(Remarks to the Author)
The authors address all my previous comments sufficiently.
Based on one of my questions, the authors added “In addition, the divergence between the two natural polyploids was not small and only slightly less than the DMRs between synthetic and natural polyploids (Fig. S5d), indicating the influence of independent evolution at their respective habitats since their origins.”
Which is interesting, however the shared origin of the two natural accessions needs to be shown somehow. Since A. kamchatica has multiple hybridization events founding the species, the difference you see might be inherited from the progenitors and not due to environmentally-driven divergence of the pattern. I'm sure that the two accessions are actually coming from the same original natural cross, but it would be good to show it somehow. Maybe they have the same combination of S-alleles? Or they could choose to write this part in the discussion and suggest the two possible explanations: different origins or divergence of methylation patterns after a single origin.

Version 2:

Reviewer comments:

Reviewer #1

(Remarks to the Author)
After going through the itemized responses, the revised manuscript and the suplemental data, I consider the authors have reasonably addressed all my major comments to the previous versions. The concerned points have been calarified and the manuscript has been further improved. I do not have furhter concerns.

Reviewer #3

(Remarks to the Author)
I have no further concerns

Open Access This Peer Review File is licensed under a Creative Commons Attribution 4.0 International License, which permits use, sharing, adaptation, distribution and reproduction in any medium or format, as long as you give appropriate credit to the original author(s) and the source, provide a link to the Creative Commons license, and indicate if changes were made.
In cases where reviewers are anonymous, credit should be given to 'Anonymous Referee' and the source.
The images or other third party material in this Peer Review File are included in the article's Creative Commons license, unless indicated otherwise in a credit line to the material. If material is not included in the article's Creative Commons license and your intended use is not permitted by statutory regulation or exceeds the permitted use, you will need to obtain permission directly from the copyright holder.
To view a copy of this license, visit https://creativecommons.org/licenses/by/4.0/

Response to reviewers comments
We thank all reviewers for their valuable feedback.
Please find the answer to each comment below.
In addition to the modification according to the reviewers comments, we also corrected other typos, and improved some other wording as highlighted in the manuscript (file name: Manuscript_NatCom_withcorrection.pdf)

Reviewer #1 (Remarks to the Author):

The major aim of this manuscript was to investigate the combined effects of polyploidization and environment on DNA methylation and gene expression in the short-term. For this purpose, the authors first assembled high-quality genomes of one accession of each of the two diploid progenitor species, Arabidopsis halleri and A. lyrata, which are the exact parents of a synthetic allotetraploid used in this study. Then, authors examined the methylome and transcriptome changes (both in total scale and subgenome-level) in the synthetic allotetraploid, together with two accessions (adapted to different habitats) of the natural allotetraploid (A. kamchatica), and the two progenitor species, under two environmental conditions. Authors made several interesting observations such as a major effect of allopolyploidization on both DNA methylation and gene expression, divergent changes in DNA methylation under different environmental conditions, and convergence of DNA methylation in the synthetic allotetraploids with progression of generations towards that of the natural allotetraploid species, A. kamchatica. The study topic is interesting and complementary to what is already known in the field. The contents are suited to a journal with readers of broad interest. The manuscript overall is well-written with easy to follow logic. However, I find the analyses are overtly crude and superficial leaving the data largely underexplored. Many places of the analyses require extensive confirmation or clarification. More critically, more in-depth exploration of the functional implications of the changed DNA methylation patterns both with respect to immediate adaptation to whole-genome doubling and short- and long-term adaptation to the environmental conditions should be endeavored. Also, no mechanistic insights underpinning the changes in the DNA methylation and gene expression (although briefly speculated for gene expression changes) are provided. Apart from these general deficiencies, I have the following specific comments and concerns, which hopefully may enable further improvement of the manuscript.

Thank you very much for your precious and insightful comments and suggestions. In the previous version of the manuscript, we focused more on the description of the basic analysis than on a deeper interpretation. According to your and other reviewers' suggestions, we have added new analyses of the features of DNA methylation pattern, focusing on the structural and genetic differences between homeologs, as listed below. By the new analyses, we showed the universal distribution of DMR on chromosomes and non-biased methylation change in homeologous pairs regardless of the genetic distance between them. We have also added descriptions related to each new analysis, which should aid in deeper interpretation.
Fig. S2b: mapping coverage of non-BS DNA-seq to show the consistenty to BS DNA-seq
Fig. S3: mapping coverage of BS DNA-seq of natural tetraploids to show the lack HE

Fig. S5: Proportion of hyper and hypomethylation in DMRs in Fig.4 and the new comparisons between two natural tetraploids.
Fig. S11: distribution of DMR along chromosomes (not biased) in synthetic tetraploids.
Fig. S12: Effect of genetic diversity between homeologous genes (not biased by genetic diversity).
Table. S5: Detailed information of DMR (length distribution, number of included Cs etc.)
Table. S6: Number of homeologous DMG pairs in synthetic tetraploids.
Table. S7: Detailed locality information of the plants
Table. S8: BS DNA-seq information (conversion rate, mapping rate etc.)

In addition to new figures and tables, we have modified the main text and the legends of many figures and improved the illustrations of figures to make them more readable. We also added a new paragraph about the trend of methylation change of synthetic tetraploid in discussion (L 364-374) according to the new analyses (Fig. S11, S12, and Table S5), and another paragraph about the immediate evolution of selfing in neopolyploids and its effect on evolution (408-416).
(L 364-374) We investigated the trend of methylation change in synthetic *A. kamchatica* compared with progenitors by focusing on the following three features to examine how they could shape the susceptibility to change. Firstly, the distribution of DMRs along each chromosome (Fig. S11) was similar among chromosomes, with the valleys linked to the telomeres and centromeres as well as HE regions. Second, the genetic divergence from its homeologous gene did not have a strong correlation to whether the gene was detected as DMG or not (Fig. S12). At last, the number of homeologous pairs both of which were detected as DMGs at S1 and S4 was statistically higher than expected, especially in CG and CHG contexts (Table S6), implying unknown reciprocal effects after polyploidization in specific homeologous pairs. This finding contrasts with the independent evolution of homeologous nucleotide sequences found in the genomes of natural tetraploids of *A. kamchatica* [22].
(408-416) In addition to the epigenetic and transcriptomic change of new synthetic polyploid, we have seen another interesting phenotype in reproduction. As found in other newly synthetized polyploids in *Capsella bursa-pastoris*[47] and *A. suecica*[18], our synthetic tetraploid autonomously turned to be self-fertile immediately after polyploidization. This should be attributed to the combination of *S*-loci between two progenitors: one selfing progenitor with dominant but mutated *S*-locus and another outcrossing progenitor with recessive *S*-locus as revealed in natural poolyploids of *Capsela*[47], *A. suecica*[48], and *A. kamchatica*[49]. These immediate shifts to selfing can contribute to the establishment of new polyploids to establish themselves as new independent species.

-Lines 116-120: Authors said that the synthetic tetraploids showed lower survival rates and decreased reproductive success under the stress condition. What about the other genotypes, including the natural tetraploids and progenitor species? Also, I could not find any information indicating survival rates in Figure S1. This set of information is critical. For example, if one or both accessions of the natural tetraploid species (A. kamchatica) turned out to be non-tolerant to the stress condition (i.e., showing greatly lowered fitness that the

synthetics), then, either the changes did not make sense (inconsequential to environmental adaptation) or the real progenitors of A. kamchatica at the time of formation of the natural tetraploid are substantially different from the still extant diploid species.

We added the detailed information about the survival and fertility level, such as the number of individuals incubated in each generation, the number of surviving individuals until the reproductive stage, and the number of fertile individuals at each generation, in Fig. S1. We modified the main text so that it better explains the difference in survival seed-setting among species and between conditions:
(L116) The survival rate of the synthetic polyploids in the stress condition, which was harsher with the maximum temperature of 26 ℃, was less than 80% (Fig. S1), while 100% for other species. The mild condition is a suitable environment for all three species, with the maximum temperature of 22℃, resulting in 100% survival rates of all species. Next we examined the seed production as a proxi of reproductive success. More than half of the synthetics produced seeds in mild condition, but more than half failed to produce seeds in the stress condition. In addition, in the stress condition, not all individuals of a natural tetraploid ALK derived from a colder habitat produced seeds, while all individuals of the other natural tetraploid, TKS, produced seeds on both conditions. These data suggest the difference in tolerance to the stress condition among natural genotypes. Overall, the fitness of synthetics was affected by the abiotic stress in the stress condition.

Also, related to this concern, how the differential adaptation of the two chosen accessions of A. kamchatica to their habitats are related to the conditions chosen for the study? Suppose there is no presumed correlation (between short-term epigenetic changes and long-term adaptation to specific environmental conditions). What is the rational to choose the two specific accessions of he natural tetraploid species? All these information is missing.

Thank you very much for pointing out this important point. We assumed that the difference between the two natural lines is mainly derived from their respective contrasting natural environments: a relatively mild and cold climate in North America of ALK, and a harsh summer on the sandy lake coast of TKS, besides the difference in progenitors' genotypes, as these two natural tetraploids have separate origins of polyploidization. We presumed that TKS should have an advantage in our stress condition compared to ALK due to the nature of their habitats, resulting from long-term adaptation. This difference is also consistent with the lower success rate of seed-setting of natural tetraploid ALK in stress condition (main text L122 and Fig. S1), reflecting the weaker tolerance due to their colder habitats. We added this sentence to the previous paragraph. (L111-113)
"ALK and TKS are collected at cold habitat and hot habitat of the broad distribution range of *A. kamchatica*, respectively [21]."

The epigenetic difference between these two populations should be reflected in the results of MDS plots on the y-axis. Despite our prediction and expectation, we could not detect the significant differences between the two synthetics at G4 and two natural tetraploids as written in the Discussion (L307-324).

-Lines 130-142: Authors observed regions with extremely low coverage on the A. lyrata subgenome in all synthetic samples and attributed this to homeologous exchange (HE)-mediated complete alternate allele replacement (i.e., 4:0). However, HE would inevitably result in a range of homeologous ratios (0:4, 1:3, 2:2, 3:1, 4:0) in early-generation synthetic tetraploids; it is unlikely (totally impossible in G1) to only observe only a 0:4 or 4:0 ratio in the early generations of all individuals. This raises concerns of the analytical details, and reliability of the results. For example, during the mapping process, such as the treatment of multiple hits, might lead to lower coverage in some genomic regions. Authors should verify this by mapping an in silico hybrid/tetraploid using equal amounts of parental data.

> Accurate mapping is also the most important issue for us. To ensure mapping accuracy, we used ARPEGGIO (ref. 76) developed for BS-DNA reads of polyploids by our group and collaborators.
> First of all, AEPEGGIO uses EAGLE-RC (ref. 80) in its read classification step, which showed the highest mapping accuracy for datasets of allopolyploid species in a benchmarking study (https://doi.org/10.1093/bib/bbaa035). The EAGLE-RC mapping strategy is independent mapping onto two subgenomes and the decision of a read's origin based on statistical comparison, which enabled the high accuracy in classification.
> In addition, we have already evaluated the reliability of our method (ARPEGGIO, ref 76) by counting mismapped reads on the counterpart subgenome using multiple progenitors' (species) datasets. ARPEGGIO achieved lower mismapping (approximately 30%) compared to a conventional method using concatenated subgenomes as a reference. The mismapping rates for the combination of BS-DNA reads of *A. halleri* and *A. lyrata* were 1.65% and 1.45%, respectively (ref. 76). As this evaluation was conducted using the previous versions of genome assembly (more fragmented), the mismapping rates with the current improved versions of genome assembly could be even lower. Considering these facts, we would propose that our mapping method should achieve the currently highest reliability even with BS-treatment.

Furthermore, I suggest the author to examine HEs, resequencing these samples and quantifying the homeologous ratios of chromosomal windows using methods from previous studies. There are several published studies on the topic.

> We sequenced the original (non-BS-treated) DNA as suggested and mapped the new datasets by the same workflow to compare them with BS-treated samples by producing a similar plots of coverage (Fig. S2b). By comparing the BS-treated and non-BS-treated results, the same regions were found as low-coverage regions (Fig. S2b, L143-144), suggesting a negligible effect by BS treatment for our mapping method.
> In the previous papers (ex. doi/10.1111/nph.20095, doi/10.1002/advs.202004222, doi.org/10.1038/s41467-021-25681-6) detecting HE, they specified potential HE regions manually, i.e., by comparing mapping coverages between two homeologous chromosomes as well as between the target region and neighboring/whole regions. To

our knowledge, no conventional and authorized automated method or pipeline currently exists for specifying the HE regions and the ratio of chromosomes.
At last, our first motivation to detect the low-coverage regions was to eliminate the regions with lower coverage that could drastically affect the result due to the smaller number of reads in order to make the result accurate. The lower coverage could be derived by multiple reasons, including HE of whichever unusual ratios, difficulty in mapping, or repetitive sequence. These difficult or low-quality regions were omitted to ensure main purpose of analyzing a general whole genome pattern of methylome transition rather than precisely specifying the causal reasons for the low-quality regions including HE. As a result, HE looked to be a major reason for the low coverage by seeing the contrast of coverage between subgenomes (higher on the counterpart).

-Lines 147-154: Authors found whole-genome methylation patterns of the synthetic subgenomes clustered with the diploid progenitors and diverged from those of the natural tetraploid species. This observation is not particularly meaningful, as the synthetics inherited genetic and epigenetic information directly from the two progenitors (parents), while the progenitor species have evolved independently from the natural tetraploid species for a long time. Authors noted that synthetic G4 became slightly closer to ALK and TKS compared to G1, particularly under the stress condition. However, MDS is primarily a dimensional reduction approach and is not suitable for quantifying relationships between samples. For quantification, pairwise comparisons and hierarchical clustering should be conducted.

We agree that MDS does not follow a perfect linear relationship. MDS was used as a first exploratory step in our large dataset. In later steps, we also focused on the decrease in similarity between progenitors and synthetics with other approaches. To avoid giving a misleading impression, we rephrased the description about the MDS:
[old] (old L152) The synthetic G4 got slightly closer to ALK and TKS compared to G1, particularly in stress conditions.
[new] (current L165) The synthetic G4 was less overlapping with progenitors compared to G1.

-Lines 155-161: Why did authors use imputed cytosine calls to estimate global methylation levels? Does this method outperform others? Additionally, I could not find the conversion rates for any sample in the manuscript. I suggest calculating the weighted methylation level using the method proposed by Schultz et al. (2012).

Thank you very much for suggesting an improvement to the method's description. We have added more information about the conversion rate etc., as a supplementary table (Table S8).
The paper by Schultz et al. (2012) (now referred to as ref. 87) discussed the features of multiple methods to calculate the whole genome methylation level, emphasizing the advantage of the weighted methylation level precisely reflecting the actual level compared to others. METHImpute's global methylation estimates (ref. 86) is also based on the idea of weighted methylation level. This method was shown to be robust, and the output is very similar to the Schultz et al. (2012) approach, even for low-coverage datasets. The authors carried out extensive comparisons between their method and

weighted methylation levels, showing consistent results on a wide range of plant species (including *Arabidopsis*) and coverages.
The main reason we used METHImpute (and its main benefit) is the availability of methylation levels for all cytosines in the genome as an output supported by a Hidden Markov Model. We used those values to explore average genome-wide methylation levels (circlize plot, Fig. 2) and mean recalibrated methylation levels within and around genes (Fig. 3c). This would have not been possible without this program.
We added a phrase and the paper in the method to clarify this at L688:
which is known to output a whole genome methylation level consistent with the weighted methylation level proposed in Schultz et al. 2012.

- Lines 233-224: "Counterintuitively, DMGs and DEGs were inconsistent, as the overlaps were significantly smaller than random in both conditions". If the overlaps are not significantly different from that expected by random, it indicates DMGs and DEGs are not directly correlated, and this makes sense. But what is meant by "significantly smaller than random"?

We mentioned that the overlaps are not significantly different from what would be expected by random chance, as you mentioned. We have rephrased this part and changed the order of the following two sentences.
[old L233-237] Counterintuitively, DMGs and DEGs were inconsistent, as the overlaps were significantly smaller than random in both conditions (Fig. S10). We found a mild positive correlation between the methylation increase and the expression increase in the overlapping genes (Fig. S11). This suggests the presence of transcriptome controls not directly connected to the DNA methylation change in each genic region.
[new L254-261] Counterintuitively, DMGs and DEGs were not associated, as the overlaps were significantly smaller than statistically expected in both conditions by chi-square test (Fig. S10). This suggests the presence of transcriptome controls not directly connected to the DNA methylation change in each genic region. Still, we found a mild positive correlation between the methylation increase and the expression increase in these overlapping genes, implying an effect of methylation on expression (Fig. S10).

-What is the size of the DMRs?

Thank you for pointing out the paucity of this basic information in the previous manuscript. We have added the statistical details on DMRs as Table S5, and mentioned it in the method (L725-726). The range distribution was approximately from 400 to 1000 bp, without any specific trends related to context, subgenomes, or conditions.

How many DMGs were homeologous pairs?

Thank you very much for the precious hint for a deeper analysis. We checked the overlap of DMGs on H and L, for G1 and G4 and added Table S5. We found significantly more homeologous pairs than expected in each comparison, implying a possible correlation in methylation change in homeologous pairs. This number increased from G1 to G4. Nevertheless, the number is very small (3-62 pairs) and because of that further analyses on the gene functions were not possible. One sentence was added to L370:

At last, the number of homeologous pairs both of which were detected as DMGs at S1 and S4 was statistically higher than expected, especially in CG and CHG contexts (Table S5), implying unknown reciprocal effects after polyploidization in specific homeologous pairs.

Was GO:0009408 significantly over-represented? For GO enrichment analysis, the adjusted p-value, rather than the raw p-value, should be used for GO enrichment analysis due to multiple comparisons. What was the adjusted p-value for the significantly over-represented GO terms?

We used TopGo with the elim and Fisher options which generate adjusted *p*-values, and the threshold was set to 0.05 as written in the method (L747-754). The whole GO list with details (including the *p*-value for each GO category and the list of genes detected in each category) is attached as Dataset 1. Specifically for GO:009408, the *p*-value was significant with *p*=0.045 (4 genes included).

The range of the y-axis should be reduced in Figure 4.

The Figure 4 is now moved to a supplementary figure S6. We set the range of y-axis in this figure to make the comparison easier with the number of synthetic tetraploids in Fig 4 and Fig S6.

-Some figures are difficult to understand, such as Figure 6. Additionally, some data analysis procedures are not clearly described and lack of details. For example, how were the WGBS and RNA-seq data of diploid progenitors analyzed? Were they mapped and analyzed as in silico tetraploids using equal data from both diploid parents? How was the reference genome set for the analysis? Was an in silico tetraploid genome constructed using both Arabidopsis halleri genome assembly v2.2 and A. lyrata genome assembly v2.2 as the reference genome?

We used the same pipeline, ARPEGGIO (ref. 75), for both tetraploids and diploids. In this pipeline, sequence reads are mapped to two subgenomes independently using Bismark, rather than concatenated genomes, to compare the matching rates to each subgenome, and EAGLE-RC determines the origin of each read based on statistical probability. For the diploids datasets, we stopped this procedure at the mapping by Bismark on its own chromosome. I added this to L 658:
for both diploid and tetraploid samples, but for the diploid datasets, the read classification step by EAGLE-RC was abbreviated and the alignment by Bismark for the following steps.
ARPEGGIO includes EAGLE-RC (ref 79) for the mapping step, which ensures high accuracy of short-read mapping. A phrase 'on each subgenome' was added to L662 in the explanation of EAGLE-RC. The whole process is relatively conservative, and the proportion of the mapped reads per total number is not necessarily high (20-30% for each subgenome), suggesting 30%< reads are eliminated as 'unclear' reads. The mapping statistics are added as Table S8.

In addition, we improved some other descriptions (figure caption, etc.) as well as figure presentations to make them easier to interpret. In Fig. 6, for example, I added a few terms to explain the type of comparisons on each panel.

-In Fig. 4, only the methylation differences identified in the progenitor species and the two accessions of the natural allotetraploid, ALK and TKS, at G1 and G4, are shown. Comparisons between the natural and the synthetic allotetraploids of both generations should be shown as these are central contents of the manuscript.

The comparisons between the natural G1 and synthetic G1 and G4 were shown in the previous Fig. 3a, in the current Fig. 4.
We also modified Fig. 3 (current Fig. 4) and added a new supplementary figure (Fig. S5) to include more information about the transition of patterns over generations of synthetics: divergence from progenitors (Fig. 4b) and convergence toward natural tetraploid (Fig. 4c).

Reviewer #2 (Remarks to the Author):

The authors constructed a good model to study the evolutionary trajectories of neopolyploids. Based on their analyses of DNA methylation and gene expression in synthetic neo-tetraploids, natural tetraploids, and their progenitors, they identified similar patterns of change. Specifically, they found that neo-tetraploids evolved away from their progenitors and closer to natural tetraploids. Notably, this change was primarily autonomous following polyploidization. Such convergent patterns may reflect similar survival pressures experienced by both synthetic and natural A. kamchatica. The paper is well-organized, though a few questions require clarification.

1, The authors assembled the genomes of two progenitors and found a high syntenic relationship between them, with some exceptions on several chromosomes. However, I observed that genomic structural variations are primarily present in the new assembly of A. lyrata subsp. petraea, while A. lyrata MN47 exhibited a very high syntenic relationship with A. halleri, without inter-chromosome rearrangements. For instance, A. lyrata MN47 and A. halleri displayed intact synteny on chromosome 1, while both showed a translocation between chromosomes 1 and 2 when compared with A. lyrata subsp. petraea. Additionally, a translocation between chromosomes 7 and 8 was identified among A. lyrata subsp. petraea, A. lyrata MN47, and A. halleri. I suspect there may be errors in the assembly of A. lyrata subsp. petraea, but I could not confirm this due to the absence of Hi-C contact maps. The authors should carefully review their genome assembly for A. lyrata subsp. petraea. Furthermore, all raw sequencing data should be uploaded to a public database, and accessible links should be provided for verification of results and future use.

Thank you very much for a valuable suggestion about the genome assembly. We included another genotype of *A. lyrata* (NT1 from Siberia, geographically closer to our *A. lyrata petraea* than MN47) in the synteny analysis (Fig. 1). As a result, some

inconsistencies between *A. halleri* and *A. lyrata petraea* were also supported by NT1, suggesting the intraspecific structural variations in A. *lyrata* (Fig. 1b). On the other hand, the translocation between chromosomes 7 and 8 was not found in NT1, implying a misassembly, which could be improved by adding Hi-C data. We added the description to the manuscript (L137-140). As pointed out also by Reviewer 3, structural variation at the chromosomal level should not basically affect the analysis.

2, Some figures are difficult to interpret or contain errors. In Figure 1a, the arrow labeled PvsS1 indicates the comparison between the progenitor A. halleri and the halleri subgenome of the synthetic tetraploids. However, PvsS1 should also include comparisons with A. lyrata subsp. petraea and the lyrata subgenome of the synthetic tetraploids, which is not clearly represented. In Figure 2b, the different shapes and colors represent various samples, but the shapes can easily be confused with the error bars. It would be more effective to use different colors to distinguish samples and provide a clearer box diagram.

Thank you very much for pointing out the problems with the figures. We improved Fig. 1a to avoid misleading by separating the arrowheads and the circles representing plants, and improved the legend. We modified Figure 2b (current Fig3b) to use the same symbol with different colors to make the error bars clearer.

3, It is surprising that there are no differentially methylated regions (DMRs) observed when comparing the different treatments (Mild vs. Stress) in synthetic G1. Many studies have shown that stress treatments alter methylation and expression patterns. The authors should provide additional evidence to support the validity of their results. Moreover, the WGBS and RNA-seq workflows uploaded to GitHub are currently inaccessible (https://github.com/supermaxiste/EnvironmentalStressPolyploidEvolution).

We carefully checked the analysis again and found out the data support this conclusion. In short, the method we applied (dmrseq, ref. 85) uses a 2-step approach: 1) select candidate regions with at least 10% methylation difference, and 2) check for significant methylation difference. Thanks to this approach, we could break down the number of initial differences (candidate, by 1st step) and the ones that were statistically significant (significant, by 2nd step) (see the table attached below). As an example, in the CHG context, the number of regions with at least 10% difference was almost double in G1 (26’071) compared to G4 (14’818), yet none of them were statistically significant. Two key factors are used to detect significance (see dmrseq paper for details): covariance of nearby loci and biological variability among biological replicates. Because of this, either the high variation in methylation among G1 synthetics replicates or the covariance of adjacent loci in each G1 synthetic sample could have driven this result, as the larger change of methylation pattern was detected at the first generation after the polyploidization (as discussed in L384-386).
To be clearer on the lack of DMRs in synthetic G1, we added the following:
[New L181-L183] At G1, we found a number of difference in two conditions, but none of them was found statistically significant.

	CG	CHG	CHH	
S1 HMvLL	2'571	26'071	4'756	Candidate regions
S1 HMvLL	0	0	0	Significant regions
S4 HMvLL	9'051	14'818	11'335	Candidate regions
S4 HMvLL	1'279	1'422	2'442	Significant regions

The GitHub folder is now open and publicly accessible.

4, The authors presented a strong discussion, but I believe two additional aspects should be included or strengthened. Firstly, the results indicate a rapid and intense autonomous change in methylation and expression post-polyploidization. Is it possible that these changes correlate with the rebuilding of genome stability necessary for survival? Secondly, there is no significant accumulation of environment-specific genes between the different treatments. One possible reason is that the considered "stress" condition may not represent a sufficiently harsh environment for the synthetic tetraploid. Evidence from natural A. kamchatica shows stable or decreased methylation levels under stress conditions and increased methylation levels under mild conditions, suggesting that the considered "stress" condition may be a weak stressor for the tetraploid.

Thank you very much for your valuable suggestion.
For the first point, we agree that the autonomous change, or the epigenetic stabilization, could be one of the keys to successful polyploidization. But it is still hard to discuss how the change contributes to fitness at this point, given the relatively small sample sizes in each group and the lack of a precise physiological evaluation of tolerance (please also see the answer to Reviewer #1's question on Lines 116-120). We added the phrase 'If so, this stabilization could be associated with the successful establishment of a neopolyploid as a new species' at L279 in the discussion.

For the second point, we have found in total 46 GOs (10 on H-side and 36 on L-side) in two conditions at G4 but only one GO category (GO:0009408 response to heat, on L-side) looked directly connected to the difference of condition. Nevertheless, due to the nature of GO analysis, more genes detected as DMG could be involved in the divergence between two conditions even if it was not detected as GO category. First of all, we rephrased the previous description (in L188-190) to make it clearer by adding this information.
In addition, the GMLs of natural tetraploids at two conditions are similar on H-side but those on L-side are slightly higher at stress condition (Fig. 3b), implying a higher sensitivity of the lyrata subgenome for high temperature due to its colder habitat. Nevertheless, the definitive pattern (simple increase or decrease) of GML at stress conditions is still controversial, with examples reporting two opposite patterns. Thus, it is challenging to assess the strength of stress solely from the direction or level of methylation change under the current situation. In addition to the strength of selection, the number of generations could be another factor affecting the speed of change. Thus, we would like to emphasize that the combination of multiple factors (stress level, number of generations, structural limitation of methylation change per generation, etc.) could affect the speed of change of the methylation pattern. In this line, we added the phrase 'The speed of change at each generation should be affected

by multiple reasons, including the strength of stress level and other structural constraints' at L322-324.

5, Line 137 incorrectly refers to figure S3; please correct it to refer to the appropriate figure.

We have corrected the figure numbers according to the new order.

Reviewer #3 (Remarks to the Author):

The authors present a study of the dynamic rewiring of methylation patterns in the model allopolyploid Arabidopsis kamchatica, using natural and resynthesized accessions under different environmental stresses.
The progenitors of resynthesized accessions are assembled in high-quality genomes presented in this work, including the assembly of the A. halleri genome, which can be very valuable for the scientific community. However, large non-syntenic regions between A. halleri and A. lyrata could be an assembly artifact of A. halleri genome since no Hi-C data was used, which could be mentioned. Overall, it is not problematic for the paper, where the SVs are not the point of, and the short reads of methylation data will be mapped to such assembly without issues.

Thank you very much for a lot of valuable comments.
We added another important genotype of *A. lyrata* (NT1 from Siberia, geographically closer to our lyrata than MN47) to the synteny analysis (Fig. 1), which revealed more about the intraspecific structural variation of *A. lyrata* and interspecific difference from *A. halleri*. Please refer to the revised manuscript at L132 and the comments to Reviewer 1 for the details.

The pattern of convergence of methylation from neopolyploids to stable natural polyploids is very interesting. It would also be interesting to discuss the mechanisms of such convergence. When analyzing the methylation patterns, comparing them to the genetic distance would be good. For example, is the dynamics of DMRs somehow related to the genetic distance between the homeologs, or if the convergence of specific methylation patterns can be explained by the closer genetic distance of these regions between the synthetic and natural accessions?

We thank for the interesting idea to focus on the genetic distance between homeologous pairs. This might be a critical factor for the methylation change, as higher genetic divergence between two homeologous genes could also mean higher epigenetic difference, and thus, they could affect each other more at polyploidization. We used BLAST bit score as an indicator of genetic distance between two homeologous genes. Due to the difficulty of comparing non-coding regions, we only considered the coding region to run BLAST. The BLAST bit score distributions of DM Homeologs (DMHs) and non-DM Homeologs were very similar. The only significant

result had a very weak effect. These results indicate that the dynamics of DMRs are not directly related to the genetic distance between homeologs, at least for the initial generations of the synthetic polyploid. We assume other factors (adjacent region, small RNA, or other epigenetic control from the counterpart genome…) can affect the methylation change on each homeologous gene. A new figure (Fig. S12) and new sentences were added on this line at L368:
Second, the genetic divergence from its homeologous gene did not have a strong correlation to whether the gene was detected as DMG or not (Fig. S12).

In lines 267-268 you also write “Transcriptome showed a quicker pattern of convergence toward natural polyploids over generations than DMRs” In this case what could be the mechanism?

It is a very interesting point: what is the underlying mechanism that achieves this pattern? At this moment, we do not have a clear answer, but we hypothesize that the slight change of methylome may be amplified by transcription by either or both cis- and trans-regulations as well as by successive steps in the cascade of signal or expression network, resulting in larger changes in gene expression than DNA methylation, or other (epi)genetic effects as mentioned at L287-L293. This hypothesis could also be consistent with the larger number of DEGs (30% of genes) than DMGs. In addition, the DNA methylation in non-genic region could have large impact on gene expression pattern besides the DNA methylation on genic region, making the overall effect on gene expression larger than we only found in genic region. We added a new sentence in L292: ‘The DMRs outside genic regions could also contribute to this effect.’

On Figure 4 you present a comparison of methylation levels in synthetics opposed to progenitors and natural accessions. Did you compare methylation between the two natural A. kamchatica and parents?

Thank you very much for the precious suggestion. We added the analysis between two natural tetraploids (current Fig. S5c). The divergence between the two natural polyploids was also large, and the number of DMRs was only slightly less than that between synthetic and natural polyploids, as added to L205-207:
In addition, the divergence between the two natural polyploids was not small and only slightly less than the DMRs between synthetic and natural polyploids (Fig. S5d), indicating the influence of independent evolution at their respective habitats since their origins.

Are there some DMRs that could be tetraploid-specific and are present both in natural and synthetic A. kamchatica in contrast to the progenitors?

The number of DMRs between natural tetraploid and progenitors is so huge, reaching up to 60’000 regions, encompassing ca. 15000 DMGs on each subgenome, which corresponds to approx. 75% of genes on each subgenome. This means that the majority of genes are differentially methylated between progenitors and natural tetraploids, making further comparison less meaningful. We conducted the chi-square test to compare the overlap between the two comparisons (natural vs. progenitor and

natural vs. synthetic, in two conditions and in three methylation contexts), but only found that the overlap was not significantly different from random (data not shown) in most combinations, implying the lack of association.

Are there specific regions on which methylation levels converge in natural and synthetic G4 A. kamchatica?

We plotted the physical position of DMRs on chromosomes to find that the distribution is relatively flat along chromosomes except for centromeres and telomeres as well as potential HE regions with lower coverage (Fig. S11). We did not see the influence of the potential misassemblies of the A. lyrata genome, as their positions were on the edge of chromosomes 7 and 8 (This point is also mentioned in the legend of Fig. S11). In addition, the genetic distance between two subgenomes was not relevant to the level of methylation change as shown in the new analysis (Fig. S12). In summary, the change of methylation levels does not accumulate on specific regions but rather universally throughout chromosomes. These results were added in discussion as a new paragraph (L364-374):
We investigated the trend of methylation change in synthetic *A. kamchatica* compared with progenitors by focusing on the following three features to examine how they could shape the susceptibility to change. Firstly, the distribution of DMRs along each chromosome (Fig. S11) was similar among chromosomes, with the valleys linked to the telomeres and centromeres as well as HE regions. Second, the genetic divergence from its homeologous gene did not have a strong correlation to whether the gene was detected as DMG or not (Fig. S12). At last, the number of homeologous pairs both of which were detected as DMGs at S1 and S4 was statistically higher than expected, especially in CG and CHG contexts (Table S6), implying unknown reciprocal effects after polyploidization in specific homeologous pairs. This finding contrasts with the independent evolution of homeologous nucleotide sequences found in the genomes of natural tetraploids of *A. kamchatica* [22].

The paper could be also placed better in the context of literature. For example, It is worth mentioning epigenetic rewiring under stress conditions after polyploidization observed in tetraploid rice (Wang et al, 2021). The paper also describes two interesting observations. First, the spontaneous doubling of one of the four resynthesized diploid hybrids leading to fertile allotetraploid used in the study. I think it would make sense to mention a similar observation made in Capsella-pursa pastoris (Bachmann et al, 2021, as well as a study suggesting this mechanism in Arabidopsis suecica origin (Burns et al, 2024).

We appreciate your suggestion. We added a few sentences and the suggested references on epigenetic rewiring and stress condition of tetraploid rice in L314 to L320:
This higher plasticity in DNA methylation is consistent with the previous report comparing diploid progenitor rice and a new autotetraploid, in which the higher salt tolerance of tetraploid could be attributed to the higher induction of multiple key stress responsive genes most probably due to the higher plasticity of their DNA methylation in tetraploid[35]. We did not detect such a drastic change in a specific group

of genes, probably because our stress could have been milder and the relevant key responsive genes are less clear for heat stress than salt stress.

We also added one sentence to mention the self-compatibility of our synthetic tetraploid (L106) and a new paragraph about the fertility of allotetraploids in discussion (L408), describing the molecular mechanism and the significance of immediate fertility of new polyploids:

In addition to the epigenetic and transcriptomic change of new synthetic polyploid, we have seen another interesting phenotype in reproduction. As found in other newly synthetized polyploids in *Capsella bursa-pastoris*[47] and *A. suecica*[18], our synthetic tetraploid autonomously turned to be self-fertile immediately after polyploidization. This should be attributed to the combination of *S*-loci between two progenitors: one selfing progenitor with dominant but mutated *S*-locus (*A.* lyrata) and another outcrossing progenitor with recessive *S*-locus (*A. halleri*) as revealed in natural poolyploids of *Capsela*[47], *A. suecica*[48], and *A. kamchatica*[49]. These immediate shifts to selfing can contribute to the establishment of new polyploids to establish themselves as new independent species.

Second, the high amount of potential homeologous exchanges in the resynthesised A. kamchatica and their relatively limited amount in the natural accessions. Similar observations were mede in A. suecica, which authors mention. Together they suggests meiotic stabilization of A. suecica (Burns et al 2024, Cheron et al 2023) and A. kamchatica and could be discussed briefly.

We added a few sentences and the suggested references about the miotic instability and HE, as well as the effect of HE in longer term where we discuss the effect of HE in longer term at L351:

This speculation would be consistent with the synthetic *A. suecica* in which more HE has been found[39], and incorrect recombination partner choice at meiosis was found to be strongly correlated with the frequency of HEs. As discussed in the previous papers on synthetic *A. suecica*[39,40], the higher stability in meiosis, as well as fewer HEs, would be more advantageous in the long term.

Data availability:

Make sure that the github repository is available

Now the GitHub folder is open and accessible.

Notes on the figures:

Figure 1b : why not using a newer corrected version of MN47 reference (Kolesnikova et al 2023)?

We have upgraded Fig 1b, including the genome in the suggested publication (NT1). Genome assembly of NT1 utilizes the assembly data of MN47, but their original localities are distant. Thus, we included both genomes in the new synteny analysis.

Figure 1d is a bit hard to read, here are some suggestions: indicate each chromosome number and subgenome on each part of the circos plot. For each layer of the plot indicate species and generation instead of 1 to 10. Make figure bigger.

Former Fig1d is enlarged as Fig2 and modified it to facilitate visualization.

Figure 2c: Lines are overlapping, it is hard to distinguish colors and dashed lines. I would suggest not mixing dashed and normal lines in one plot.

We changed the dashed lines into solid lines in slightly darker colors in G4/5.

Missing supplementary figure: "In natural tetraploids ALK and TKS, we did not detect a sign of HE by coverage (Fig. S3)."

We appreciate your correction. We have thoroughly corrected the figure numbers according to the new order.

Fig. 4 why such large y-axis if maximum number of DMR-s goes to less than 1000

We intended to make it comparable with the sibling Figure (current Fig. 4 and Fig. S5) by using the same range. This figure is now moved to supplementary Fig. S6, due to the reorganization of figures.

Figure 5: Proportions on the circular plot are hard to read and compare. I would suggest using stacked bars instead of circles. Proportions are overall very similar. A text description could be enough, the figure could be moved to supplementary data.

We appreciate your precious suggestion. We made a new version of the figure with stacked bars, which is attached as Fig. S7.

Notes on Materials and Methods:
Line 487-489: Where were A. lyrata and A. halleri collected, coordinates, are they from different populations? A. kamchatica originated from multiple crosses between A. lyrata and A. halleri. Are these A. lyrata and A. halleri potential progenitors to any of the natural A. kamchatica they are compared to?

We added a new table (Table S6) on the coordinates of the diploids used for crossing and the natural tetraploids.
The diploid progenitors used in this experiment were derived from the same populations as used in our previous study (A. lyrata petraea = lyrpet4/5 and A. halleri= halgem2 in Ref. 11, Shimizu-Inatsugi et al. 2009). Based on the result of phylogenetic analysis using two nuclear single genes in Refs. 10 and 21, the progenitors used in this paper were shown to be one of the closest to natural tetraploids compared to other

populations from Europe or other regions. We added the information in the method section (L560). Especially, an identical (and very similar) gene sequence was found between lyrpet4/5 and some of the Alaskan tetraploid populations. We also find a similar gene sequence between halgem2 and many of Japanese (but not TKS) and Alaskan tetraploid populations, even though no identical sequence was found. These results suggest the close relationship between our diploid lines and the actual progenitors of natural tetraploids.

Line 544: What kind of TE library was used? Reference?

Thank you for pointing out the unclear description. The library in reference 68 was used, as described in L627.

Other notes on the text:

Title: ..in neopolyploid Arabidopsis kamchatica (remove "of")

We appreciate the precious suggestion. We have removed 'of'.

l50: Allopolyploidization? Polyploidization might not involve merging of two genomes (autopolyploidization, after improper segregation of chromosomes) Rephrase the definition of polyploidy?

We rephrased this term as 'Allopolyploidization'. In addition, we added 'allo' to seven other terms (in lines 38, 40, 82, 90, 309, 344, 378, and 388) to make our intention clearer.

l61 genetic

Corrected.

l71 which findings?

Rephrased to 'The findings described above'.

Response to reviewers comments

We thank all reviewers again for their valuable feedback.

Please find the answer to each comment below.

In addition to the changes mentioned in the responses, we also made some minor modifications (e.x, number of Figures). All modifications are highlighted in the manuscript file in red.

Reviewer #1 (Remarks to the Author):

I am sorry to say that I feel a bit disappointed at the revisions. The revised manuscript addresses some of my previous comments. However, there are still significant deficiencies in the in-depth exploration of data, functional relevance, and mechanisms, as noted in my earlier report. There remains a lack of rigorous evidence to support serval of the main conclusions.

Point 1
Lines 116-126: How was the survival rate evaluated? How many replicates were used, and was the difference statistically significant?

The number of incubated, surviving, and fertile individuals were added to Fig. S1 in the previous version. In this version, we also added all the results (*p*-values) of statistical evaluation of the difference in survival and fertility between the two conditions in each genotype using Fisher's exact test (two-sided) as Fig. S1(c), with the summary of the numbers of individuals (fertile/survived). The test was performed on each generation and the sum of all generations (G1 to G4/G5). Due to the small sample size per genotype in each generation, no significant *p*-values were observed within each generation. Nevertheless, a significant difference ($p<0.05$) was detected in the sum of all generations for the fertility of ALK and synthetic tetraploid ($p=0.0445$ and 0.0245, respectively) (See Point 2 below).

Point 2
Lines 120-121: "More than half of the synthetics produced seeds in mild conditions, but more than half failed to produce seeds in stress conditions." What did this vague statement mean? Need to provide the exact proportions along the text and indicate statistical significance if it is meant to highlight differences in seed production between the two conditions.

By Fisher's exact test (two-sided), the fertility of synthetic tetraploids in mild and stress conditions had a significant difference ($p=0.0245$), though the test did not detect significance in each generation due to small sample sizes. In addition to the new Fig. S1(c), we modified the text:
L120(old) *More than half of the synthetics produced seeds in mild condition, but more than half failed to produce seeds in the stress condition.*
(new) *Seed production among all synthetics across generations was significantly different between conditions, better in mild condition (Fig. S1).*

Point 3
Lines 170-174: "Synthetics in G4 showed a decrease from G1 for both mild and stress

conditions, suggesting convergence from progenitor levels toward natural lines' levels." Please include the global methylation levels of all replicates of each genotype (or the mean and standard deviation) in Figure 3b. Paradoxically, authors also said the synthetics showed divergent changes under different conditions, so under which condition do the synthetics converge to their natural counterpart?

The mean and standard deviation of each genotype have been included in Figure 3b (from the 1st version of the manuscript), represented by the symbol and error bars (without caps as end style) . Please note that most of the error bars are masked by the symbols due to their small size (though some are visible).
The divergent change is discussed for two synthetic lines under two conditions, showing more DMRs in G4 than in G1 (Fig. 4a), as described in L180-194.
The convergence is discussed in the results in Fig. 4c, as the number of DMRs between natural and synthetic polyploids gets smaller in G4 than in G1 in both natural tetraploids, and even lower than that between natural progenitors and natural tetraploids. This trend is noticeable in CG context, as described in L 225-232, at both conditions, respectively. The two patterns, divergence between synthetics and the convergence of synthetics toward natural tetraploids, do not necessarily have to be mutually exclusive.

Point 4

I still do not understand why imputed cytosine calls are used for calculating global methylation levels, considering that global methylation can be calculated for each cytosine context across all sites.

As the nature of shotgun sequencing, no mapping or low mapping due to the fluctuation of sequencing coverage is inevitable, and missing data on specific cytosines can occur stochastically in any sequencing, which may affect the classic calculation method assuming sufficient coverage at all cytosines. The advantage of METHimpute is its robustness to fluctuations in sequencing coverage. A Hidden Markov Model algorithm is used in METHimpute to estimate and impute methylation states for all cytosines across the whole genome. Our global methylation level is calculated from this output using the classic method you mentioned (implemented in METHimpute).
As long as we cannot avoid low or no coverage due to shotgun sequencing, this method is essential to conduct the calculation. This approach overcomes the limitations of technical issues of sequencing (dependency on the comprehensiveness of sequence data), assuming the missing data on any cytosines.
After the publication of METHimpute in 2019, this paper has been cited more than 45 times (as of 2026.02). In many cases, it was used to detect whole-genome methylation levels, underscoring the method's usefulness and broad demand.
We further clarified the point in the method section by modifying the sentence:
L701(old) *In short, METHImpute applies a Hidden Markov Model to WGBS data to impute methylation calls and levels for all cytosines in the genome.*
(new) *In short, METHImpute applies a Hidden Markov Model to WGBS data to impute methylation levels for all cytosines in the genome,* ***enabling robust analysis even with low-coverage datasets***.

Point 5

Lines 177-226: DNA methylation is divided into three sequence contexts: CG, CHG, and CHH, each regulated by different pathways and exhibiting different global methylation levels.

Typically, DMRs and DMGs should be calculated separately for each cytosine context using different thresholds and presented or discussed separately. Which context is the "DMR with at least 10% methylation difference" referring to? Which categories—CG, CHG, CHH, and hyper- or hypo-methylation—do the DMRs and DMGs in this section pertain to?

We use dmrseq to detect DMR/DMG, as mentioned in the method (L689) at the first step, which, by default, considers each of the three contexts (CG, CHG, and CHH) independently. For the most analyses, we provided a breakdown of context and hyper-, hypo-methylation in each context.

1st point: Which context is the "DMR with at least 10% methylation difference" referring to?

As explained in our previous response (see below: **The previous comment and response**), we meant this 10% methylation difference in each context, which is the default threshold setting of dmrseq to detect DMR, to simplify the calculation procedure. However, the description in the main manuscript, "DMR with at least 10% methylation difference" might be confusing for the majority of readers who are not aware of this setting of dmrseq. So, we simplified the description at this part, focusing on statistical significance:

> Line181(old) *At G1, we found a number of candidate regions of DMR with at least 10% methylation difference in two conditions, but none of them was found statistically significant.*
> (new) *At G1, **we did not find any statistically significant DMR.***

2nd point: Which categories—CG, CHG, CHH, and hyper- or hypo-methylation—do the DMRs and DMGs in this section pertain to?

In this section, because the number of detected DMGs was small in each context, we summarized all contexts to conduct GO analysis. As we only consider the genes with reciprocal-best-hit orthologs in *A. thaliana*, the number of genes for GO analysis always got lower than the number of detected DMGs. This was not mentioned in the manuscript, so we have added the description.

> L185(old): *There were 225 and 164 DMGs for H- and L-subgenomes (H- and L-sides), respectively (Supplementary Dataset 1), which we used to conduct Gene Ontology (GO) enrichment analysis.*
> (new): *There were **181, 255, and 201 DMGs for CG, CHG, and CHH context, respectively, at G4 on H-side, and 167, 156, and 126 DMGs on L-side (Supplementary Dataset 1). By Gene Ontology (GO) enrichment analysis on each context, we did not find enrichment in temperature-related categories in either group, potentially due to the small sample sizes. Then we combined the DMGs from three categories in each subgenome to target 225 and 164 DMGs with reciprocal best-hit genes in A. thaliana for the H- and L-sides, respectively, by filtering out overlaps (Supplementary Dataset 1).***

Specifically for GO:0009408 (response to heat), 4 genes contributed to the enrichment:
Almed_G015236 (AT1G11270) CHH-down
Almed_G020759 (AT3G47860) CG-down
Almed_G007599 (AT4G14690) CHG-down, CG-down
Almed_G024506 (AT1G11270) CHG-up
(Down means less methylation in stress condition)
All of the detailed information can be found in Dataset 1.

In addition to this section (data shown in Fig. 4a), we analyze DMGs in two other sections (data shown in Fig. 4b and Fig. 8a).
For the data shown in Fig. 4b, we ran GO analysis on each context, and the summary of the results is shown separately in Table S4. To back up this description, we added Fig. S8 newly, a series of Venn diagrams showing the detailed numbers of DMGs in each context and their overlaps in the comparison between progenitors and synthetic G1 or G4.
For Fig. 8, we summarized DMRs in three contexts. Here, the original number of DEGs in each context is much smaller compared to the number of DEGs (5K< as found in Fig. 8a), which makes the comparison between DEGs and DMGs in each context hard due to the extreme size difference. Thus, we combined all DMGs across three contexts with the purpose of making a more general overview comparing DEGs and DMGs.
We added the details how the three contexts were treated in each part, and added the detailed number of DMGs detected in each context as Fig. S8.

Main text regarding Fig 4b.
L217(old) *When we focused on the DMGs, i.e. the genes overlapping with DMRs detected in gene bodies and flanking regions,*
(new) *When we focused on the DMGs, i.e. the genes overlapping with DMRs detected in gene bodies and flanking regions* ***in each context (Fig. S8),***

Main text regarding Fig.8a
L259(old) *To link gene expression and DNA methylation at genes, we examined the overlap between the DMGs and DEGs (Fig. 8a, Supplementary Dataset 3).*
(new*) To link gene expression and DNA methylation at genes, we examined the overlap between the DMGs and DEGs (Fig. 8a, Supplementary Dataset 3)* ***by combining all DMGs across three contexts (Fig. S8) to compare them with DEGs.***

We appreciate you pointing this out, as it gives us the chance to improve the description.

The previous comment and response:

3, It is surprising that there are no differentially methylated regions (DMRs) observed when comparing the different treatments (Mild vs. Stress) in syntheAc G1. Many studies have shown that stress treatments alter methylation and expression patterns. The authors should provide additional evidence to support the validity of their results. Moreover, the WGBS and RNA-seq workflows uploaded to GitHub are currently inaccessible (hNps://github.com/supermaxiste/EnvironmentalStressPolyploidEvoluAon).

We carefully checked the analysis again and found out the data support this conclusion. In short, the method we applied (dmrseq, ref. 85) uses a 2-step approach: 1) select candidate regions with at least 10% methylation difference, and 2) check for significant methylation difference. Thanks to this approach, we could break down the number of initial differences (candidate, by 1st step) and the ones that were statistically significant (significant, by 2nd step) (see the table attached below). As an example, in the CHG context, the number of regions with at least 10% difference was almost double in G1 (26'071) compared to G4 (14'818), yet none of them were statistically significant. Two key factors are used to detect significance (see dmrseq paper for details): covariance of nearby loci and biological variability among biological replicates. Because of this, either the high variation in methylation among G1 synthetics replicates or the covariance of adjacent loci in each G1 synthetic sample could have driven this result, as the larger change of methylation pattern was detected at the first generation after the polyploidization (as discussed in L384-386).
To be clearer on the lack of DMRs in synthetic G1, we added the following:
[New L181-L183] At G1, we found a number of difference in two conditions, but none of them was found statistically significant.

Point 6

I cannot find any information in Figure 4a.

I am not sure whether this comment is about the number of counts in the left figure of 4a, or the figure was not visible for some technical issues. If it is a technical issue, we submitted a newer version of the file. If it is about the number of counts, the number of DMRs at synthetic G1 between two conditions was zero for all contexts, as mentioned at L181 to 183 by the reason in our previous response (please also refer to the response to Point 5).

Point 7

Was GO:0009408 associated with an adjusted p-value less than 0.05? If so, it should be listed with the adjusted p-value.

In the topGO analysis, no global multiple testing correction/adjustment (e.g., FDR) was applied, but an alternative method can be used to avoid false positives. We used this "elim" algorithm, in combination with Fisher's exact test as described in the method (L759), to account for the hierarchical dependency structure of Gene Ontology (GO) terms. The elim procedure iteratively removes genes mapped to significant child terms from their parent terms, thereby reducing the propagation of false positives within the GO directed acyclic graph (DAG). Therefore, the analysis employs a structure-aware strategy to mitigate false-positive enrichment due to GO term dependencies.

We added this information in the Method at L762: ***to reduce the propagation of false positives within the GO directed acyclic graph, and the p-value was calculated using this algorithm***

In addition, to make the targeted gene group(s) for GO analysis clearer, we also added this information at L763 and the same information 'TAIR 10 orthologs (reciprocal best hit)' in Table 1, at the second line from the bottom.

L764 (old) *To assign A. thaliana genes comparable to A. halleri and A. lyrata genes, we checked for ortholog genes based on BLAST reciprocal best hit between genes of our reference and A. thaliana.*

(new) *To assign A. thaliana genes comparable to A. halleri and A. lyrata genes, we checked for orthologous genes based on BLAST reciprocal best hit between genes of our* ***reference (A. halleri or A. lyrata)*** *and A. thaliana* ***(TAIR10), and 20'418 (H-side) and 20'435 (L-side) genes were considered for GO analysis in each subgenome, respectively (Table 1)****.*

Point 8

In this revised version, the authors have not addressed why various homeologous ratios (0:4, 1:3, 2:2, 3:1, 4:0) were not detected in early-generation synthetic tetraploids.

It is likely that all ratios (0:4, 1:3, 2:2, 3:1, 4:0) existed in our samples. For our purpose to study the differential methylation among homeologs, chromosomal regions that lack one of them (0:4 or 4:0) are not usable, and thus we searched for them and removed them from the analysis. We analyzed all other analyzable regions. As the reviewer pointed out, some of the regions may have 1:3 or 3:1 ratio. It will be an interesting future topic to search for such regions

and analyze their potential effect on the methylation pattern, but we suggest that it is beyond the scope of this manuscript.

Given the history of our synthetic tetraploid (hybridization of diploid progenitors and autonomous tetraploidization of an F1 hybrid individual), homeologous exchanges can already occur at the F1 hybrid stage (diploid), meaning that the 0:4 and 4:0 ratios could already be observed in the 1st generation of the synthetic tetraploid. If it happens in a later generation in tetraploids, the other variations could also appear. We added one sentence about these possible HE scenarios in the manuscript:
L352: *The timing of these HE generations and their dynamics remain understudied. In our synthetic tetraploids, HE events may occur at either the diploid hybrid stage or following tetraploid stages, resulting in copy number ratios ranging from 0:4 to 4:0.*

Point 9

In studies involving hybrids or allopolyploids, in silico hybrids/polyploids (created by mixing equal amounts of sequencing data from both parental species) are often used to minimize biases in analysis, such as read mapping. I recommend that the authors consider using this method, if feasible, to validate the HEs and DMRs detected in synthetic samples.

As the nature of our mapping and sorting software (ARPEGGIO and EAGLE-RC included in mapping and sorting step), the mapping and sorting of each read is conducted independently and do not affect those of other reads. Thus, the pooling of equal amounts of reads of two progenitors does not give a different result compared to the independent mapping of the datasets of two progenitors. As written in our previous response, the accuracy of EAGLE-RC was proven to be one of the highest by a benchmarking study, and we also proved the high accuracy of ARPEGGIO in the original paper.

> **The previous response:**
> Accurate mapping is also the most important issue for us. To ensure mapping accuracy, we used ARPEGGIO (ref. 76) developed for BS-DNA reads of polyploids by our group and collaborators. First of all, AEPEGGIO uses EAGLE-RC (ref. 80) in its read classification step, which showed the highest mapping accuracy for datasets of allopolyploid species in a benchmarking study (https://doi.org/10.1093/bib/bbaa035). The EAGLE-RC mapping strategy is independent mapping onto two subgenomes and the decision of a read's origin based on statistical comparison, which enabled the high accuracy in classification.
> In addition, we have already evaluated the reliability of our method (ARPEGGIO, ref 76) by counting mismapped reads on the counterpart subgenome using multiple progenitors' (species) datasets. ARPEGGIO achieved lower mismapping (approximately 30%) compared to a conventional method using concatenated subgenomes as a reference. The mismapping rates for the combination of BS-DNA reads of *A. halleri* and *A. lyrata* were 1.65% and 1.45%, respectively (ref. 76). As this evaluation was conducted using the previous versions of genome assembly (more fragmented), the mismapping rates with the current improved versions of genome assembly could be even lower. Considering these facts, we would propose that our mapping method should achieve the currently highest reliability even with BS-treatment.

Point 10

In the revised version, the authors now state that "DMGs and DEGs were not associated, as the overlaps were significantly smaller than statistically expected in both conditions by chi-square test (Fig. S10)." From a statistical point of view, however, an overlap that is significantly smaller than expected under a random model would usually be interpreted as a negative association / under-representation—that is, DMGs are less likely to be DEGs than expected by chance,

which implies a certain degree of avoidance of overlap.

Thank you very much for pointing out the critical fault in the description. We modified this sentence at L260.
(old) *Counterintuitively, DMGs and DEGs were not associated, as the overlaps were significantly smaller than statistically expected in both conditions by chi-square test (Fig. S10). This suggests the presence of transcriptome controls not directly connected to the DNA methylation change in each genic region.*
(new) *Counterintuitively, DMGs and DEGs were* ***negatively*** *associated,* ***with overlaps*** *significantly smaller than expected* ***by chance*** *in both conditions,* ***as assessed*** *by chi-square tests (Fig. S10).* ***This should reflect a complex correlation between DNA methylation and expression, most likely due to multiple control factors not directly related to DNA methylation****.*

Reviewer #2 (Remarks to the Author):

I have no further concern.

Reviewer #3 (Remarks to the Author):

The authors address all my previous comments sufficiently.
Based on one of my questions, the authors added "In addition, the divergence between the two natural polyploids was not small and only slightly less than the DMRs between synthetic and natural polyploids (Fig. S5d), indicating the influence of independent evolution at their respective habitats since their origins."
Which is interesting, however the shared origin of the two natural accessions needs to be shown somehow. Since A. kamchatica has multiple hybridization events founding the species, the difference you see might be inherited from the progenitors and not due to environmentally-driven divergence of the pattern. I'm sure that the two accessions are actually coming from the same original natural cross, but it would be good to show it somehow. Maybe they have the same combination of S-alleles? Or they could choose to write this part in the discussion and suggest the two possible explanations: different origins or divergence of methylation patterns after a single origin.

Thank you very much for pointing out this critical issue. The origins of two natural polyploids are distinct, as mentioned at Line 109 and as discussed in the previous papers analyzing nuclear genes and chloroplast haplotype (Ref. 10 and 22) and *S*-alleles (https://journals.plos.org/plosgenetics/article?id=10.1371/journal.pgen.1002838). We added this *S*-locus paper as a reference 23, where we mentioned the distinct origins at Line109 (Result).
We have also modified the last part of the suggested sentence to include this information on distinct origins at L211:
(old) *indicating the influence of independent evolution at their respective habitats since their origins.*
(new) *indicating the influence of independent evolution at their respective habitats* ***as well as the distinct progenitors' genotypes at polyploidizations.***

We also added references describing the origins at L572 in the section Plant Material and sequencing in Method as well as in line 111 in Results. Instead, reference 46 was removed because it was the same as reference 42. We also corrected typos in the references.
