# Supplementary material for: Convergence and divergence of DNA methylation and gene expression patterns in neopolyploid Arabidopsis kamchatica
Source: Nat Commun. 2026 Jun 30;17:8419. doi: 10.1038/s41467-026-75042-4 (PMC13478183; doi:10.1038/s41467-026-75042-4)
Supplement: Supplementary file 3 [file 41467_2026_75042_MOESM3_ESM.pdf]

Description of Additional Supplementary Files

File Name: Supplementary Data 1
Description: Basic information about the result of sequencing, mapping, and BS-conversion of each biological sample.
