# Supplementary material for: Convergence and divergence of DNA methylation and gene expression patterns in neopolyploid Arabidopsis kamchatica
Source: Nat Commun. 2026 Jun 30;17:8419. doi: 10.1038/s41467-026-75042-4 (PMC13478183; doi:10.1038/s41467-026-75042-4)
Supplement: Supplementary file 4 [file 41467_2026_75042_MOESM5_ESM.pdf]

nature portfolio

Corresponding author(s): Rie Shimizu Inatsugi

Last updated by author(s): Nov 27, 2025

Reporting Summary

Nature Portfolio wishes to improve the reproducibility of the work that we publish. This form provides structure for consistency and transparency in reporting. For further information on Nature Portfolio policies, see our Editorial Policies and the Editorial Policy Checklist.

Statistics

For all statistical analyses, confirm that the following items are present in the figure legend, table legend, main text, or Methods section.

n/a	Confirmed	
☐	☒	The exact sample size (*n*) for each experimental group/condition, given as a discrete number and unit of measurement
☒	☐	A statement on whether measurements were taken from distinct samples or whether the same sample was measured repeatedly
☐	☒	The statistical test(s) used AND whether they are one- or two-sided<br>*Only common tests should be described solely by name; describe more complex techniques in the Methods section.*
☒	☐	A description of all covariates tested
☒	☐	A description of any assumptions or corrections, such as tests of normality and adjustment for multiple comparisons
☐	☒	A full description of the statistical parameters including central tendency (e.g. means) or other basic estimates (e.g. regression coefficient) AND variation (e.g. standard deviation) or associated estimates of uncertainty (e.g. confidence intervals)
☐	☒	For null hypothesis testing, the test statistic (e.g. *F*, *t*, *r*) with confidence intervals, effect sizes, degrees of freedom and *P* value noted<br>*Give P values as exact values whenever suitable.*
☒	☐	For Bayesian analysis, information on the choice of priors and Markov chain Monte Carlo settings
☒	☐	For hierarchical and complex designs, identification of the appropriate level for tests and full reporting of outcomes
☒	☐	Estimates of effect sizes (e.g. Cohen's *d*, Pearson's *r*), indicating how they were calculated

Our web collection on statistics for biologists contains articles on many of the points above.

Software and code

Policy information about availability of computer code

Data collection: n/a

Data analysis:

- HiFiasm - v.0.14
- Purge Haplotigs - v1.1.1
- BLAST+ - v.2.11.0
- RagTag - v1.1.1
- BUSCO - v.5.2.2
- KAT - v.2.4.1
- MAKER - v.3.01.04
- fastp - v.0.20.1
- HISAT2 - v.2.2.1
- SAMtools - v.1.13
- StringTie2 - v.2.1.7
- gffread - v.0.12.7
- PASA - v.2.5.3
- Trinity - v.2.8.5
- tRNAscan-SE - v.2.0.9
- Infernal - v.1.1.4
- RepeatMasker - v.4.1.2

- GENESPACE - v.1.3.1
- Orthofinder - v.2.5.5
- FastME - v.2.1.6.3
- MCL - v.22-282
- DIAMOND - v.2.1.9
- R - v4.0.2, v4.0.5
- tidyverse - v1.3.0, v1.3.1
- GenomicRanges - v1.40.0, v1.42.0
- TrimGalore - v0.6.5
- Bismark - v0.22.3
- EAGLE-RC - v1.1.2
- QualiMap - v2.2.2d
- MultiQC - v1.8
- dmrseq - v1.6.0
- METHImpute - v1.10.0
- STAR - v2.7.3a
- featureCounts - v2.0.1
- edgeR - v3.32.1
- karyoploteR - v1.16.0
- VennDiagram - v1.7.0
- ARPEGGIO - v2.0.0
- topGO - v2.52.0
- FastQC - v0.11.8
- data.table - v1.13.0
- gridExtra - v2.3

For manuscripts utilizing custom algorithms or software that are central to the research but not yet described in published literature, software must be made available to editors and reviewers. We strongly encourage code deposition in a community repository (e.g. GitHub). See the Nature Portfolio guidelines for submitting code & software for further information.

Data

Policy information about availability of data

All manuscripts must include a data availability statement. This statement should provide the following information, where applicable:

- Accession codes, unique identifiers, or web links for publicly available datasets
- A description of any restrictions on data availability
- For clinical datasets or third party data, please ensure that the statement adheres to our policy

Genomes:
The genome assemblies are registered in NCBI under the BioProject No.
PRJNA788966 (A. halleri)
PRJNA788965 (A. lyrata)

Other datasets are available in DDBJ under the following accession codes:
BS DNA-seq dataset (PRJDB12567)
DNA-seq dataset (PRJDB39671)
RNA-seq dataset (PRJDB12582)

Research involving human participants, their data, or biological material

Policy information about studies with human participants or human data. See also policy information about sex, gender (identity/presentation), and sexual orientation and race, ethnicity and racism.

Reporting on sex and gender	n/a
Reporting on race, ethnicity, or other socially relevant groupings	n/a
Population characteristics	n/a
Recruitment	n/a
Ethics oversight	n/a

Note that full information on the approval of the study protocol must also be provided in the manuscript.

Field-specific reporting

Please select the one below that is the best fit for your research. If you are not sure, read the appropriate sections before making your selection.

☐ Life sciences ☐ Behavioural & social sciences ☒ Ecological, evolutionary & environmental sciences

For a reference copy of the document with all sections, see nature.com/documents/nr-reporting-summary-flat.pdf

Ecological, evolutionary & environmental sciences study design

All studies must disclose on these points even when the disclosure is negative.

Study description	Genome assemblies of two diploid plant species is conducted. The same genetypes were used to produce hybrid F1 plants, and among them one individual tetraploidized autonomously. The offsprings of this individual was incubated in two climate conditions. The whole genome DNA methylation pattern (BS DNA-seq) in the first and fourth generations in each climate condition was analyzed, using mature leaves. The gene expression pattern (RNA-seq) from the same leaf samples were also analyzed. In parallel, the two diploid plants, serving as the progenitors of this synthetic tetraploids and the two genetic lines of two tnatural tetraploids collected from field sites were incubated in the same climate conditions, and the whole genome DNA methylation pattern (BS DNA-seq) and gene expression pattern (RNA-seq) were also analyzed to be compared with the synthetic tetraploids. The genotypes of the two diploid progenitors were chosen as they were found to be the closest to the actual progenitors of natural tetraploids by previous publication among multiple genotypes collected from wide distribution ranges.
Research sample	All genotypes (Arabidopsis lyrata, Arabidopsis halleri, Arabidopsis kamchatica) were obtained from natural field sites with permission if needed and imported to the country where the experiment was conducted (Switzerland) with permission from both exporting and importing countries.
Sampling strategy	A common biological triplicates approach was taken for data collection and analysis.
Data collection	n/a
Timing and spatial scale	n/a
Data exclusions	n/a
Reproducibility	Biological triplicates were set.
Randomization	n/a
Blinding	n/a

Did the study involve field work? ☐ Yes ☒ No

Reporting for specific materials, systems and methods

We require information from authors about some types of materials, experimental systems and methods used in many studies. Here, indicate whether each material, system or method listed is relevant to your study. If you are not sure if a list item applies to your research, read the appropriate section before selecting a response.

Materials & experimental systems

n/a	Involved in the study	
☒	☐	Antibodies
☒	☐	Eukaryotic cell lines
☒	☐	Palaeontology and archaeology
☒	☐	Animals and other organisms
☒	☐	Clinical data
☒	☐	Dual use research of concern
☐	☒	Plants

Methods

n/a	Involved in the study	
☒	☐	ChIP-seq
☒	☐	Flow cytometry
☒	☐	MRI-based neuroimaging

Plants

Seed stocks	All genotypes used in this experiment were originally collected from natural habitats and incubated for multiple generations in our laboratory. The precise locations of the collection are described in the manuscript.
Novel plant genotypes	A new genotype (synthetic tetraploid) was produced in the laboratory by crossing two plants (Arabidopsis lyrata, Arabidopsis halleri) and by its autonomous polyploidization.
Authentication	n/a
