# Supplementary material for: Convergence and divergence of DNA methylation and gene expression patterns in neopolyploid Arabidopsis kamchatica
Source: Nat Commun. 2026 Jun 30;17:8419. doi: 10.1038/s41467-026-75042-4 (PMC13478183; doi:10.1038/s41467-026-75042-4)
Supplement: Supplementary file 5 [file 41467_2026_75042_MOESM6_ESM.zip › Fig 6/ReadMe_GeneList_DEG.rtf]

The following columns in each file refer to:

geneID: Gene ID on A. halleri genome or A. lyrata genome
logFC: fold change of gene expression (CPM) 
logCPM: average CPM between samples, used as x-axis of volcano plot (Fig S4)
PValue: p-value from edgeR’s exactTest
dir: direction of the expression change, 1 for increased and -1 for decreased. For all folders with comparisons XvsY, X is always the reference. Same goes for XvsY/Z/W
qvalue: FDR corrected p-value
