# Supplementary material for: Convergence and divergence of DNA methylation and gene expression patterns in neopolyploid Arabidopsis kamchatica
Source: Nat Commun. 2026 Jun 30;17:8419. doi: 10.1038/s41467-026-75042-4 (PMC13478183; doi:10.1038/s41467-026-75042-4)
Supplement: Supplementary file 5 [file 41467_2026_75042_MOESM6_ESM.zip › Fig 4/ReadMe_GeneList.rtf]

The following columns in each file refer to:
geneID: Gene ID on A. halleri or A. lyrata genome
seqname: Gene location (chromosome)
start: Gene starting position (start codon) 
end: Gene ending position (start codon) 
region_start: DMR start position	
region_end: DMR end position	
overlap_length: Overlap between gene and DMR in bp
methylation_status: methylation increase or decrease in the DMR in target
